# Calliviminone A from *Callistemon citrinus* Induces PANC-1 Pancreatic Cancer Cell Death by Targeting the PI3K/Akt/mTOR Pathway

**DOI:** 10.3390/plants14132074

**Published:** 2025-07-07

**Authors:** Juthamart Maneenet, Ahmed M. Tawila, Hung Hong Nguyen, Nguyen Duy Phan, Orawan Monthakantirat, Supawadee Daodee, Chantana Boonyarat, Charinya Khamphukdee, Yaowared Chulikhit, Suresh Awale

**Affiliations:** 1 Natural Drug Discovery Laboratory, Institute of Natural Medicine, University of Toyama, 2630 Sugitani, Toyama 930-0194, Japan; juthamar@inm.u-toyama.ac.jp (J.M.);; 2 Division of Pharmaceutical Chemistry, Faculty of Pharmaceutical Sciences, Khon Kaen University, Khon Kaen 40002, Thailand; 3 Division of Pharmacognosy and Toxicology, Faculty of Pharmaceutical Sciences, Khon Kaen University, Khon Kaen 40002, Thailand

**Keywords:** *Callistemon citrinus*, calliviminone A, pancreatic cancer, PANC-1, antiausterity, PI3K/Akt/mTOR pathway, phytochemistry

## Abstract

Pancreatic cancer cells exhibit a remarkable ability to tolerate nutrient deprivation, a phenomenon termed “austerity,” which enables their survival within the hypovascular tumor microenvironment. Conventional anticancer therapies frequently fail to effectively target these resilient neoplastic cells, posing a significant challenge to the therapeutic management of pancreatic cancer. Consequently, targeting austerity, the ability of cancer cells to tolerate nutrient starvation, represents a promising anti-austerity strategy for developing novel pancreatic cancer therapeutics. In this study, we investigated calliviminone A (CVM-A), a phloroglucinol–meroterpenoid isolated from *Callistemon citrinus* leaves, for its anti-austerity activity against PANC-1 human pancreatic cancer cells. Calliviminone A exhibited potent preferential cytotoxicity in nutrient-deprived medium (NDM) with a PC_50_ of 0.57 µM, while showing minimal toxicity in nutrient-rich Dulbecco’s Modified Eagle’s medium (IC_50_ = 45.2 µM), indicating a favorable therapeutic index. Real-time live-cell imaging revealed that CVM-A induced significant morphological changes, including cell shrinkage and membrane blebbing, leading to cell death within 24 h of NDM. Furthermore, under normal nutrient conditions in Dulbecco’s Modified Eagle’s Medium (DMEM), CVM-A significantly inhibited PANC-1 cell migration (up to 47% reduction at 20 µM) and colony formation (over 80% suppression at 25 µM), suggesting its antimetastatic potential. Western blot studies demonstrated that CVM-A downregulated key survival components of the PI3K/Akt/mTOR signaling pathway, completely inhibiting Akt and p-Akt at 2.5 µM in NDM, and suppressing insulin-induced Akt activation. These findings highlight CVM-A as a promising lead compound for developing novel anticancer therapies that target the adaptive survival mechanisms and metastatic potential of pancreatic cancer in nutrient-deprived microenvironments.

## 1. Introduction

Pancreatic cancer remains one of the most lethal malignancies, with a 5-year survival rate below 10%, primarily due to late-stage diagnosis, which limits therapeutic options [1,2,3]. In 2025, pancreatic cancer is estimated to be the sixth leading cause of cancer-related deaths worldwide, with approximately 466,000 deaths annually and over 500,000 new cases diagnosed [4,5]. In Japan, pancreatic cancer ranks as the fourth leading cause of cancer-related deaths, with approximately 43,000 deaths annually [6,7]. Surgical resection offers the best prospect for long-term survival, yet only approximately 20% of patients are eligible for advanced disease at presentation [2,8]. Even after successful resection, recurrence is common and often occurs within months, significantly reducing survival rates [9,10]. Adjuvant chemotherapy, typically gemcitabine monotherapy, is a standard post-surgical treatment to reduce the risk of recurrence [11]. Although gemcitabine is the standard first-line treatment for patients with unresectable or metastatic pancreatic cancer, its impact on overall survival remains limited [12,13]. To address this, combination regimens such as FOLFIRINOX (leucovorin, fluorouracil, irinotecan, and oxaliplatin) and gemcitabine-based therapies (e.g., gemcitabine/nab-paclitaxel) have been adopted in clinical practice [14,15]. However, these regimens rarely achieve complete remission, and their increased toxicity often leads to severe side effects, necessitating treatment discontinuation [16,17].

Pancreatic tumors are characterized by aggressive growth and high nutrient demand to sustain rapid proliferation [18,19]. Unlike most cancers, which rely on angiogenesis to secure nutrients, pancreatic tumors are hypovascular, resulting in restricted blood flow and nutrient availability [20,21]. Remarkably, pancreatic cancer cells adapt to this nutrient-scarce microenvironment through metabolic reprogramming, a phenomenon termed “austerity” [22,23]. Targeting adaptive tolerance to nutrient deprivation has emerged as a promising anti-austerity strategy for identifying novel anticancer agents. In this approach, compounds are screened for selective cytotoxicity against pancreatic cancer cells in nutrient-deprived medium (NDM) compared with nutrient-rich Dulbecco’s Modified Eagle’s medium (DMEM). Compounds exhibiting preferential toxicity in NDM without affecting cells in DMEM are classified as anti-austerity agents, with activity quantified by the “Preferential Cytotoxicity” (PC_50_) value, representing the concentration inducing 50% cell death in NDM without toxicity in DMEM [24,25,26]. Previous studies have identified potent anti-austerity agents, including nicolaioidesin C [25,27], arctigenin [22], angelmarin [23], plumbagin derivatives [24], and toyaburgine [28], some of which have demonstrated efficacy in suppressing pancreatic tumor growth in preclinical mouse models [25,26,27,28].

As part of our ongoing efforts to discover novel anti-austerity agents, we previously identified potent activity in the CH_2_Cl_2_ extract of *Callistemon citrinus* leaves, with a PC_50_ value of 7.4 µg/mL. Phytochemical analysis of this extract yielded twenty-nine compounds, including seven novel meroterpenoids (callistrilones L–Q and epicallistrilone Q), which exhibited preferential cytotoxicity against PANC-1 human pancreatic cancer cells [29,30,31]. Among these, calliviminone A (CVM-A), a phloroglucinol–meroterpenoid adduct, displays the most potent preferential cytotoxicity in the nanomolar PC_50_ range. However, detailed biological effects and mechanisms of action remain unexplored. In this study, we conducted a comprehensive biological evaluation of CVM-A and investigated its effects on cell death, morphology, migration, colony formation, and the underlying molecular mechanisms, with a particular focus on the PI3K/Akt/mTOR signaling pathway.

## 2. Results

### 2.1. Anti-Austerity Activity of Calliviminone A (CVM-A) Against PANC-1 Human Pancreatic Cancer Cells

**Figure 1:**
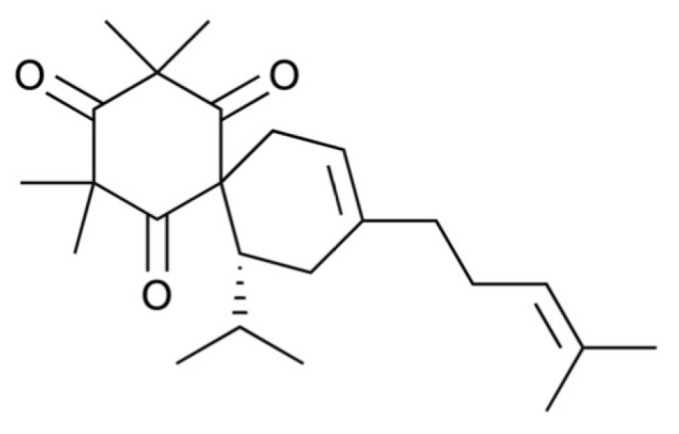
Structure of calliviminone A.

Calliviminone A (CVM-A, Figure 1), a phloroglucinol–meroterpenoid, was isolated from the CH_2_Cl_2_ extract of *Callistemon citrinus* leaves, as previously reported [29,30,31,32] and is detailed in the Supplementary Material. Its structure was elucidated using nuclear magnetic resonance (NMR) spectroscopy, including ^1^H and ^13^C NMR, and validated by comparison with the prior literature [33] (see Supplementary Material, Table S1, Figures S1 and S2 for ^1^H and ^13^C NMR spectroscopic data and spectra). The anti-austerity activity of CVM-A was evaluated in PANC-1 human pancreatic cancer cells cultured in nutrient-deprived medium (NDM) and standard Dulbecco’s Modified Eagle Medium (DMEM). NDM, lacking glucose and serum, mimics the nutrient-scarce tumor microenvironment characteristic of pancreatic cancer, whereas DMEM contains glucose, amino acids, serum, and other nutrients that are essential for cell proliferation.

**Figure 2:**
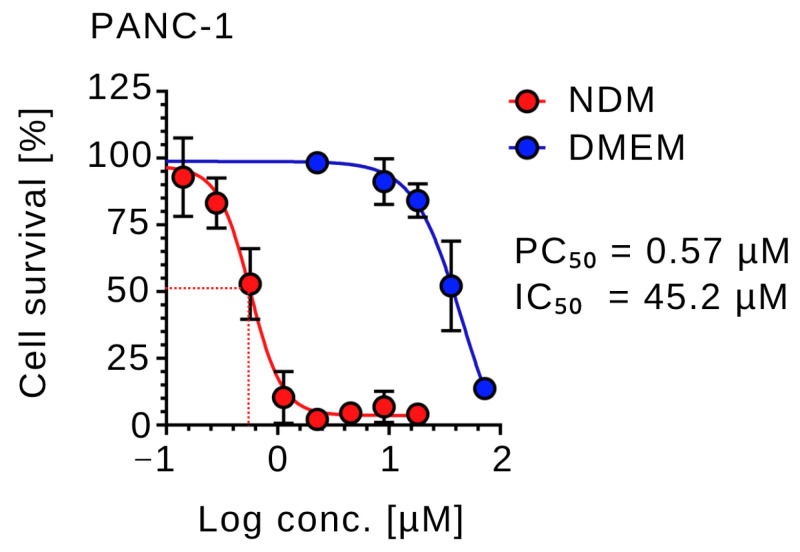
Preferential cytotoxicity of calliviminone A (CVM-A) against PANC-1 cells in nutrient-deprived medium (NDM) versus Dulbecco’s modified Eagle’s medium (DMEM). Data are presented as mean ± SD (*n* = 3).

Under nutrient-deprived conditions (NDM), CVM-A induced a pronounced dose-dependent reduction in the viability of PANC-1 cells. Concentrations as low as 0.1 µM significantly reduced cell survival, with a PC_50_ value of 0.57 µM (log concentration ≈ −0.25), corresponding to approximately 50% cell viability. At concentrations above this threshold, near-complete loss of cell viability was observed (Figure 2). Arctigenin, a positive control used in the study, showed PC_50_ value of 0.1 µM. These results demonstrated that CVM-A potently suppressed PANC-1 cell survival under nutrient stress, a condition in which these cells typically exhibit resistance to conventional chemotherapeutic agents. In contrast, in nutrient-rich DMEM, PANC-1 cells showed cytotoxicity only at elevated concentrations, with an IC_50_ value of 45.2 µM. Cell viability remained above 90% at concentrations below 25 µM, indicating minimal toxicity under nutrient-replete conditions. These results suggest the potential of CVM-A as a promising therapeutic agent for targeting the hypovascular pancreatic tumor microenvironment.

### 2.2. Real-Time Live-Cell Imaging of Calliviminone (CVM-A) A-Induced Morphological Changes and Cell Death in PANC-1 Pancreatic Cancer Cells in Nutrient-Deprived Medium (NDM)

**Figure 3:**
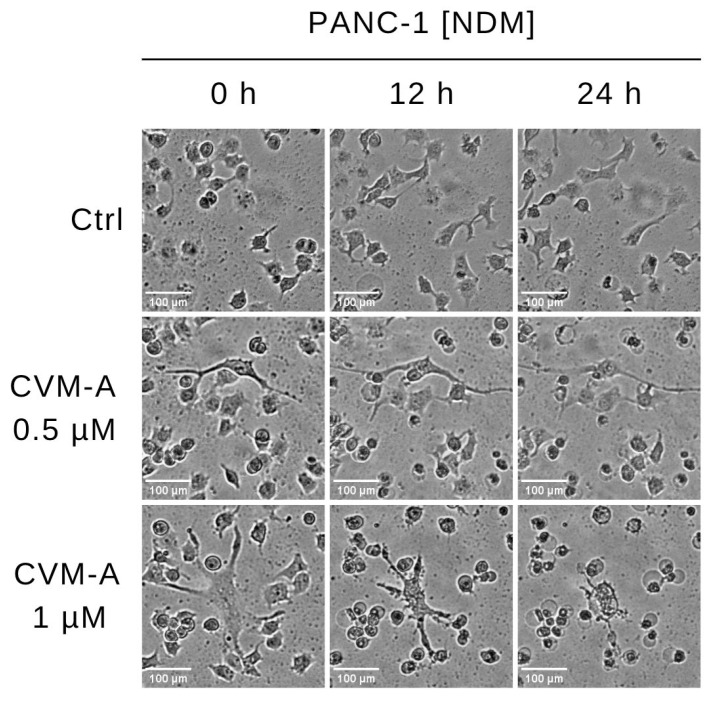
Time-lapse imaging of PANC-1 cells treated with calliviminone A (CVM-A, 0.5 and 1 µM) in NDM over 24 h, captured every 15 min using a CytoSMART digital microscopy system (see Supplementary Video S1).

To investigate the effects of CVM-A on cell morphology and viability, real-time live-cell imaging was performed on PANC-1 human pancreatic cancer cells cultured in nutrient-deprived medium (NDM). PANC-1 cells were treated with calliviminone A at concentrations of 0.5 µM and 1 µM. The control group contained untreated cells, incubated in a CO_2_ incubator equipped with a Cytosmart digital imaging system, and time-lapse images were captured every 15 min over 24 h. As shown in Figure 3, untreated PANC-1 cells maintained intact cellular morphology throughout the observation period, indicating survival under nutrient-deprived conditions. In contrast, cells treated with 1 µM CVM-A exhibited cell shrinkage and a rounded morphology within 12 h. After 24 h, pronounced morphological changes including membrane blebbing were observed. A detailed real-time visualization of the cell death process, demonstrating the anticancer potential of CVM-A, is provided in Supplementary Video S1.

### 2.3. Live-/Dead-Cell Imaging Analysis of Calliviminone A (CVM-A)-Induced Cytotoxicity in Nutrient-Deprived Medium (NDM)

**Figure 4:**
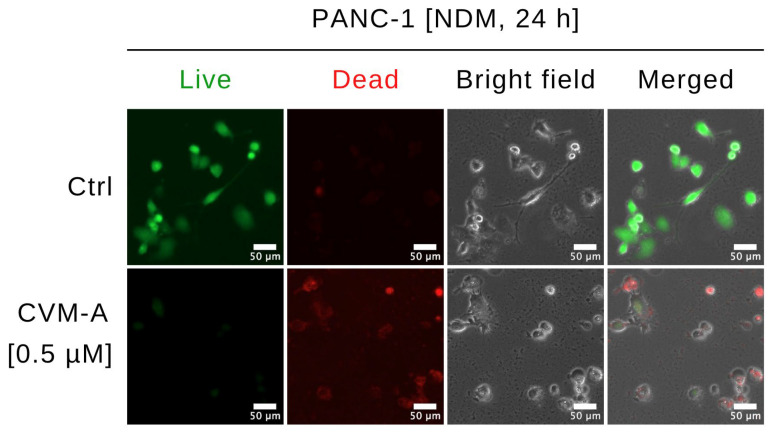
Morphological changes in PANC-1 cells treated with calliviminone A (CVM-A, 0.5 µM) in NDM for 24 h, stained with Live/Dead Cell Imaging Kit, and imaged using an EVOS FL digital microscope.

To investigate the effects of CVM-A on the morphology and viability of PANC-1, a Live/Dead Cell Imaging Kit was used. This two-color fluorescence assay distinguishes live and dead cells using distinct dyes: a live-cell component that emits intense, uniform green fluorescence in viable cells, and a dead-cell component that predominantly emits red fluorescence in cells with compromised membranes, indicative of cytotoxicity. PANC-1 cells were treated with 0.5 µM of CVM-A in nutrient-deprived medium (NDM) and compared with untreated controls. Imaging was performed using a fluorescence microscope system, as detailed in the Experimental section. As shown in Figure 4, untreated PANC-1 cells exhibited an intact morphology and emitted green fluorescence, indicative of viability. In contrast, treatment with 0.5 µM of CVM-A resulted in a marked increase in the number of cells emitting red fluorescence, which was accompanied by an altered morphology, including cell rounding and membrane disruption.

### 2.4. Real-Time Cell Migration Assay of Calliviminone A (CVM-A)-Induced Inhibition of PANC-1 Pancreatic Cancer Cell Migration in Normal Nutrient Medium (DMEM)

**Figure 5:**
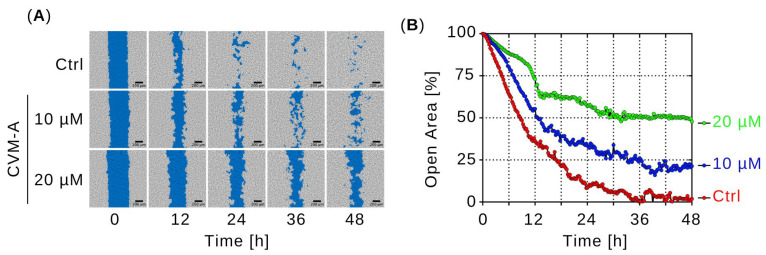
Effect of calliviminone A (CVM-A: 10 and 20 µM) on PANC-1 cell migration in DMEM over 48 h. (**A**) Real-time imaging of wound closure at 0, 12, 24, 36, and 48 h. (**B**) Quantification of open wound area (see Supplementary Video S2).

To evaluate the inhibitory effect of CVM-A on PANC-1 cell migration, real-time cell migration assay was performed. PANC-1 cells, either untreated (control) or treated with 10 or 20 µM of CVM-A in DMEM, were monitored for 48 h and images were captured every 15 min. Figure 5A shows representative images of the open areas of treated and untreated PANC-1 cells. In the control cells, the open area was nearly closed, reducing to 1.9% of the initial area after 48 h. In contrast, treatment with 10 µM and 20 µM of CVM-A resulted in open areas that remained at 21% and 47% of the initial area, respectively (Figure 5B). These findings demonstrated that calliviminone A significantly inhibited PANC-1 cell migration.

### 2.5. Calliviminone A (CVM-A)-Mediated Inhibition of PANC-1 Pancreatic Cancer Cell Colony Formation in Normal Nutrient Medium (DMEM)

**Figure 6:**
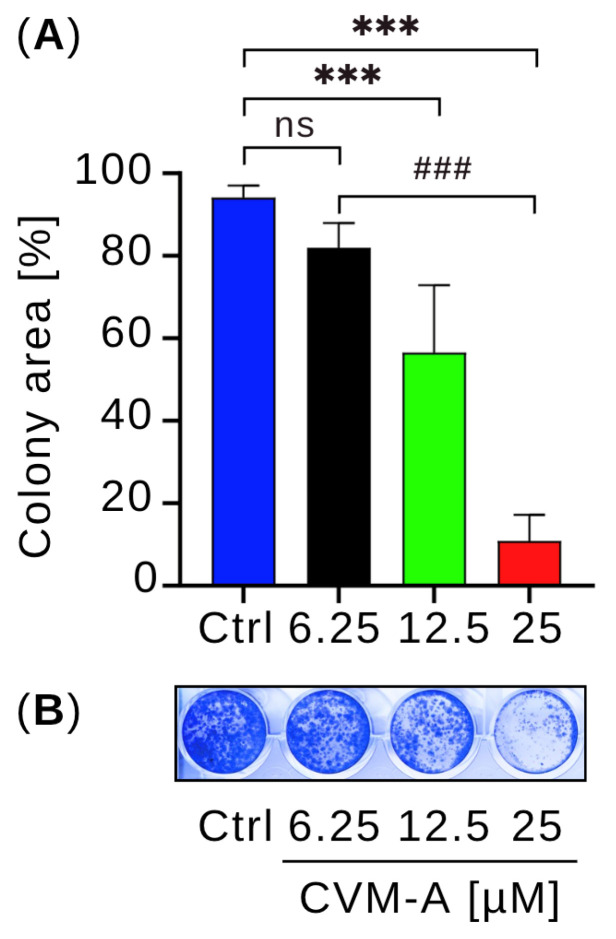
Effect of calliviminone A (CVM-A: 6.25, 12.5, 25 µM) on PANC-1 colony formation in DMEM after 10 days. (**A**) Mean colony area (*n* = 3). (**B**) Representative wells stained with crystal violet. ns = not significant, *** *p* < 0.001 vs. control, ^###^ *p* < 0.001 between treated groups.

To evaluate the ability of CVM-A to inhibit colony formation, we performed colony formation assay using PANC-1 human pancreatic cancer cells seeded in 24-well plates. The cells were treated with 6.25, 12.5, or 25 µM calliviminone A in DMEM for 24 h. The medium was then replaced with fresh DMEM, and the cells were cultured for an additional 10 d. Colonies were stained with crystal violet for visualization, and colony area was quantified using the Colony plugin in ImageJ software (version 1.54k). As shown in Figure 6, untreated PANC-1 control cells formed extensive colonies covering 94.2% of the well area. In contrast, treatment with CVM-A at 6.25 and 12.5 µM reduced colony formation, whereas at 25 µM, it suppressed colony formation by more than 80% in a dose-dependent manner compared with the control (*p* < 0.001). These results highlight the potent inhibitory effect of calliviminone A on pancreatic cancer cell colony formation.

### 2.6. Calliviminone A (CVM-A) Inhibits Insulin-Induced PI3K/Akt/mTOR Activation in PANC-1 Pancreatic Cancer Cells

**Figure 7:**
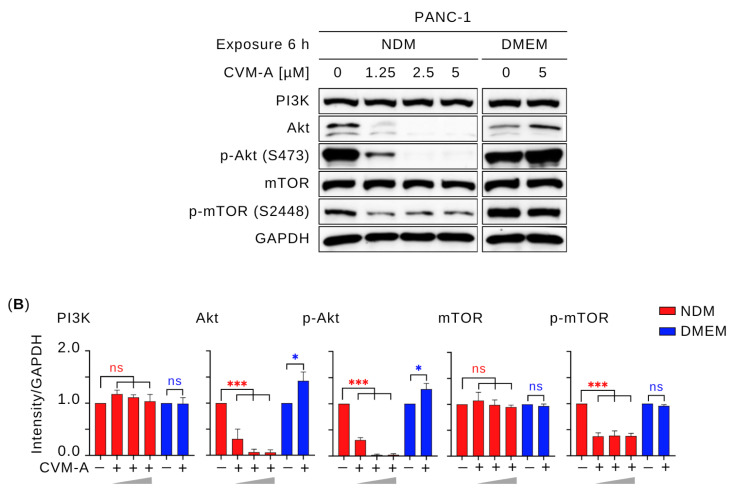
Effect of calliviminone A (CVM-A: 1.25, 2.5, 5 µM) on PI3K/Akt/mTOR pathway in PANC-1 cells. (**A**) Western blot. (**B**) Quantification of GAPDH-normalized band intensity (*n* = 3). ns = not significant, * *p* < 0.1, *** *p* < 0.001 vs. untreated groups. Red asterisks indicate comparisons to untreated control in NDM; blue asterisks indicate comparisons to untreated control in DMEM.

The PI3K/Akt/mTOR signaling pathway, a major downstream effector of the Ras family of proteins, regulates critical cellular processes including growth, survival, proliferation, metabolism, and motility [34,35]. To investigate whether CVM-A modulated this pathway, Western blotting was performed using PANC-1 human pancreatic cancer cells. Cells were treated with 1.25, 2.5, and 5 µM of CVM-A for 6 h in NDM and 5 µM in DMEM. As shown in Figure 7, CVM-A had minimal impact on Akt, phosphorylated Akt (p-Akt), and phosphorylated mTOR (p-mTOR) expression in PANC-1 cells cultured in nutrient-rich DMEM (*p* < 0.1, *p* < 0.1, and *p* < 0.0001, respectively). In contrast, in nutrient-deprived medium (NDM), CVM-A at 1.25 µM significantly reduced Akt and p-Akt levels (*p* < 0.001), with complete inhibition of both proteins at 2.5 and 5 µM (*p* < 0.001). Similarly, p-mTOR expression was significantly reduced in the NDM group at all the tested concentrations (*p* < 0.001). These results suggest that CVM-A disrupts the austerity response, impairing the cancer cells’ ability to survive nutrient stress.

In contrast, under nutrient-rich conditions (DMEM), CVM-A enhanced the expression of Akt and p-Akt, with relative expression levels increasing to 1.2–1.6 and 1.2–1.4, respectively, at 5 µM (Figure 7). This unexpected upregulation of p-Akt in DMEM might be transient, as the compound concurrently inhibits cancer cell migration and colony formation, suggesting it does not promote oncogenic behavior. The stable expression of PI3K in DMEM indicates that the enhancement of p-Akt may not be mediated by PI3K upregulation, raising the possibility that CVM-A inhibits negative regulators, such as PTEN, or activates growth factor receptor signaling, which requires further exploration.

**Figure 8:**
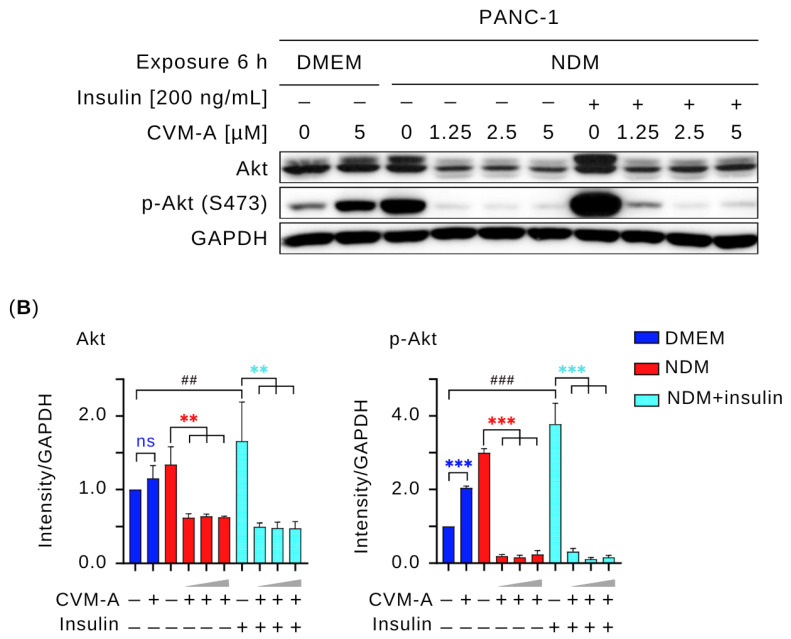
Effect of calliviminone A (CVM-A) with insulin (200 ng/mL) on Akt and p-Akt expression in PANC-1 cells under NDM. (**A**) Western blot analysis. (**B**) Quantification of GAPDH-normalized band intensity (*n* = 3). Statistical significance: ns = not significant, ** *p* < 0.01, *** *p* < 0.001 vs. untreated groups. Blue asterisks indicate comparisons to untreated control in DMEM; red asterisks indicate comparisons to untreated control in NDM, light blue asterisks indicate comparisons to untreated insulin-induced cells in NDM. ^##^ *p* < 0.01, ^###^ *p* < 0.001 vs. untreated control in DMEM.

Since CVM-A inhibited Akt signaling under NDM conditions, we next investigated whether it could also suppress growth factor-stimulated activation. Therefore, we performed a Western blot analysis to assess whether CVM-A downregulates insulin-induced Akt activation. For this purpose, PANC-1 cells were stimulated with insulin (200 ng/mL) alongside CVM-A treatment in NDM. As shown in Figure 8, insulin significantly upregulated Akt and p-Akt expression in NDM compared to untreated PANC-1 cells in DMEM (*p* < 0.01 and *p* < 0.001, respectively). However, co-treatment with CVM-A at 1.25 µM significantly reduced Akt expression (*p* < 0.01), whereas 2.5 and 5 µM CVM-A almost completely inhibited p-Akt in NDM (*p* < 0.001). These results indicate that insulin promotes Akt and p-Akt expression, whereas CVM-A potently downregulates these proteins, suggesting its potential as a therapeutic agent targeting the PI3K/Akt/mTOR pathway in the pancreatic cancer microenvironment.

## 3. Discussion

Human pancreatic cancer cells thrive in a hypovascular tumor microenvironment characterized by poorly organized blood vessels that severely restrict the availability of oxygen and nutrients [36,37]. Despite these harsh conditions, pancreatic cancer cells exhibit remarkable resilience to nutrient deprivation, enabling their survival in extreme microenvironments [2,38]. Targeting the ability to tolerate starvation is a promising strategy in drug discovery. Employing an anti-austerity approach, we identified calliviminone A (CVM-A) as a highly potent compound with preferential cytotoxicity against PANC-1 human pancreatic cancer cells, with a PC_50_ value of 0.57 µM. In addition to CVM-A, previous studies have identified structurally related compounds, including callistrilones L–N, which exhibit similar mechanisms of anti-austerity activity against pancreatic cancer cells. These compounds showed potent preferential cytotoxicity, with PC_50_ values of 0.065 µM, 0.038 µM, and 0.010 µM for callistrilones L, M, and N, respectively [29]. Structurally, CVM-A is classified as a phloroglucinol–meroterpenoid adduct, characterized by a phloroglucinol core conjugated to a meroterpenoid moiety [33]. In contrast, myrtucommulones and callistrilones L–N are distinct meroterpenoids existing as an equilibrium mixture of two conformational isomers [29,39]. These findings suggest that a phloroglucinol or related polyphenolic scaffold, combined with conjugated ketones, may be critical for selective cytotoxicity under nutrient-deprived conditions. Further investigation into the structural features responsible for their anti-austerity activity is warranted.

To explore the real-time effect of CVM-A on cell death, we performed real-time live-cell imaging and cell morphology analysis of PANC-1 cells under NDM. As shown in Figure 3, untreated PANC-1 cells maintained a healthy morphology, whereas cells treated with CVM-A exhibited significant shrinkage and a rounded morphology within 24 h (Supplementary Video S1). Furthermore, PANC-1 cells treated with 0.5 µM of CVM-A in NDM for 24 h were subjected to dual staining with Live/Dead Cell Imaging reagent, enabling the rapid assessment of cell viability based on plasma membrane integrity. As depicted in Figure 4, the control cells displayed an intact morphology and emitted green fluorescence, indicative of viability. In contrast, CVM-A treated cells exhibited rounded membranes, loss of integrity, and red fluorescence, confirming cell death. These findings prompted further investigation of the antimetastatic potential and molecular mechanisms underlying the anti-austerity activity of CVM-A.

Pancreatic cancer is often diagnosed at an advanced stage with metastatic spread, rendering curative surgery infeasible [3,40]. Patients with late-stage disease have a dismal prognosis, with a 5-year survival rate of 2.9% and a median survival of less than 6 months [41]. Agents that inhibit cancer cell migration can suppress tumor metastasis and improve survival outcomes. Therefore, we conducted a cell migration assay to evaluate the effects of CVM-A. Cell migration is a critical process in cancer progression [42,43]. In this study, an open area gap of PANC-1 cells was created using a two-well cell culture insert, allowing cells to migrate in the open area. Cell migration was compared with that of the control cells and cells treated with 10 and 20 µM of CVM-A. While control cells migrated rapidly to the open area and closed the wound area within 48 h, CVM-A significantly inhibited migration, reducing the open wound area by 47% at 20 µM compared with untreated controls (Figure 5 and Supplementary Video S2).

Metastatic pancreatic cancer cells often colonize secondary sites, such as the lungs and liver, forming small colonies that develop into large tumors—a process known as colonization [44,45]. The inhibition of colony formation can prevent metastatic progression. To test this hypothesis, we performed a colony formation assay. PANC-1 cells were seeded in 24-well plates at a density of 5000 cells/mL and treated with 0, 6.25, 12.5, or 25 µM calliviminone A for 24 h. After replacing the medium with fresh DMEM, cells were cultured for 10 days. As shown in Figure 6, untreated control cells formed colonies occupying 80% of the well area, whereas CVM-A significantly suppressed colony formation, particularly at 12.5 and 25 µM concentrations, where colony formation was markedly reduced. These results suggested that CVM-A is a promising lead compound for the development of antimetastatic therapies.

The PI3K/Akt/mTOR signaling pathway is a critical regulator of cancer hallmarks, including cell survival, proliferation, metabolism, and metastasis, and is frequently activated in pancreatic cancer [46,47,48]. Akt phosphorylation (p-Akt) has been reported to be an austerity marker that is expressed by pancreatic cancer cells during nutrient starvation, leading these cells to tolerate and survive. In addition, p-Akt at Thr308 and Ser473, triggered by insulin receptor activation and PI3K signaling, produces the active p-Akt form, which mediates glucose uptake and cell survival [49,50,51]. To investigate the effects of CVM-A on this pathway, Western blotting was performed to assess the expression levels of key survival and proliferation proteins. As shown in Figure 7, CVM-A significantly downregulated Akt and p-Akt expression, achieving complete inhibition at 2.5 µM in NDM. Similarly, mTOR phosphorylation is markedly reduced under these conditions. Notably, insulin-induced Akt and p-Akt expression was suppressed by CVM-A at 1.25, 2.5, and 5 µM concentrations. These findings indicate that CVM-A inhibits the critical components of the PI3K/Akt/mTOR pathway under tumor microenvironment mimicking condition of nutrition deprivation, reinforcing its potential as a candidate for pancreatic cancer drug development.

## 4. Materials and Methods

### 4.1. Reagents

Dulbecco’s Modified Eagle’s medium (DMEM; high-glucose and glucose-free), D-PBS (−) powder, penicillin–streptomycin–amphotericin B suspension, and trypsin were purchased from Wako Pure Chemical Industries (Osaka, Japan). Fetal bovine serum (FBS) was obtained from Nichirei Biosciences, Inc. (Tokyo, Japan). Calliviminone A (CVM-A) was isolated from *Callistemon citrinus* leaves, and its purity (>95%) was confirmed by ^1^H and ^13^C NMR spectroscopy using a JNM-ECS400 spectrometer (JEOL Ltd., Tokyo, Japan) (see Supporting Information for details on isolation and purification). Arctigenin (purity > 95%) was purchased form Tokyo Chemical Industry Co., Ltd. (TCI, Tokyo, Japan). The Cell Counting Kit-8 was purchased from Dojindo Laboratories (Kumamoto, Japan). Rabbit polyclonal antibodies against PI3K, Akt, phosphorylated Akt (Ser473), mTOR, phosphorylated mTOR (S2448), and GAPDH were obtained from Cell Signaling Technology (Danvers, MA, USA). Horseradish peroxidase (HRP)-conjugated goat polyclonal anti-rabbit IgG was purchased from DakoCytomation (Glostrup, Denmark). Resolving gel buffer, stacking gel buffer, and 30% acrylamide/bis solution were procured from Bio-Rad Laboratories Inc. (Hercules, CA, USA).

### 4.2. Cell Culture

The human pancreatic cancer cell line, PANC-1 (RBRC-RCB2095), was obtained from the RIKEN BioResource Center (BRC) Cell Bank. The cells were cultured in DMEM supplemented with 10% FBS, 0.1% sodium bicarbonate, and 1% penicillin–streptomycin. All the cells were maintained in a humidified incubator at 37 °C and 5% CO_2_ throughout the experiments.

### 4.3. Preferential Cytotoxicity Assay

PANC-1 cells (2 × 10^4^ cells/well) were seeded in a 96-well plate and allowed to adhere overnight. Cells were then treated with CVM-A under two different conditions: nutrient-rich medium (DMEM) and nutrient-deprived medium (NDM) for 24 h. CVM-A was dissolved in dimethyl sulfoxide (DMSO) at 10 mM and diluted in NDM or DMEM to the desired concentrations, with a final DMSO concentration of ≤1% (*v*/*v*). Control treatments with ≤1% DMSO alone showed no significant effect on PANC-1 cell viability (>95%), confirming that the observed activity was due to CVM-A. The NDM was prepared using glucose-free Dulbecco’s Modified Eagle’s Medium (DMEM) from Wako Pure Chemical Industries (Osaka, Japan) supplemented only with 1% penicillin–streptomycin–amphotericin B suspension. Following treatment, NDM was replaced with fresh DMEM containing 5% Cell Counting Kit-8 solution and incubated for 3 h. Absorbance was measured at 450 nm wavelength using a microplate reader (MULTISKAN SkyHigh, Thermo Scientific, Waltham, MA, USA). Cell viability was calculated based on the following equation using the mean values from triplicate wells and was analyzed using GraphPad Prism (version 10.5.0) [31].


Cell survival (%) = [(Abs_(samples)_ − Abs_(blank)_)/(Abs_(control)_ − Abs_(blank)_)] × 100

For subsequent assays, CVM-A was used at concentrations of 0.5–5 µM in NDM (based on its PC_50_ = 0.57 µM) to evaluate anti-austerity activity. Higher concentrations (6.25–25 µM) were applied in DMEM to assess anti-metastatic and anti-proliferative effects under non-cytotoxic conditions (below IC_50_ = 50 µM), ensuring assay specificity.

### 4.4. Live-Cell Imaging

PANC-1 cells (2 × 10^5^ cells/well) were seeded into 35 mm dishes and incubated overnight for adhesion. The cells were treated with calliviminone A at concentrations of 0.5 and 1 µM in NDM. Live-cell imaging was performed in a CO_2_ incubator equipped with the CytoSMART digital microscopy system. Time-lapse images were captured at 15 min intervals for 24 h and analyzed using ImageJ software [32].

### 4.5. Morphological Changes

PANC-1 cells (2 × 10^5^ cells/well) were plated in 35 mm dishes and allowed to adhere overnight. Cells were treated the next day with 0.5 µM calliviminone A in NDM for 24 h. Following treatment, cells were stained with 10 µL of the Live/Dead Cell Imaging Kit (Invitrogen, Thermo Fisher Scientific, Waltham, MA, USA) for 15 min. Images were captured using an EVOS FL digital microscope (Thermo Fisher Scientific, Waltham, MA, USA) in fluorescence (red and green) and phase-contrast modes using a 20× objective lens [39].

### 4.6. Cell Migration Assay

PANC-1 cells (1 × 10^6^ cells/mL) were seeded in two-well culture inserts (70 µL/well) to create a uniform wound gap. Cells were treated with 10 and 20 µM of CVM-A in DMEM and monitored using the CytoSMART system for 48 h. Real-time images were acquired at 15 min intervals. Wound closure areas were quantified using Fiji software (version 1.54k) and analyzed using GraphPad Prism software [31].

### 4.7. Colony Formation Assay

PANC-1 cells (5 × 10^3^ cells/well) were seeded in 24-well plates in DMEM and treated with 6.25, 12.5, and 25 µM of CVM-A for 24 h. The medium was then replaced with fresh DMEM and the cells were incubated for 10 days to allow colony formation. Colonies were stained with crystal violet solution for 15 min and the Colony Area was quantified using the “Colony Area” plugin in ImageJ [39].

### 4.8. Western Blot Analysis

PANC-1 cells (1 × 10^6^ cells/well) were seeded in six-well plates and treated with insulin (200 ng/mL), followed by CVM-A at 0, 1.25, 2.5, and 5 µM in NDM and 0 and 5 µM in DMEM for 6 h. After treatment, the cells were lysed using RIPA buffer supplemented with sodium orthovanadate, protease inhibitor cocktail, β-glycerophosphate disodium salt hydrate, and phenylmethanesulfonyl fluoride. Proteins were separated via SDS-PAGE and transferred to polyvinylidene fluoride (PVDF) membranes. The membranes were blocked with 5% (*w*/*v*) skim milk in TBST, washed, and incubated overnight with primary antibodies against PI3K, Akt, p-Akt (S473), mTOR, p-mTOR (S2448), and GAPDH. After washing, the membranes were incubated with an HRP-conjugated goat anti-rabbit secondary antibody for 1 h at room temperature. Detection was performed using enhanced chemiluminescence (Bio-Rad, Hercules, CA, USA), and band intensities were quantified using the Fiji software [39].

### 4.9. Statistical Analysis

Statistical analyses were performed using GraphPad Prism 8.4.3. Data are reported as means ± standard deviation (SD). Unpaired Student’s *t*-tests were used for comparisons between the non-treated control and treated groups in experiments conducted in Dulbecco’s Modified Eagle’s Medium (DMEM). For multiple comparisons, one-way ANOVA followed by Dunnett’s test was used. Statistical significance was set at *p* < 0.05.

## 5. Conclusions

Calliviminone A (CVM-A), isolated from *Callistemon citrinus* leaves, demonstrated potent anti-austerity activity against PANC-1 pancreatic cancer cells in nutrient-deprived medium (NDM), with a PC_50_ of 0.57 µM. At concentrations of 0.5–5 µM in NDM, CVM-A effectively inhibited cell survival by suppressing phosphorylation of Akt and mTOR—key mediators of cancer cell adaptation to metabolic stress. Additionally, in nutrient-rich conditions (DMEM), CVM-A inhibited cell migration and colony formation at non-cytotoxic concentrations (6.25–25 µM). These results highlight CVM-A’s potential as a promising lead compound for anti-austerity-based therapy targeting the unique metabolic vulnerabilities of pancreatic cancer.

## Data Availability

All data supporting the reported results, including NMR spectra, replicate data for three independent experiments, and real-time cell migration movies, are provided in the Supporting Information.

## Supplementary Materials

The following supporting information can be downloaded at https://www.mdpi.com/article/10.3390/plants14132074/s1, Plant material, extraction and isolation, Table S1:^1^H and ^13^C NMR spectroscopic data of calliviminone A (CVM-A); Figure S1:^1^H NMR spectrum of CVM-A; Figure S2:^13^C NMR spectrum of CVM-A; Figure S3: Real-time live-cell imaging of CVM-A-induced morphological changes and cell death in PANC-1 pancreatic cancer cells; Figure S4: Real-time cell migration assay of CVM-A -induced inhibition of PANC-1 pancreatic cancer cell migration; Figure S5: Three independent experiments of CVM-A-mediated modulation of the PI3K/Akt/mTOR signaling pathway in PANC-1 pancreatic cancer cells in NDM and DMEM; Figure S6: Three independent experiments of CVM-A on insulin-induced Akt activation in NDM; Video S1: Real-time live-cell imaging of CVM-A induced morphological changes and cell death in PANC-1 pancreatic cancer cells; Video S2: Real-time cell migration assay of CVM-A-induced inhibition of PANC-1 pancreatic cancer cell migration.
