# Supplementary material for: Calliviminone A from Callistemon citrinus Induces PANC-1 Pancreatic Cancer Cell Death by Targeting the PI3K/Akt/mTOR Pathway
Source: Plants (Basel). 2025 Jul 7;14(13):2074. doi: 10.3390/plants14132074 (PMC12252234; doi:10.3390/plants14132074)
Supplement: Supplementary file 1 [file plants-14-02074-s001.zip › Supplementary Material.pdf]

Supplementary Material

Calliviminone A from *Callistemon citrinus* Induces PANC-1 Pancreatic Cancer Cell Death by Targeting the PI3K/Akt/mTOR Pathway

Juthamart Maneenet [1], Ahmed M. Tawila [1], Hung Hong Nguyen [1], Nguyen Duy Phan [1], Orawan Monthakantirat [2], Supawadee Daodee [2], Chantana Boonyarat [2], Charinya Khamphukdee [3], YaowaredChulikhit [2] and Suresh Awale [1,*]

[1] Natural Drug Discovery Laboratory, Institute of Natural Medicine, University of Toyama, 2630 Sugitani, Toyama 930-0194, Japan.

[2] Division of Pharmaceutical Chemistry, Faculty of Pharmaceutical Sciences, Khon Kaen University, Khon Kaen 40002, Thailand.

[3] Division of Pharmacognosy and Toxicology, Faculty of Pharmaceutical Sciences, Khon Kaen University, Khon Kaen 40002, Thailand.

* Correspondence: suresh@inm.u-toyama.ac.jp; Tel./Fax: +81-76-434-7640

Table of Contents

Plant material.. 3

Extraction and isolation.. 3

Table S1. ^{1}H and ^{13}C NMR spectroscopic data of calliviminone A (CVM-A).. 5

Figure S1. ^{1}H NMR spectrum of calliviminone A (CVM-A).. 6

Figure S2. ^{13}C NMR spectrum of calliviminone A (CVM-A).. 7

Figure S3. Real-time live cell imaging of calliviminone A (CVM-A)-induced morphological changes and cell death in PANC-1 pancreatic cancer cells.. 8

Figure S4. Real-time cell migration assay of calliviminone A (CVM-A)-induced inhibition of PANC-1 pancreatic cancer cell migration.. 33

Figure S5. Three independent experiments of calliviminone A (CVM-A)-mediated modulation of the PI3K/Akt/mTOR signaling pathway in PANC-1 pancreatic cancer cells in NDM and DMEM....... 82

Figure S6. Three independent experiments of calliviminone A (CVM-A) on insulin-induced Akt activation in NDM.. 83

Plant Material

The *Callistemon citrinus* leaves were collected during August 2017 from El-Zohreya Botanical Garden, Giza, Egypt. This plant was authenticated by senior botanists, Mrs. Therese Labib, Consultant of Plant Taxonomy at the Ministry of Agriculture and El-Orman Botanical Garden, Giza, Egypt. A voucher specimen (000077CC@05-01-04-01) was deposited at the El-Orman Garden's herbarium. The *C. citrinus* leaves were dried, ground, and kept in a tight container throughout the experiment.

Extraction and Isolation

The dried *C. citrinus* leaves (290 g) were extracted with CH_2Cl_2 using the sonication extraction method (1.5 L, 90 min × 3). The CH_2Cl_2 solvent was filtered and concentrated by using a rotary evaporator to yield a CH_2Cl_2 extract (28 g). The *C. citrinus* CH_2Cl_2 extract was then chromatographed on silica gel by MPLC using *n*-hexane/ ethyl acetate (EtOAc) gradient mixture (0–100%) as the mobile phase to give four fractions (Fr. 1, 5.2 g; Fr. 2, 2.5 g; Fr. 3, 10.0 g; Fr. 4, 7.3 g). Fraction 1 (5.2 g) was chromatographed on normal-phase silica gel MPLC using *n*-hexane/ CH_2Cl_2 solvent mixtures (0–100%) to afford five subfractions (Fr. 1-1, 604 mg; Fr. 1-2, 939 mg; Fr. 1-3, 197 mg; Fr. 1-4, 750 mg; Fr. 1-5, 1.5 g). Subfraction 1-3 (197 mg) was purified on silica gel MPLC column using *n*-hexane/ EtOAc to afford two fractions (Fr. 1-3-1, 81 mg; Fr. 1-3-2, 101 mg). Subfraction 1-3-2 (101 mg) was purified by semi-preparative HPLC on an ODS column (30 × 250 mm, 5 μm, Tosoh) using an eluent system consisting of acetonitrile containing 0.1% formic acid to obtain calistrilone L (**1**, 2.2 mg). Subfraction 1-4 (750 mg) was subjected to passage over silica gel MPLC column and eluted with *n*-hexane/ EtOAc, providing six subfractions (Fr. 1-4-1, 6.0 mg: Fr. 1-4-2, 36.0 mg; Fr. 1-4-3, 58.0 mg; Fr. 1-4-4, 112 mg; Fr. 1-4-5, 232 mg; Fr. 1-4-6, 202 mg). Subfraction 1-4-2 (36 mg) was purified by preparative TLC using *n*-hexane/ CH_2Cl_2 (2:3) to give calliviminone A (**13**, 3.1 mg). Subfraction 1-4-3 (58 mg) was subjected to preparative HPLC on an ODS column (30 × 250 mm, 5 μm, Tosoh) using MeOH containing 0.03% formic acid as a mobile phase, to yield seven compounds, calistrilone M (**2**, 1.9 mg), callistrilone N (**3**, 3.7 mg), callistrilone O (**4**, 2.7 mg), callistrilone P (**5**, 1.7 mg), callistrilone Q (**6**, 1.1 mg), epicallistrilone Q (**7**, 1.3 mg), and callistrilone E (**8**, 1.6 mg). Subfraction 1-4-5 (232 mg) was subjected to preparative HPLC on an ODS column (30 × 250 mm, 5 μm, Tosoh) using an eluent system consisting of acetonitrile containing 0.1% formic acid, to give six compounds, callistiviminene N (**9**, 6.6 mg), callistibiminene M (**10**, 10.5 mg), callistiviminene F (**11**, 5.2 mg), myrtucommulone L (**12**, 8.0 mg), myrtucommulone K (**14**, 1.4 mg), and callistiviminene I (**15**, 13.1 mg). Fraction 3 (6.0 g) was chromatographed by normal-phase silica gel MPLC using *n*-hexane/ CH_2Cl_2–0.1% MeOH as solvent system with a gradual increase of CH_2Cl_2, to afford five subtractions (Fr. 3-1, 112 mg; Fr. 3-2, 3.5 g; Fr. 3-3, 314 mg; Fr. 3-4, 516 mg; Fr. 3-5, 213 mg). Subfraction 3-1

(112 mg) was rechromatographed by normal-phase silica gel MPLC using a gradient solvent mixture of n-hexane/ acetone to yield two compounds, endoperoxide G3 (**17**, 7.0 mg) and isoguaiacin (**21**, 2.3 mg). Subfraction 3-2 (3.5 g) was subjected to reverse-phase silica gel column chromatography using an acetonitrile/ water gradient system (50-100%) as mobile phase, to give six compounds, 8-demethyl eucalypin (**18**, 11.0 mg), eucalyptin (**19**, 16.0. mg), 3β-hydroxy-urs-11-en-13(28)-olide (**22**, 6.0 mg), betulin (**25**, 16.0 mg), uvaol (**23**, 7.0 mg), and erythrodiol (**28**, 3.5 mg). Subfraction 3-4 (516 mg) was rechromatographed over a reverse-phase silica gel column using an acetonitrile/ water gradient system (50-100%) as mobile phase to give three subfractions (Fr. 3-4-1, 11.1 mg; Fr. 3-4-2, 297 mg; Fr. 3-4-3, 167 mg). Subfraction 3-4-1 was identified as platanic acid (**26**, 11.1 mg). Subfraction 3-4-3 (167 mg) was purified further over a silica gel column using *n*-hexane/ acetone gradient system, to afford two compounds, callistenone D (**16**, 3.1 mg) and olenolic acid (**24**, 6.0 mg). Fraction 4 (7.3 g) was subjected to normal-phase silica gel MPLC column using CH_2Cl_2/ MeOH as mobile phase to give four subfracitions (Fr. 4-1, 3.1 g; Fr. 4-2, 3.4 g; Fr. 4-3, 300 mg; Fr. 4-4, 259 mg). Subfraction 4-3 was identified as catechin (**20**, 200 mg). Subfraction 4-2 (3.4 g) was purified over normal-phase silica gel MPLC using CH_2Cl_2/ MeOH gradient mixture to afford two compounds, betulinic acid (**27**, 56.0 mg) and ursolic acid (**29**, 37.0 mg).

Table S1. ^{1}H and ^{13}C NMR spectroscopic data of calliviminone A (CVM-A) in $CDCl_3$ (δ in ppm, J in Hz).

no.	CVM-A	
	δ_H	δ_C
1	-	208.69
2	-	56.49
3	-	213.04
4	-	56.78
5	-	208.79
6	-	67.09
7	2.19, m[a]	47.28
8	1.64, m[a]	30.14
9	0.88, d (6.71)	24.20
10	0.83, d (6.71)	19.13
11	1.40, s	26.32
12	1.37, s	24.94
13	1.38, s	25.07
14	1.33, s	26.05
1′α	2.47, dd (16.48)	31.14
1′β	2.13, m[a]	-
2′	5.25, br s	115.46
3′	-	138.11
4′α	2.22, m[a]	27.77
4′β	2.05, m[a]	-
5′	1.95, m[a]	37.17
6′	2.07, m[a]	26.26
7′	5.04, t	124.16
8′	-	131.61
9′	1.66, s	25.80
10′	1.59, s	17.82

[a] Signal pattern unclear due to overlapping.

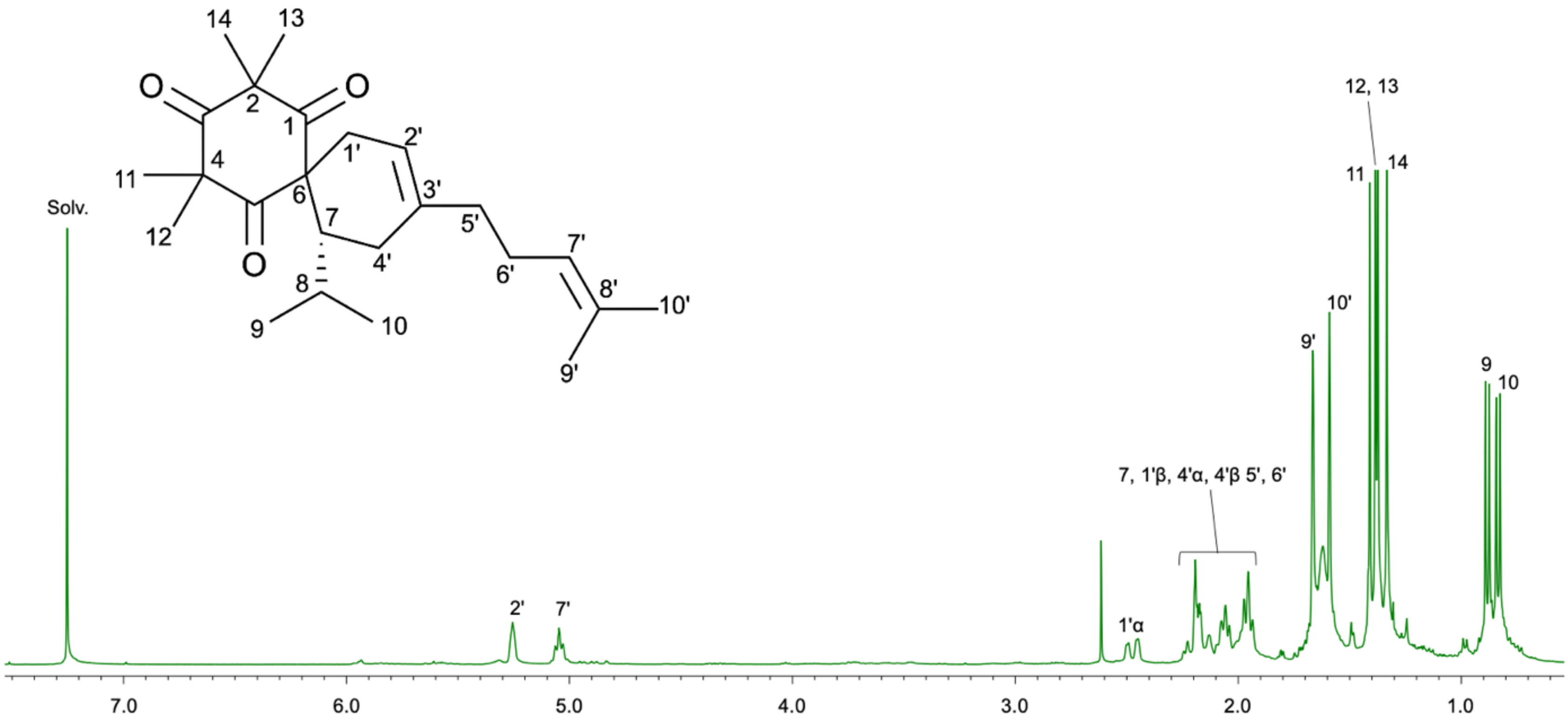

Figure S1. ^{1}H NMR spectrum of CVM-A (400 MHz, $CDCl_3$).

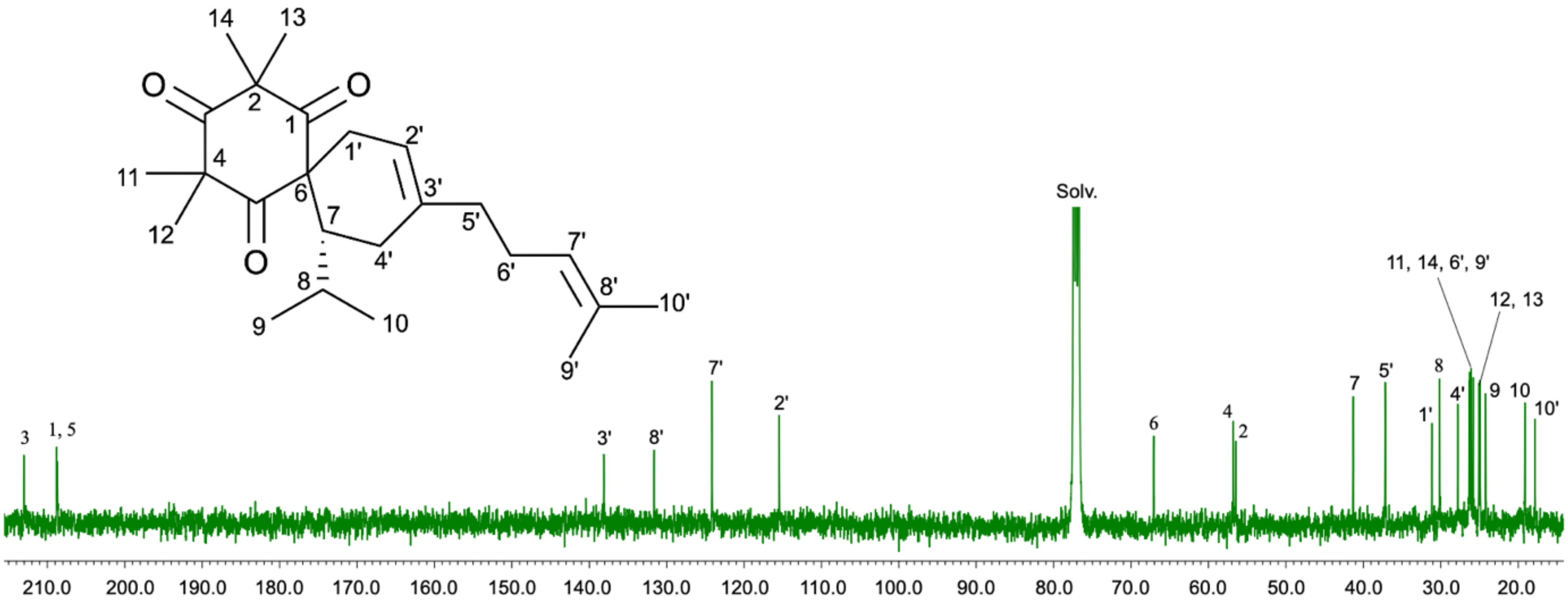

Figure S2. ^{13}C NMR spectrum of CVM-A (100 MHz, $CDCl_3$)

Figure S3. Real-time live cell imaging of calliviminone A (CVM-A)-induced morphological changes and cell death in PANC-1 pancreatic cancer cells.

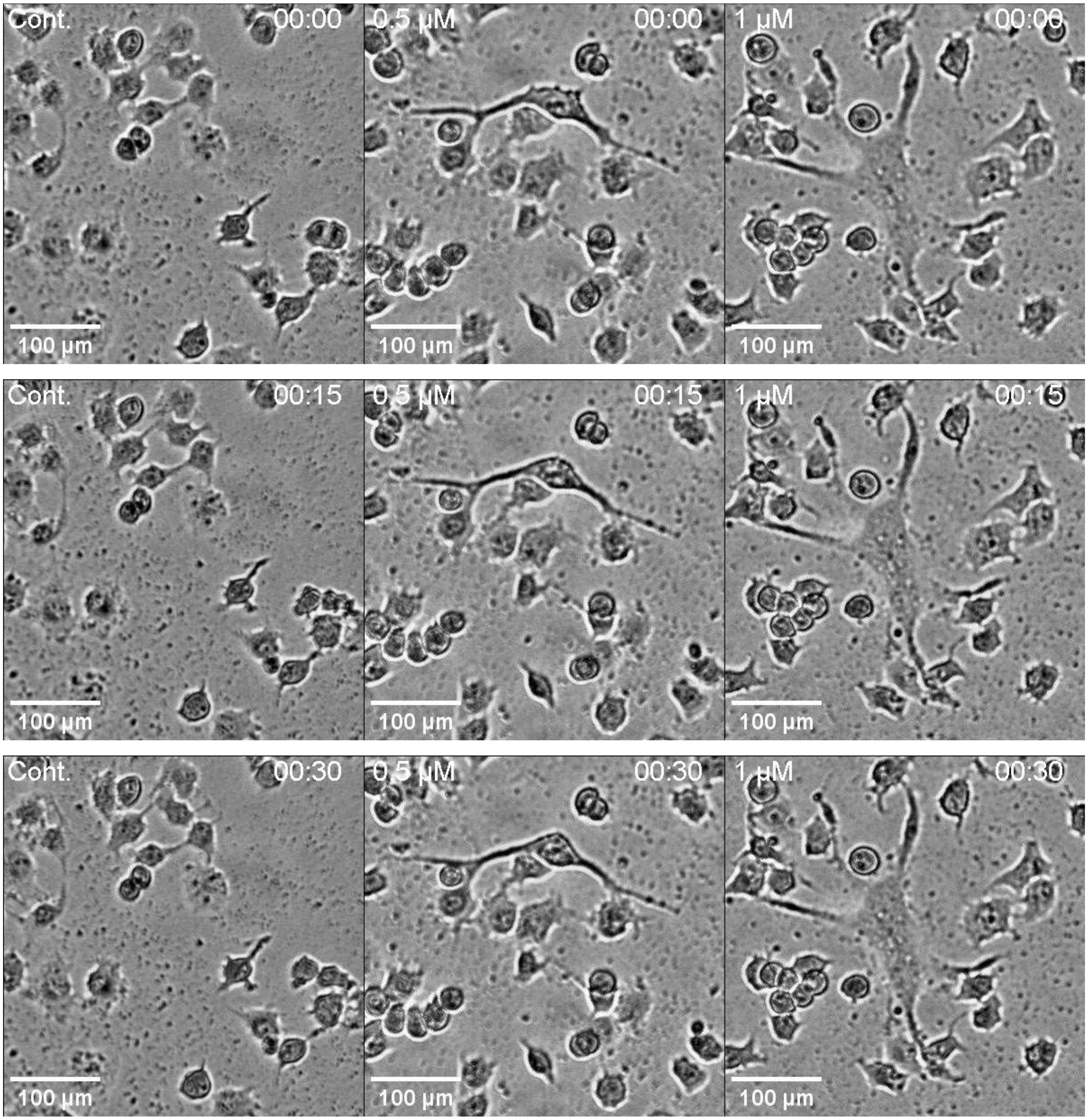

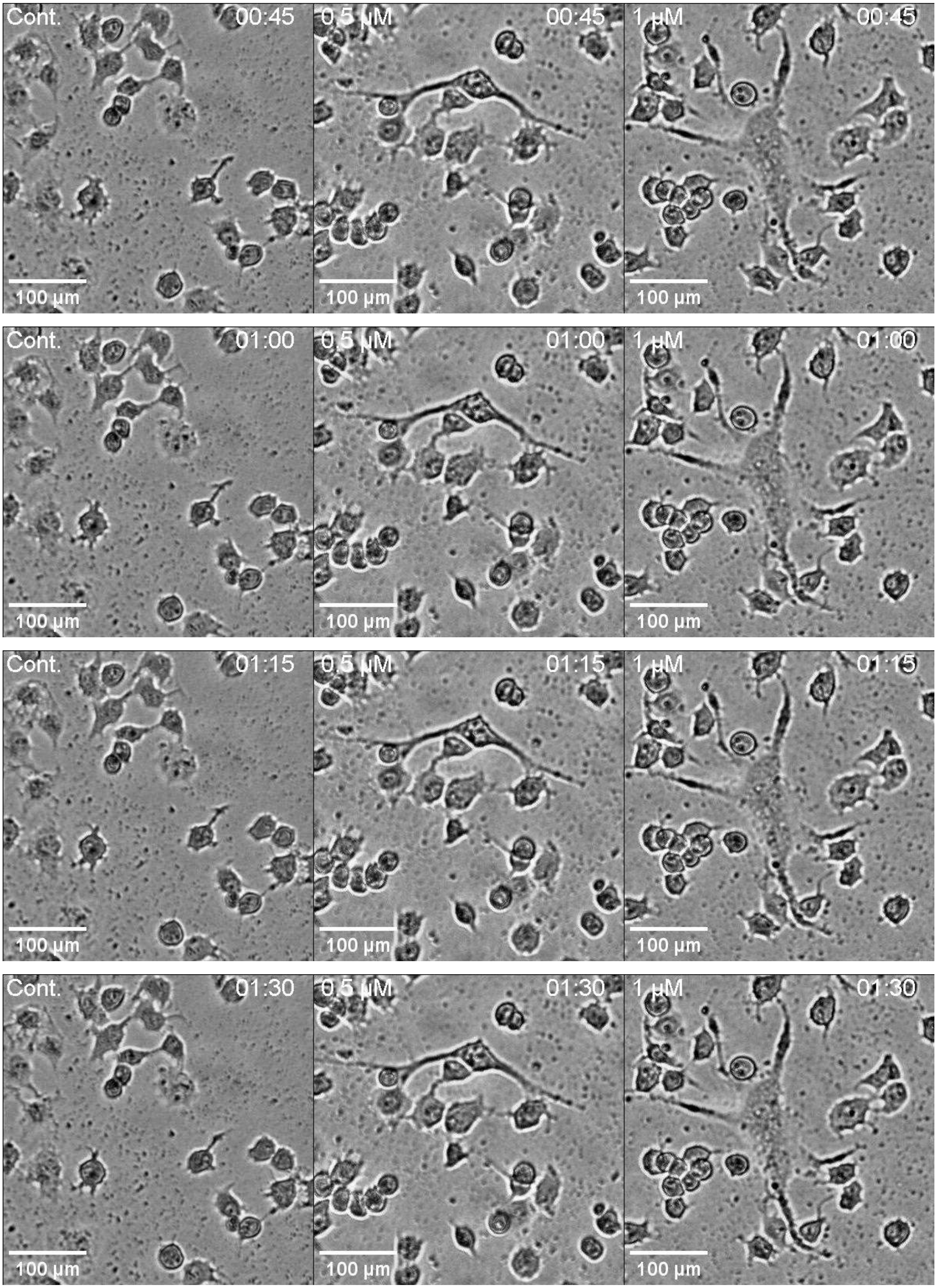
Cont.
00:45
0.5 μM
00:45
1 μM
00:45
100 μm
Cont.
01:00
0.5 μM
01:00
1 μM
01:00
100 μm
Cont.
01:15
0.5 μM
01:15
1 μM
01:15
100 μm
Cont.
01:30
0.5 μM
01:30
1 μM
01:30
100 μm

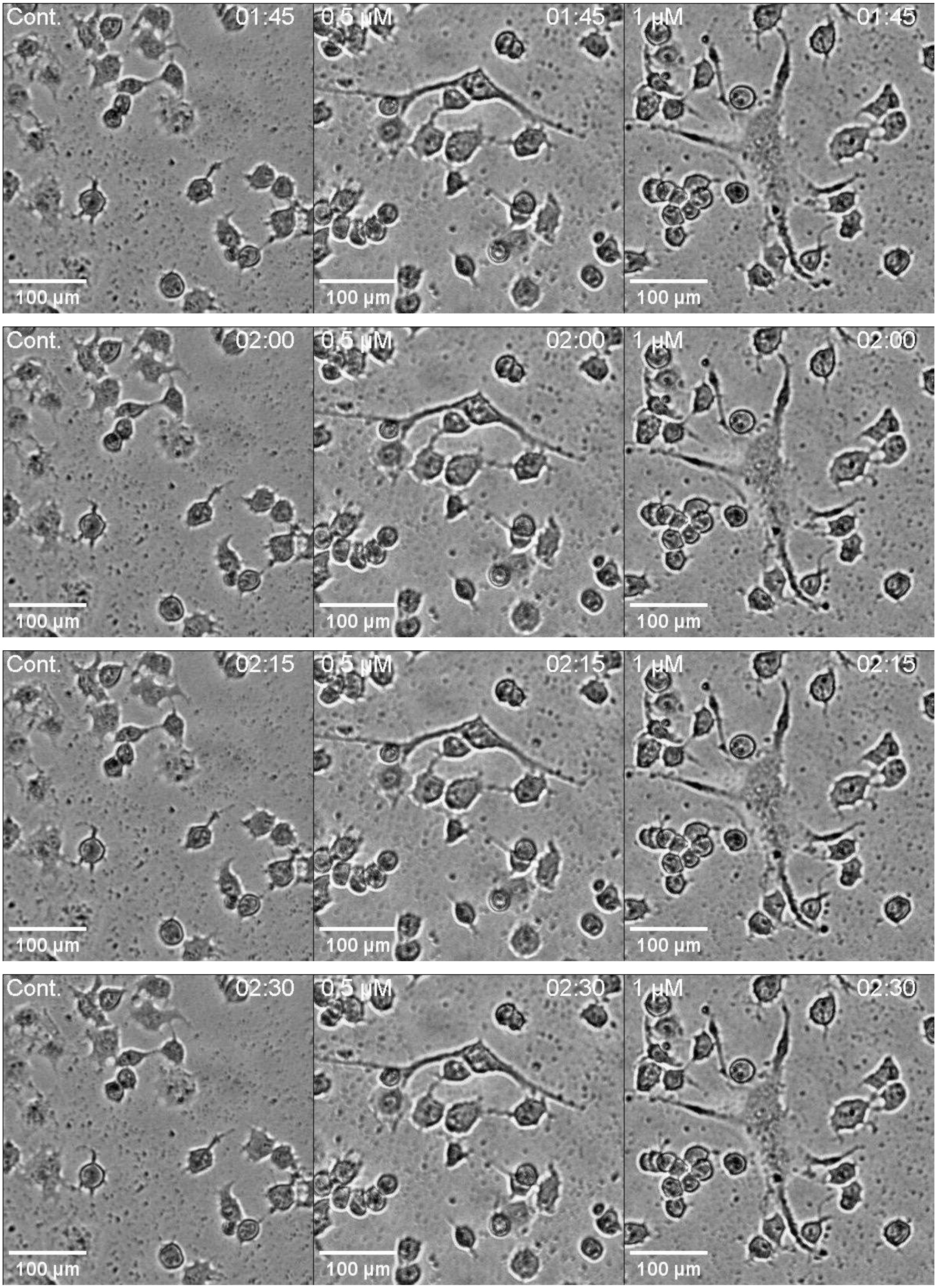
Cont.
01:45
0.5 μM
01:45
1 μM
01:45
100 μm
02:00
02:15
02:30

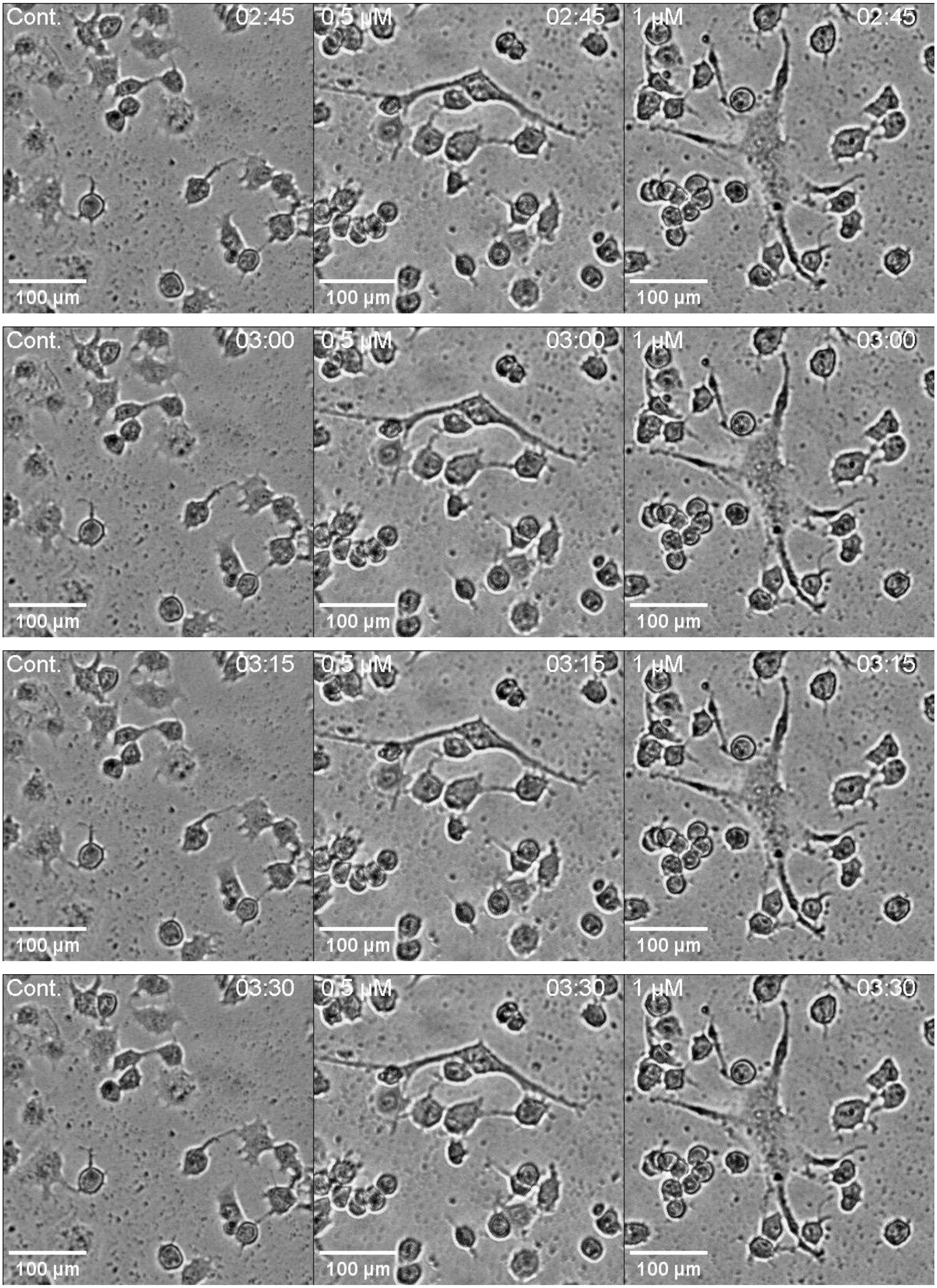
Cont.
02:45
0.5 μM
02:45
1 μM
02:45
100 μm
Cont.
03:00
0.5 μM
03:00
1 μM
03:00
100 μm
Cont.
03:15
0.5 μM
03:15
1 μM
03:15
100 μm
Cont.
03:30
0.5 μM
03:30
1 μM
03:30
100 μm

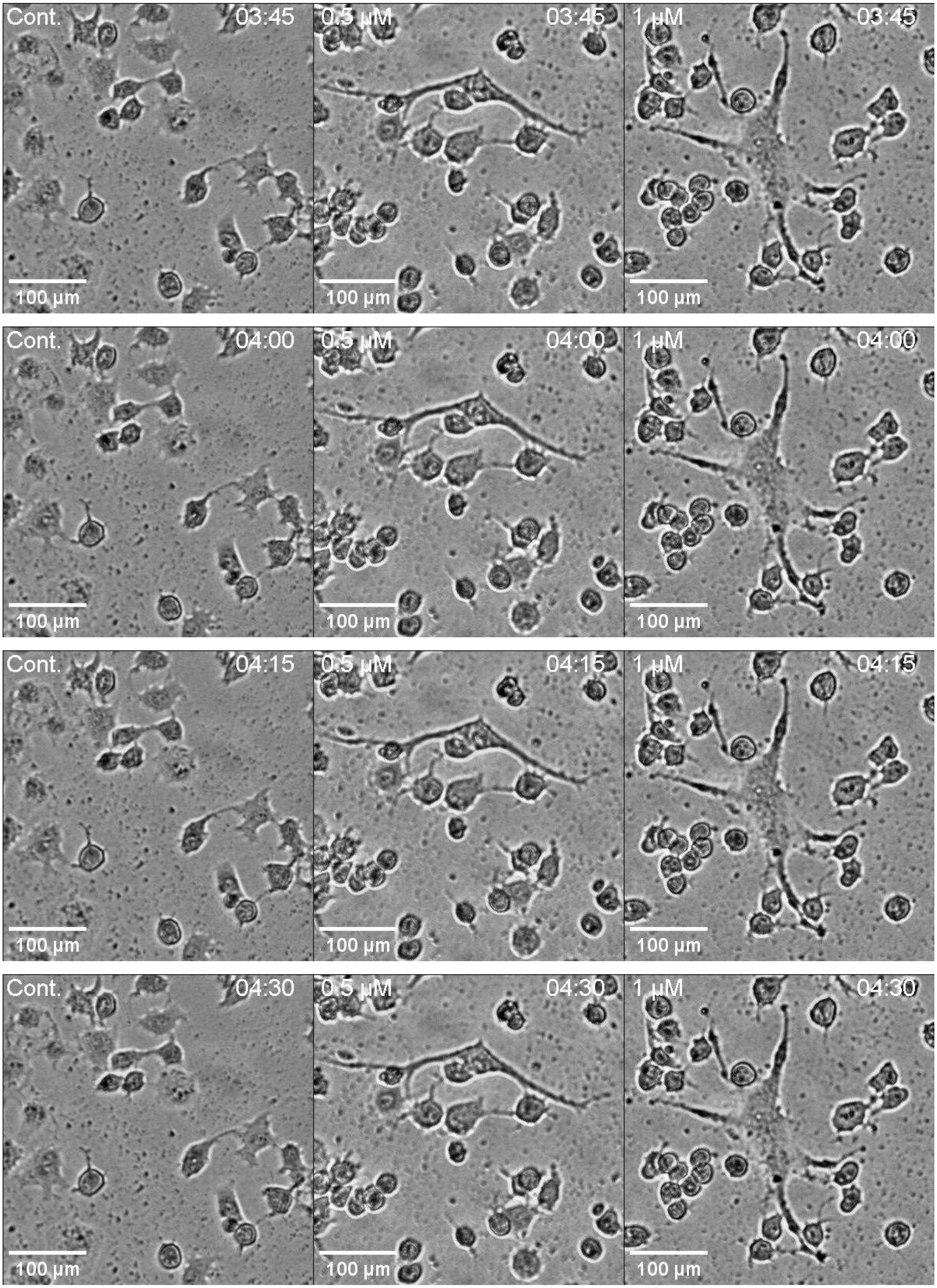

Cont.
03:45
100 µm
0.5 µM
03:45
100 µm
1 µM
03:45
100 µm
Cont.
04:00
100 µm
0.5 µM
04:00
100 µm
1 µM
04:00
100 µm
Cont.
04:15
100 µm
0.5 µM
04:15
100 µm
1 µM
04:15
100 µm
Cont.
04:30
100 µm
0.5 µM
04:30
100 µm
1 µM
04:30
100 µm

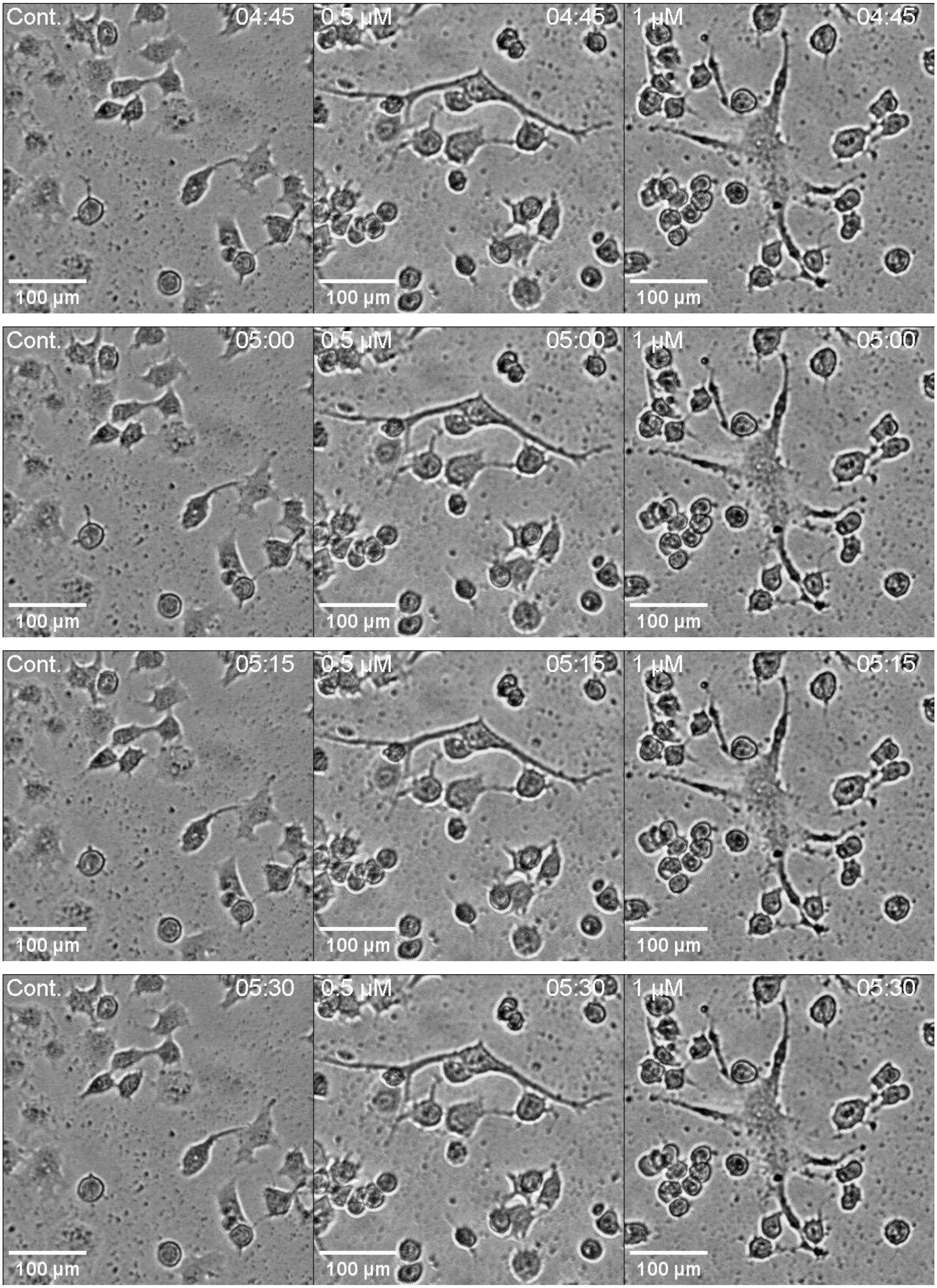
Cont.
04:45
0.5 µM
04:45
1 µM
04:45
100 µm
Cont.
05:00
0.5 µM
05:00
1 µM
05:00
100 µm
Cont.
05:15
0.5 µM
05:15
1 µM
05:15
100 µm
Cont.
05:30
0.5 µM
05:30
1 µM
05:30
100 µm

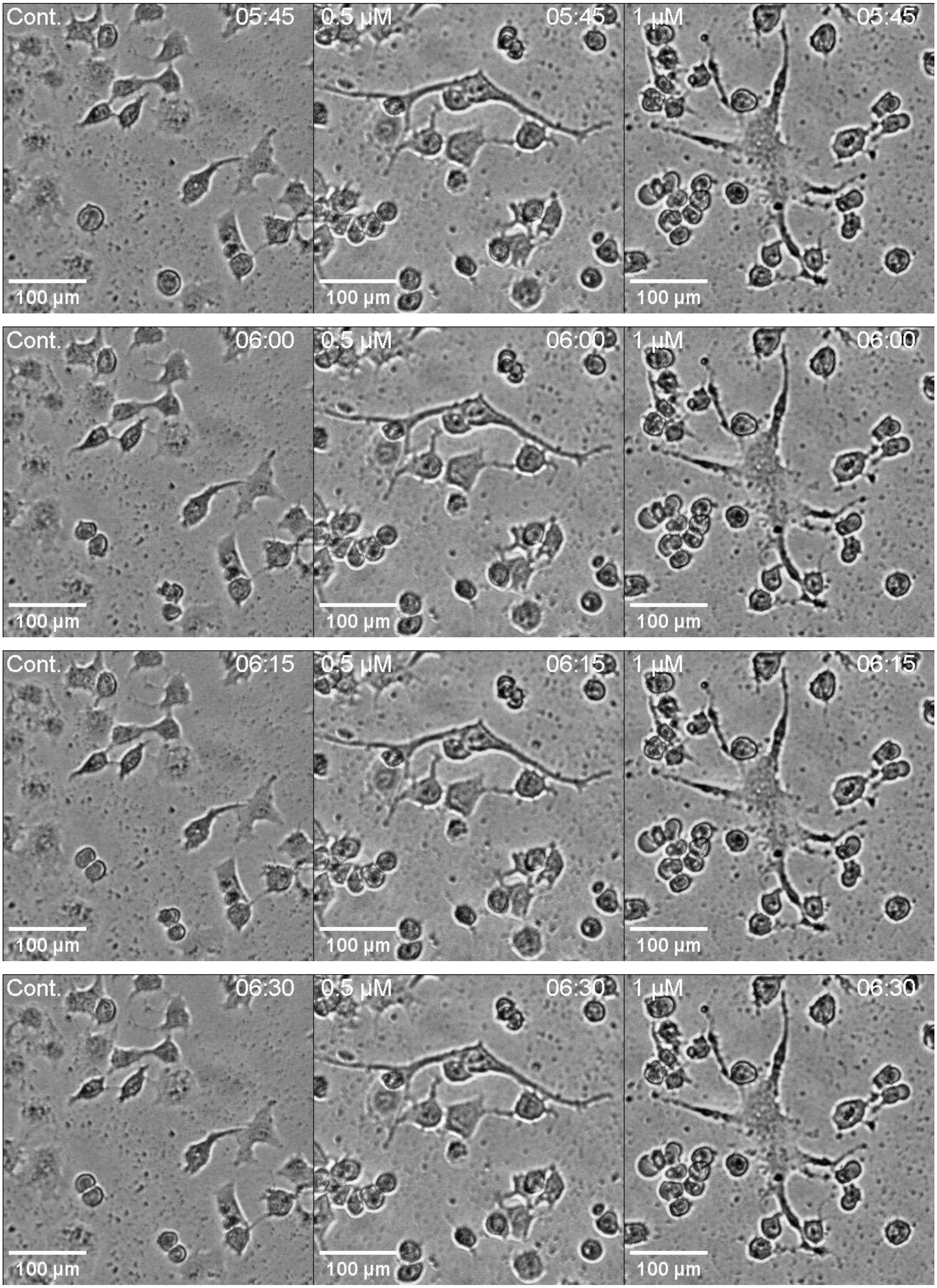
Cont.
05:45
0.5 µM
05:45
1 µM
05:45
Cont.
06:00
0.5 µM
06:00
1 µM
06:00
Cont.
06:15
0.5 µM
06:15
1 µM
06:15
Cont.
06:30
0.5 µM
06:30
1 µM
06:30
100 µm

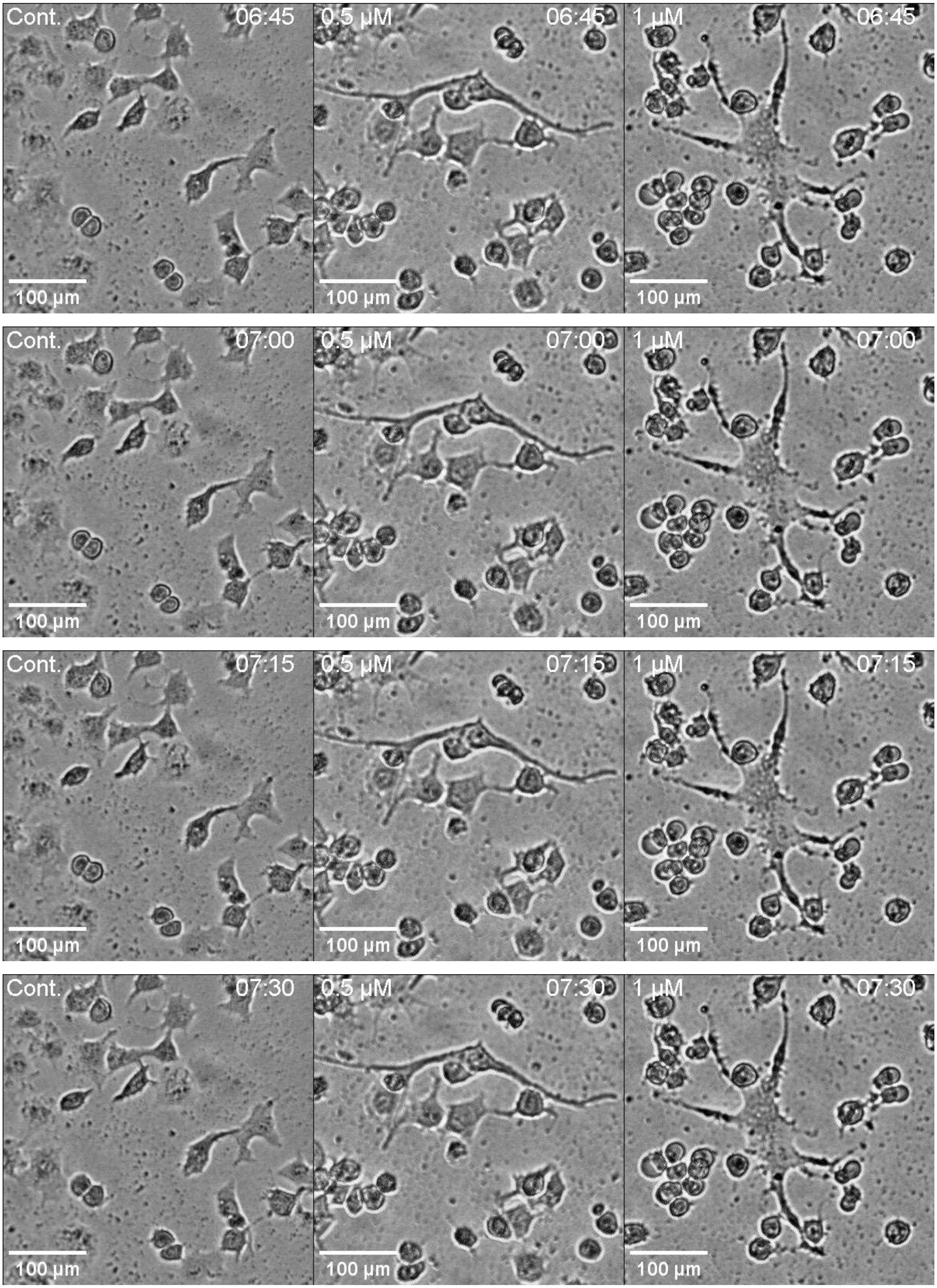
Cont.
06:45
0.5 μM
06:45
1 μM
06:45
Cont.
07:00
0.5 μM
07:00
1 μM
07:00
Cont.
07:15
0.5 μM
07:15
1 μM
07:15
Cont.
07:30
0.5 μM
07:30
1 μM
07:30
100 μm

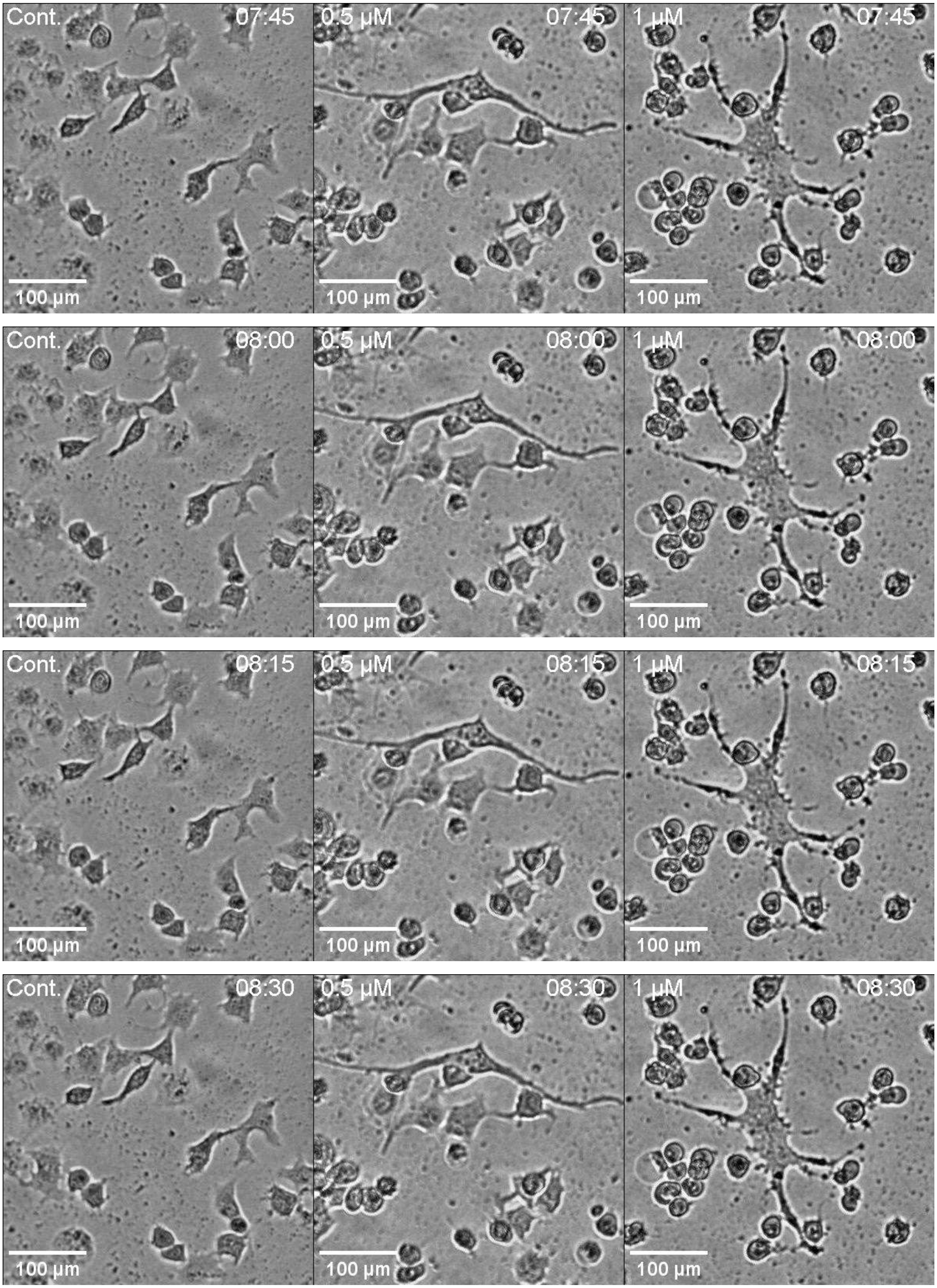
Cont.
07:45
0.5 μM
07:45
1 μM
07:45
08:00
08:15
08:30
100 μm

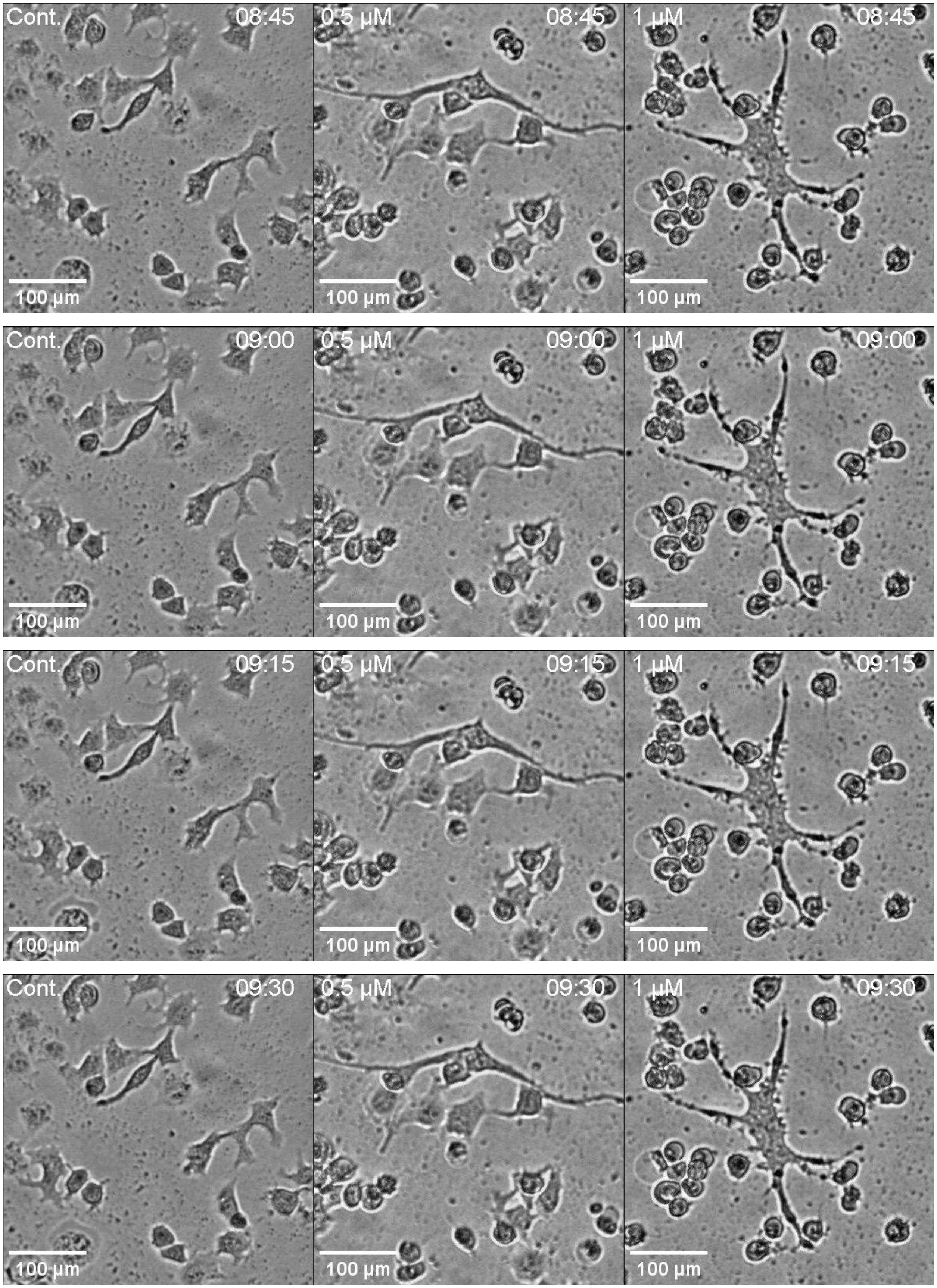
Cont.
08:45
0.5 μM
08:45
1 μM
08:45
09:00
09:15
09:30
100 μm

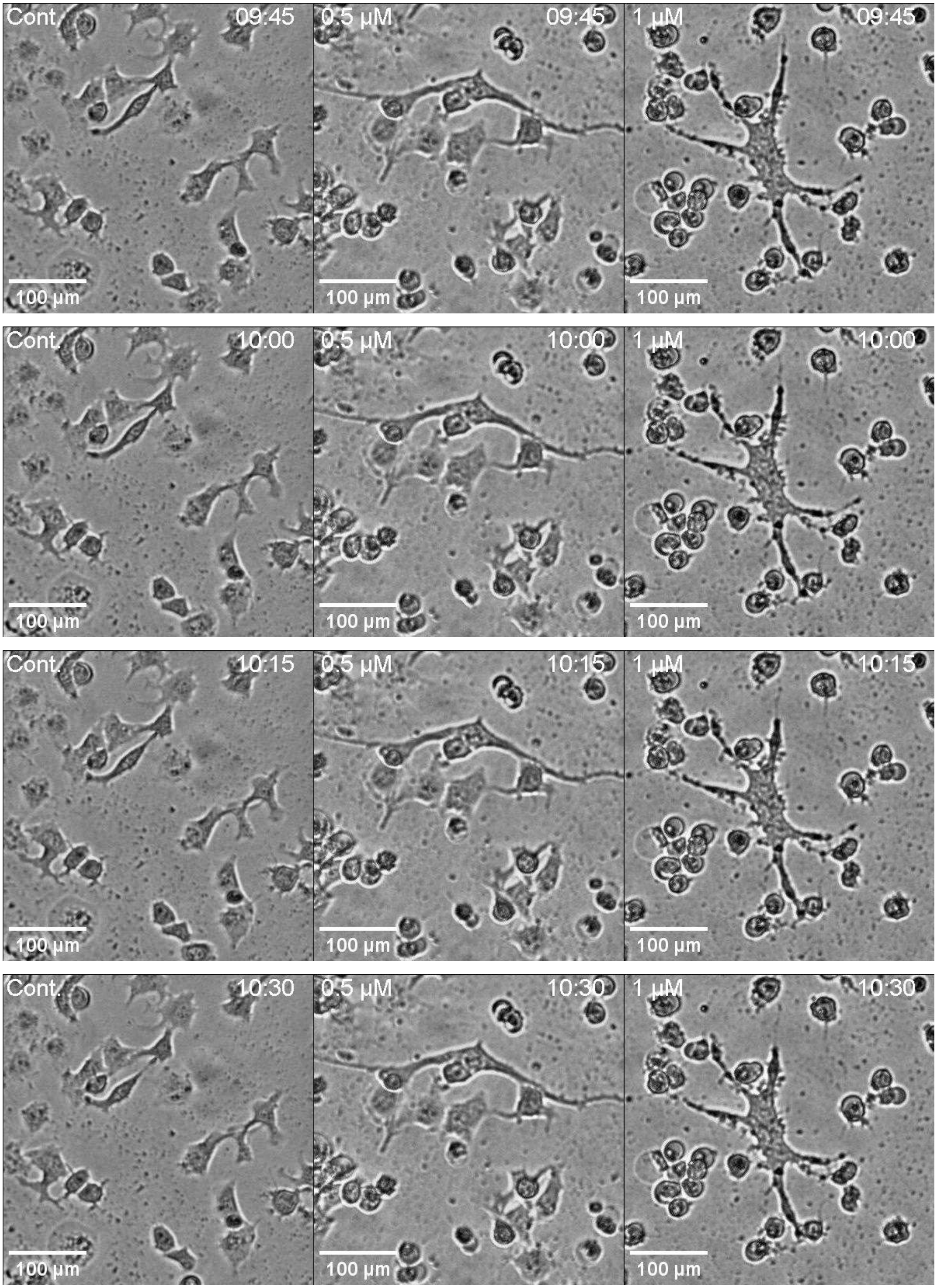
Cont.
09:45
0.5 µM
09:45
1 µM
09:45
100 µm
100 µm
100 µm
Cont.
10:00
0.5 µM
10:00
1 µM
10:00
100 µm
100 µm
100 µm
Cont.
10:15
0.5 µM
10:15
1 µM
10:15
100 µm
100 µm
100 µm
Cont.
10:30
0.5 µM
10:30
1 µM
10:30
100 µm
100 µm
100 µm

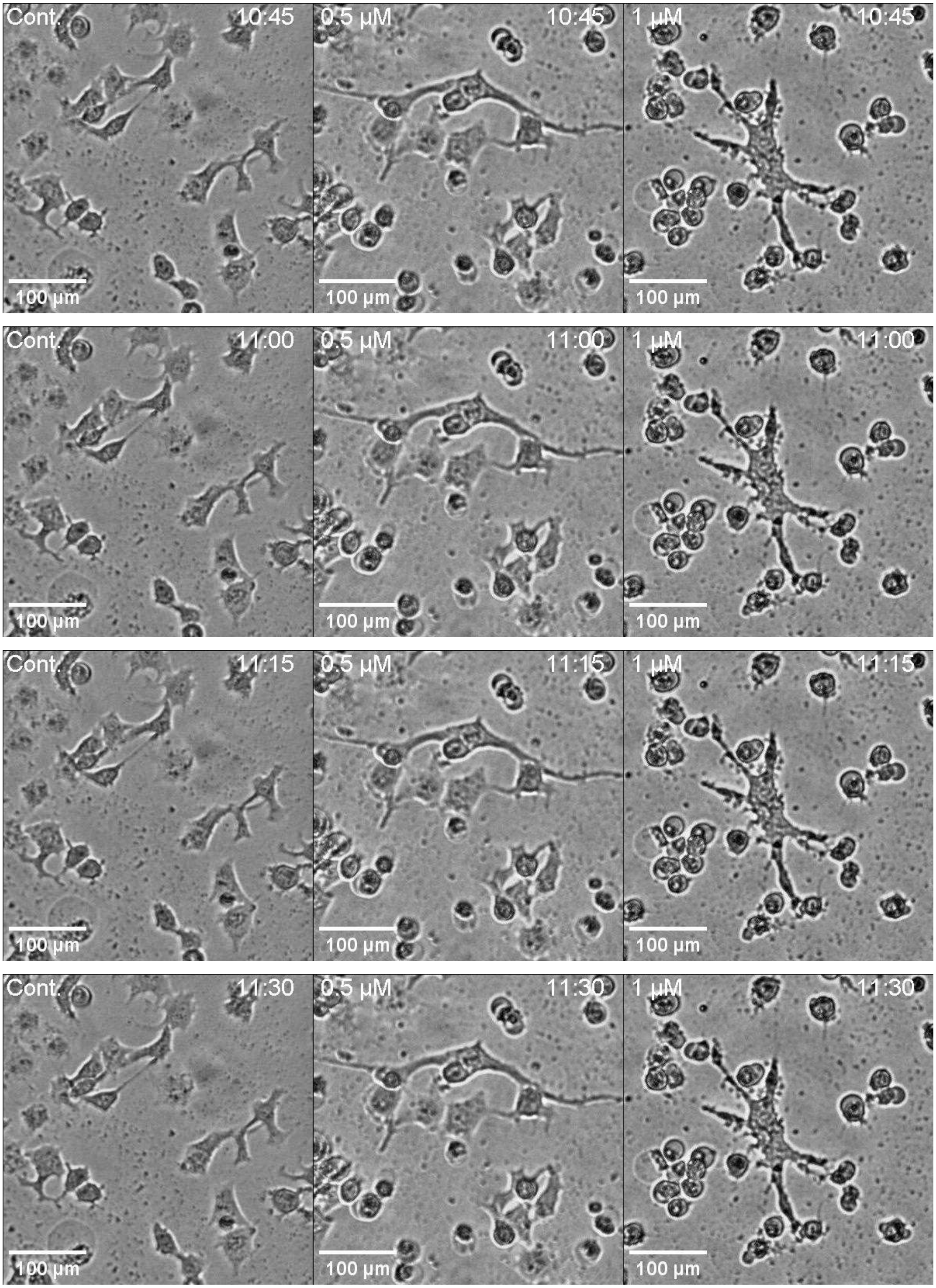
Cont.
10:45
100 µm
0.5 µM
10:45
100 µm
1 µM
10:45
100 µm
Cont.
11:00
100 µm
0.5 µM
11:00
100 µm
1 µM
11:00
100 µm
Cont.
11:15
100 µm
0.5 µM
11:15
100 µm
1 µM
11:15
100 µm
Cont.
11:30
100 µm
0.5 µM
11:30
100 µm
1 µM
11:30
100 µm

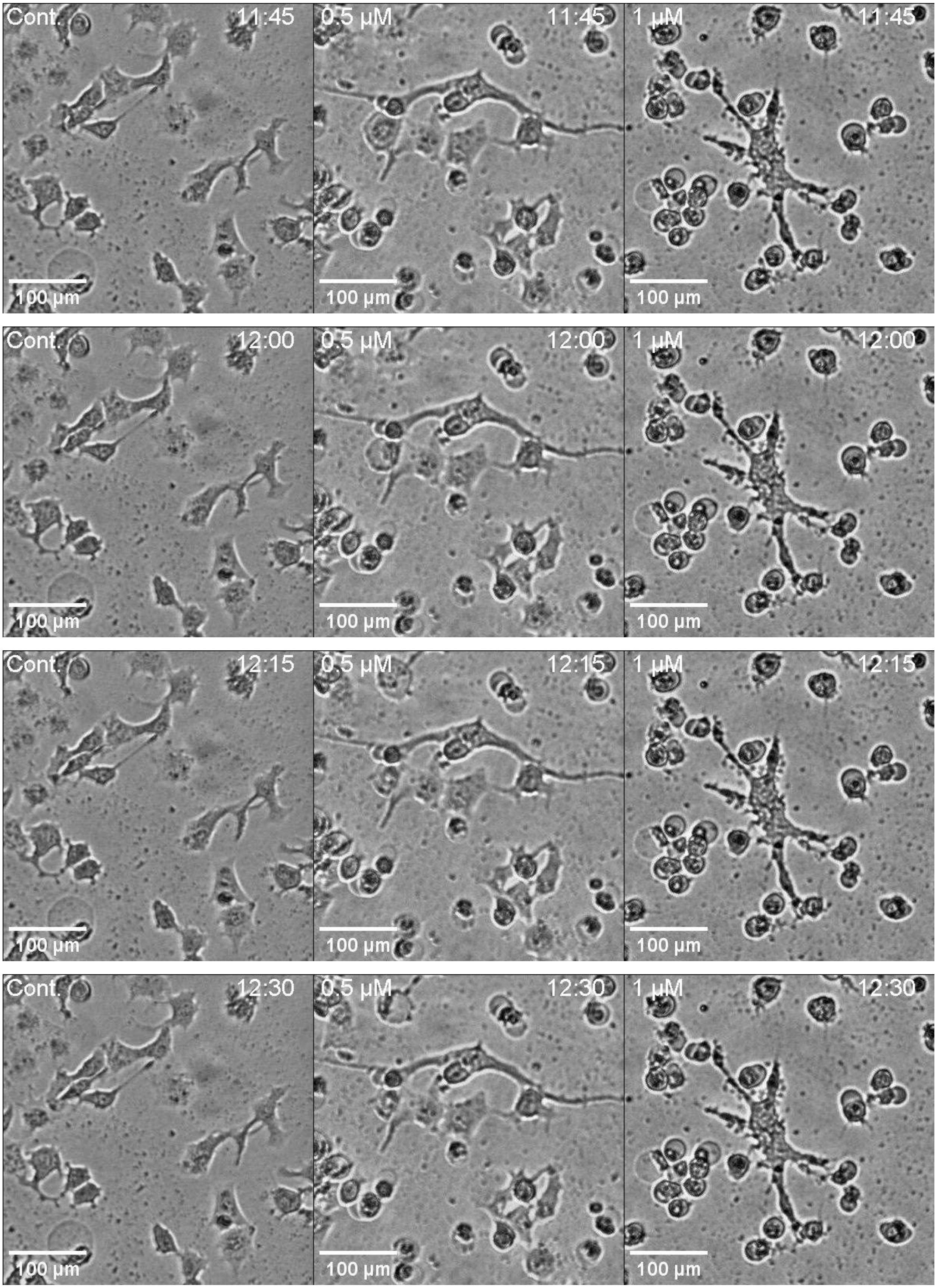
Cont.
11:45
100 µm
0.5 µM
11:45
100 µm
1 µM
11:45
100 µm
Cont.
12:00
100 µm
0.5 µM
12:00
100 µm
1 µM
12:00
100 µm
Cont.
12:15
100 µm
0.5 µM
12:15
100 µm
1 µM
12:15
100 µm
Cont.
12:30
100 µm
0.5 µM
12:30
100 µm
1 µM
12:30
100 µm

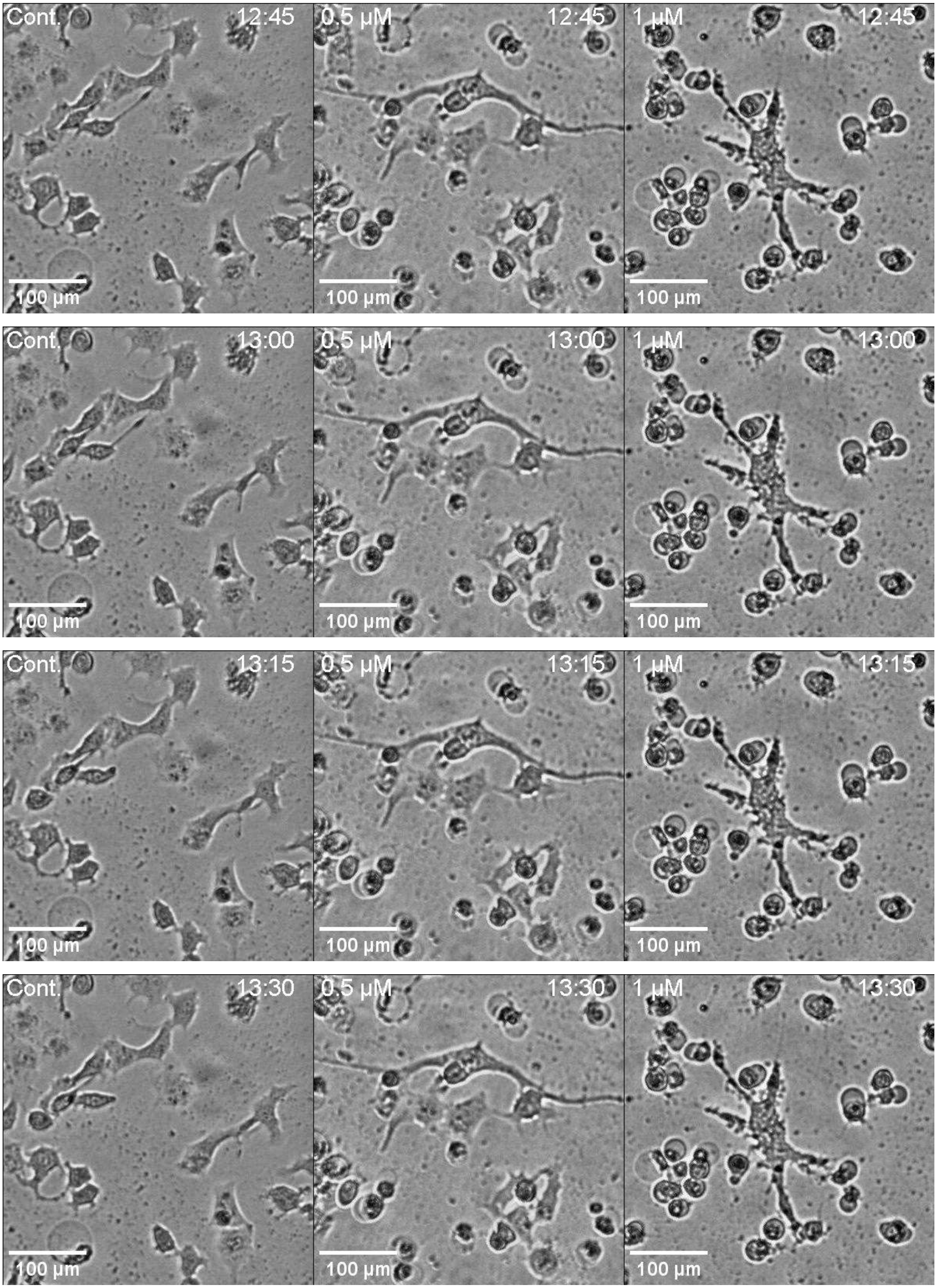
Cont.
12:45
0.5 μM
12:45
1 μM
12:45
Cont.
13:00
0.5 μM
13:00
1 μM
13:00
Cont.
13:15
0.5 μM
13:15
1 μM
13:15
Cont.
13:30
0.5 μM
13:30
1 μM
13:30
100 μm

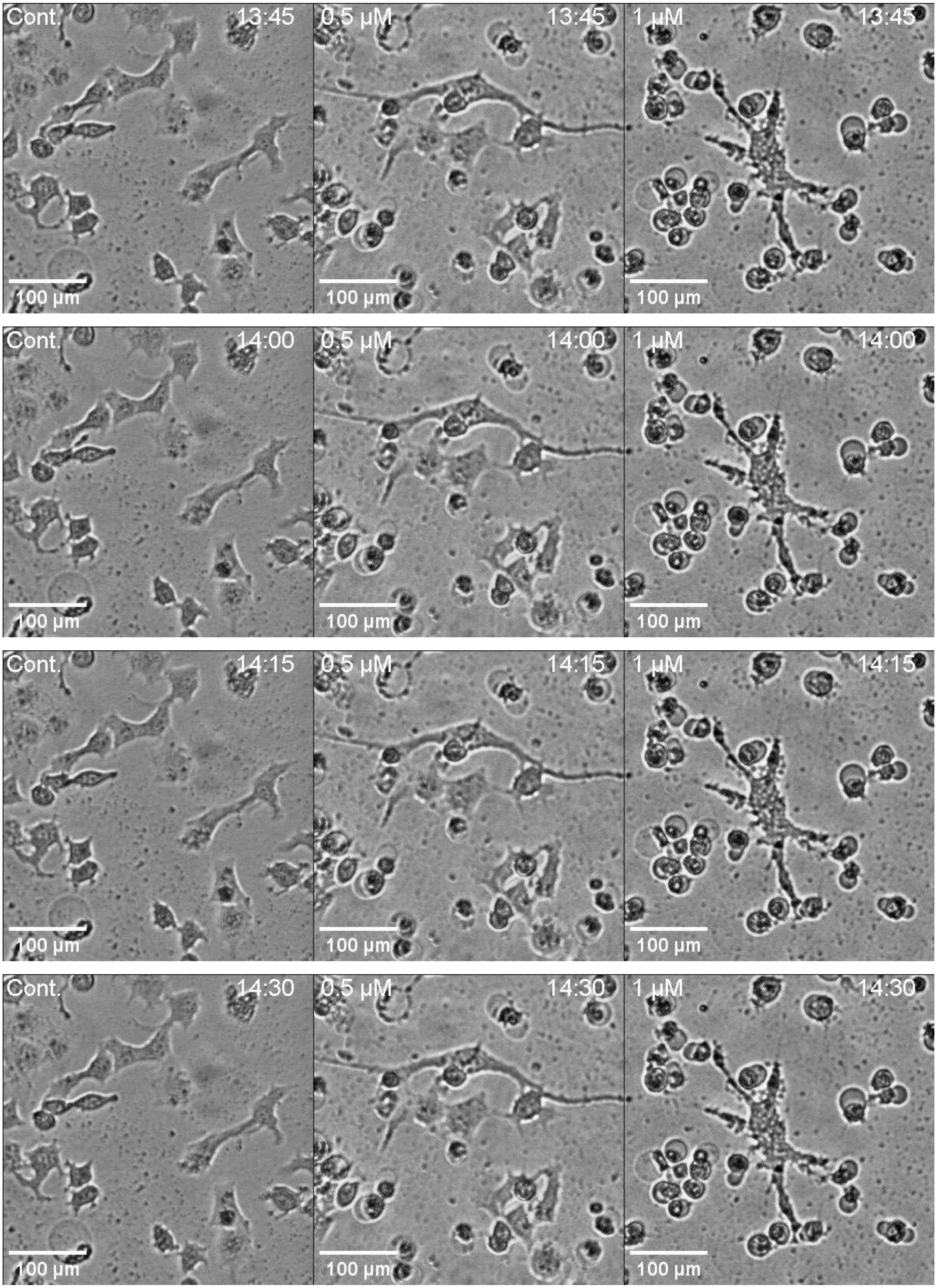
Cont.
13:45
100 µm
0.5 µM
13:45
100 µm
1 µM
13:45
100 µm
Cont.
14:00
100 µm
0.5 µM
14:00
100 µm
1 µM
14:00
100 µm
Cont.
14:15
100 µm
0.5 µM
14:15
100 µm
1 µM
14:15
100 µm
Cont.
14:30
100 µm
0.5 µM
14:30
100 µm
1 µM
14:30
100 µm

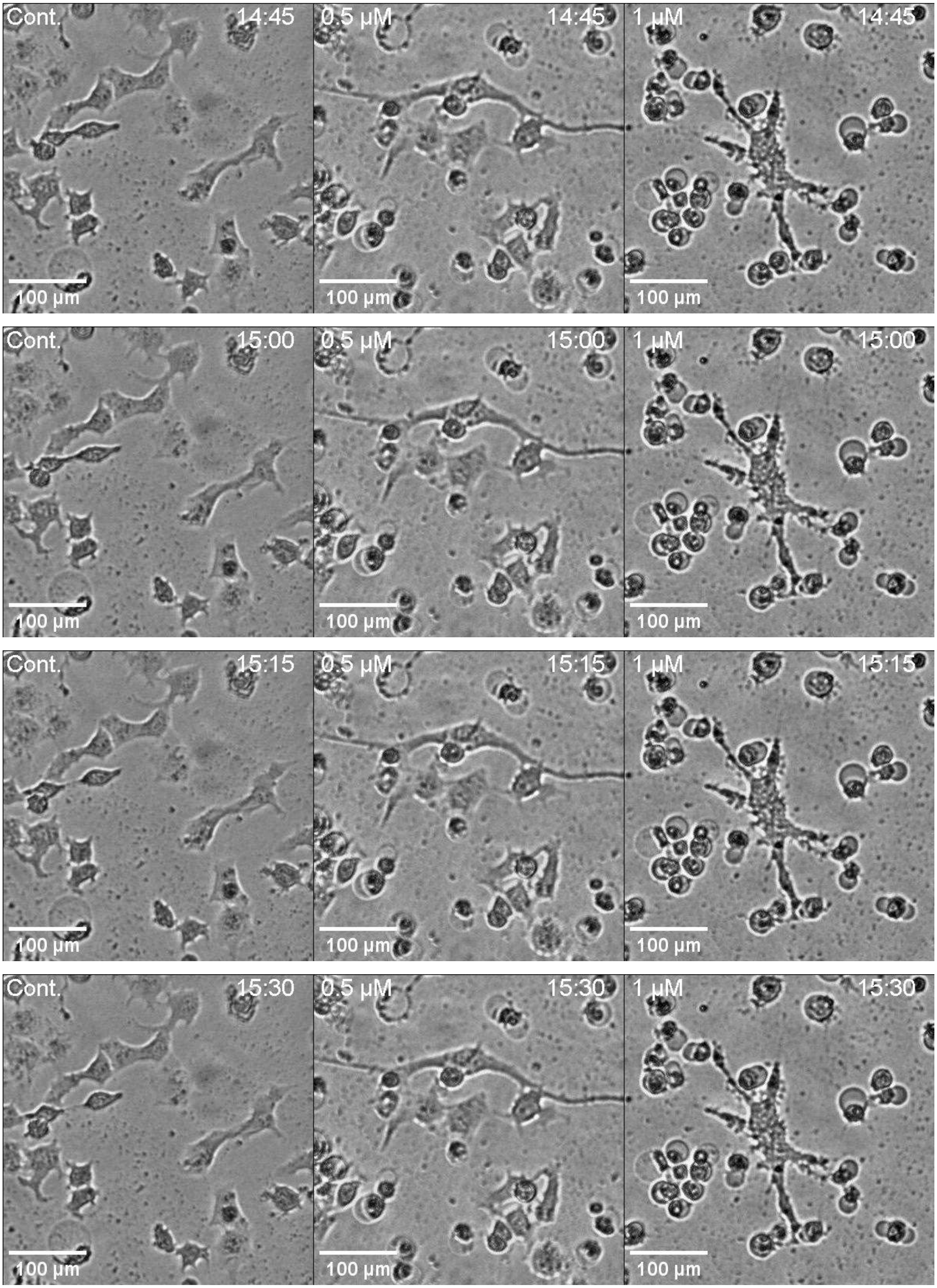
Cont.
14:45
0.5 μM
14:45
1 μM
14:45
100 μm
15:00
15:15
15:30

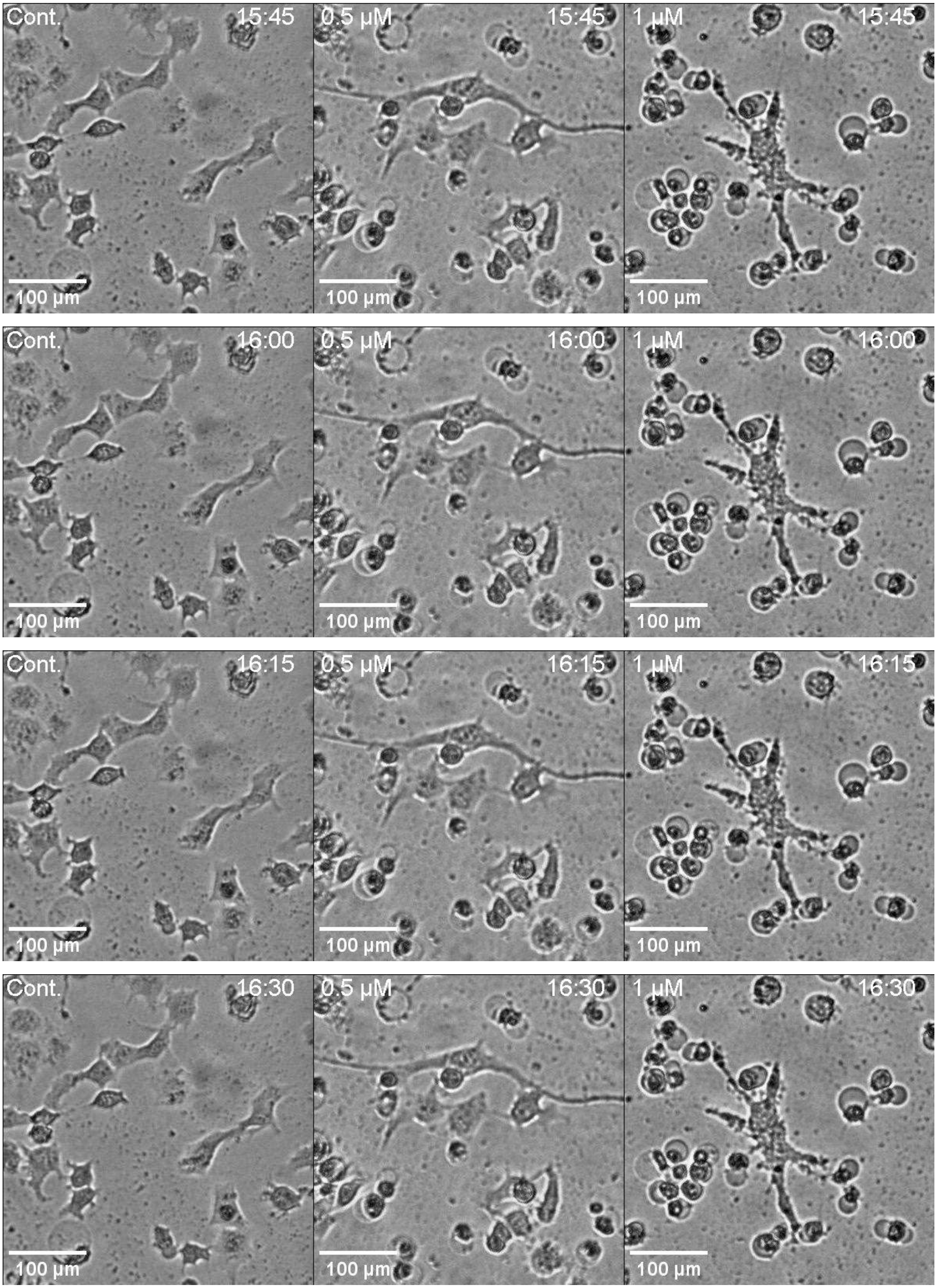
Cont.
15:45
0.5 μM
15:45
1 μM
15:45
100 μm
Cont.
16:00
0.5 μM
16:00
1 μM
16:00
100 μm
Cont.
16:15
0.5 μM
16:15
1 μM
16:15
100 μm
Cont.
16:30
0.5 μM
16:30
1 μM
16:30
100 μm

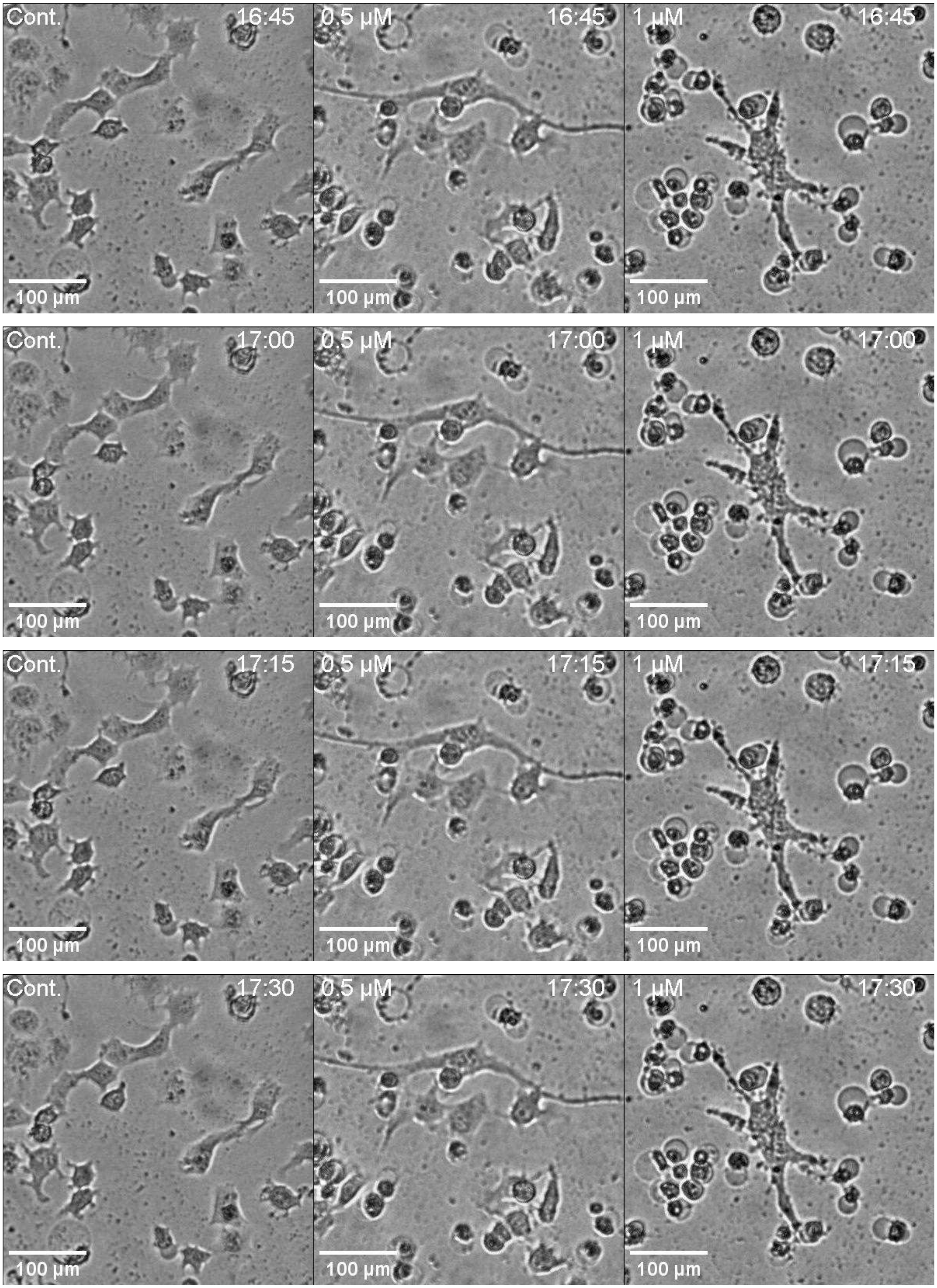
Cont.
16:45
100 µm
0.5 µM
16:45
100 µm
1 µM
16:45
100 µm
Cont.
17:00
100 µm
0.5 µM
17:00
100 µm
1 µM
17:00
100 µm
Cont.
17:15
100 µm
0.5 µM
17:15
100 µm
1 µM
17:15
100 µm
Cont.
17:30
100 µm
0.5 µM
17:30
100 µm
1 µM
17:30
100 µm

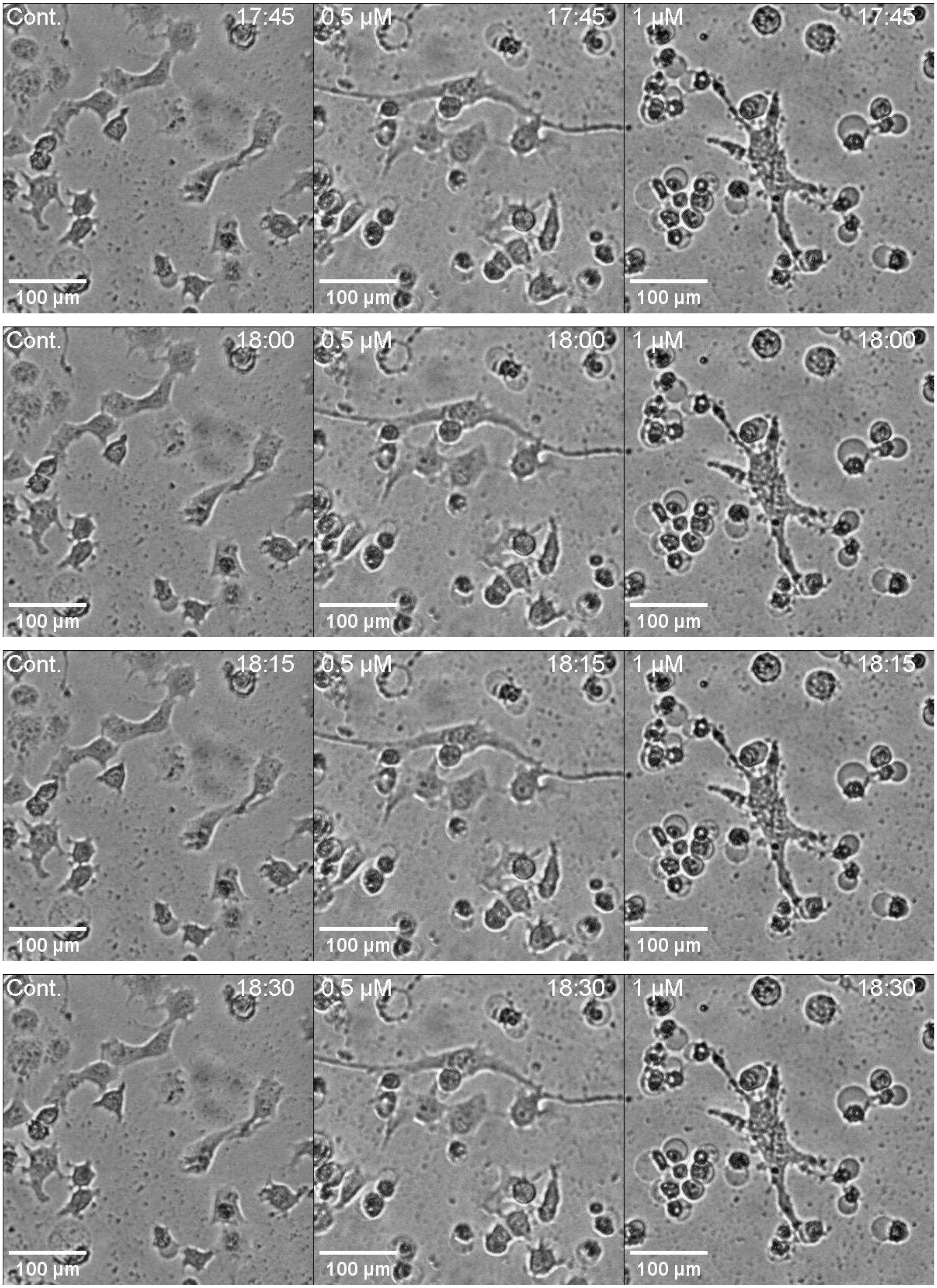

Cont.
17:45
100 μm
0.5 μM
17:45
100 μm
1 μM
17:45
100 μm
Cont.
18:00
100 μm
0.5 μM
18:00
100 μm
1 μM
18:00
100 μm
Cont.
18:15
100 μm
0.5 μM
18:15
100 μm
1 μM
18:15
100 μm
Cont.
18:30
100 μm
0.5 μM
18:30
100 μm
1 μM
18:30
100 μm

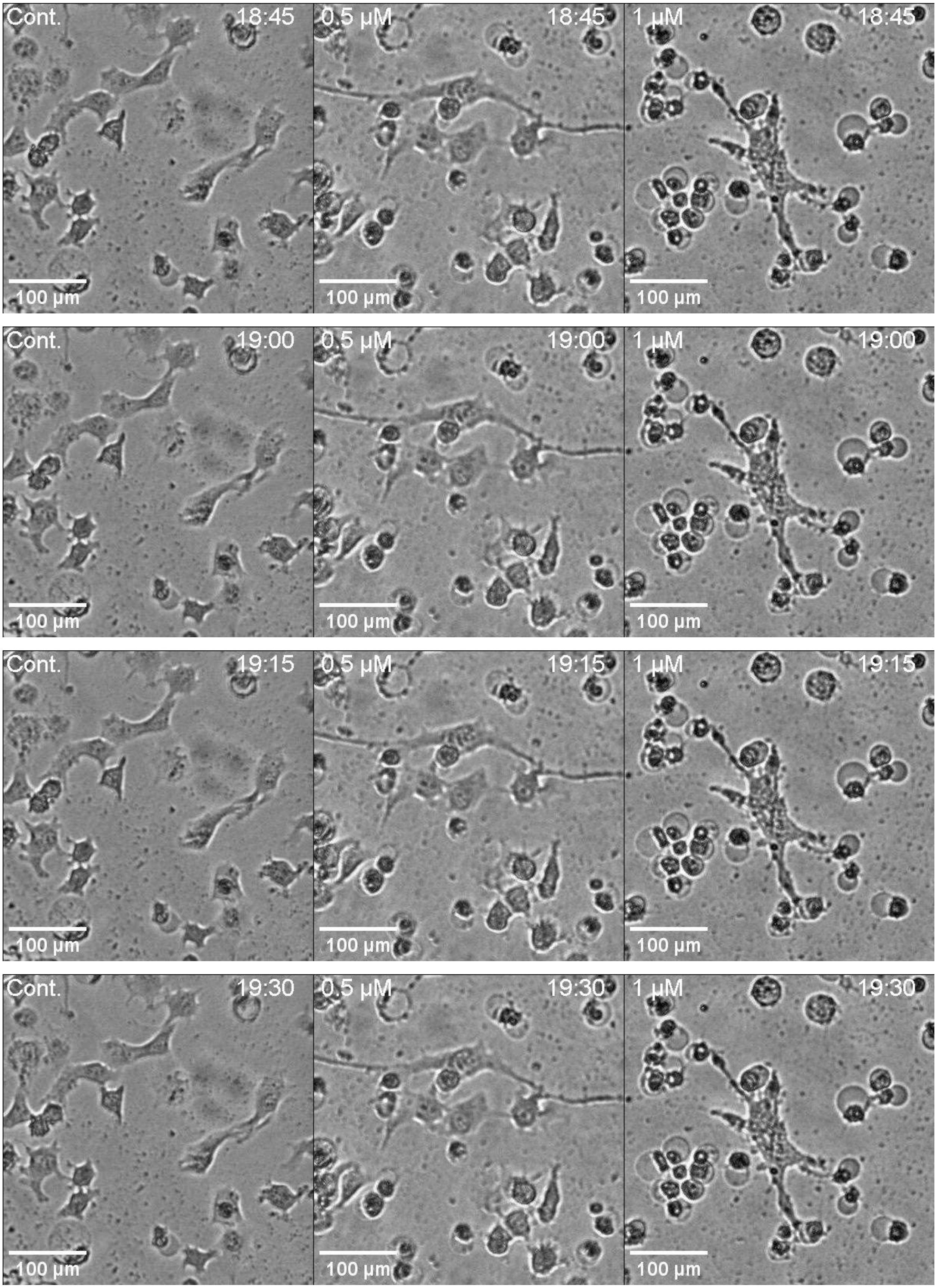
Cont.
18:45
100 µm
0.5 µM
18:45
100 µm
1 µM
18:45
100 µm
Cont.
19:00
100 µm
0.5 µM
19:00
100 µm
1 µM
19:00
100 µm
Cont.
19:15
100 µm
0.5 µM
19:15
100 µm
1 µM
19:15
100 µm
Cont.
19:30
100 µm
0.5 µM
19:30
100 µm
1 µM
19:30
100 µm

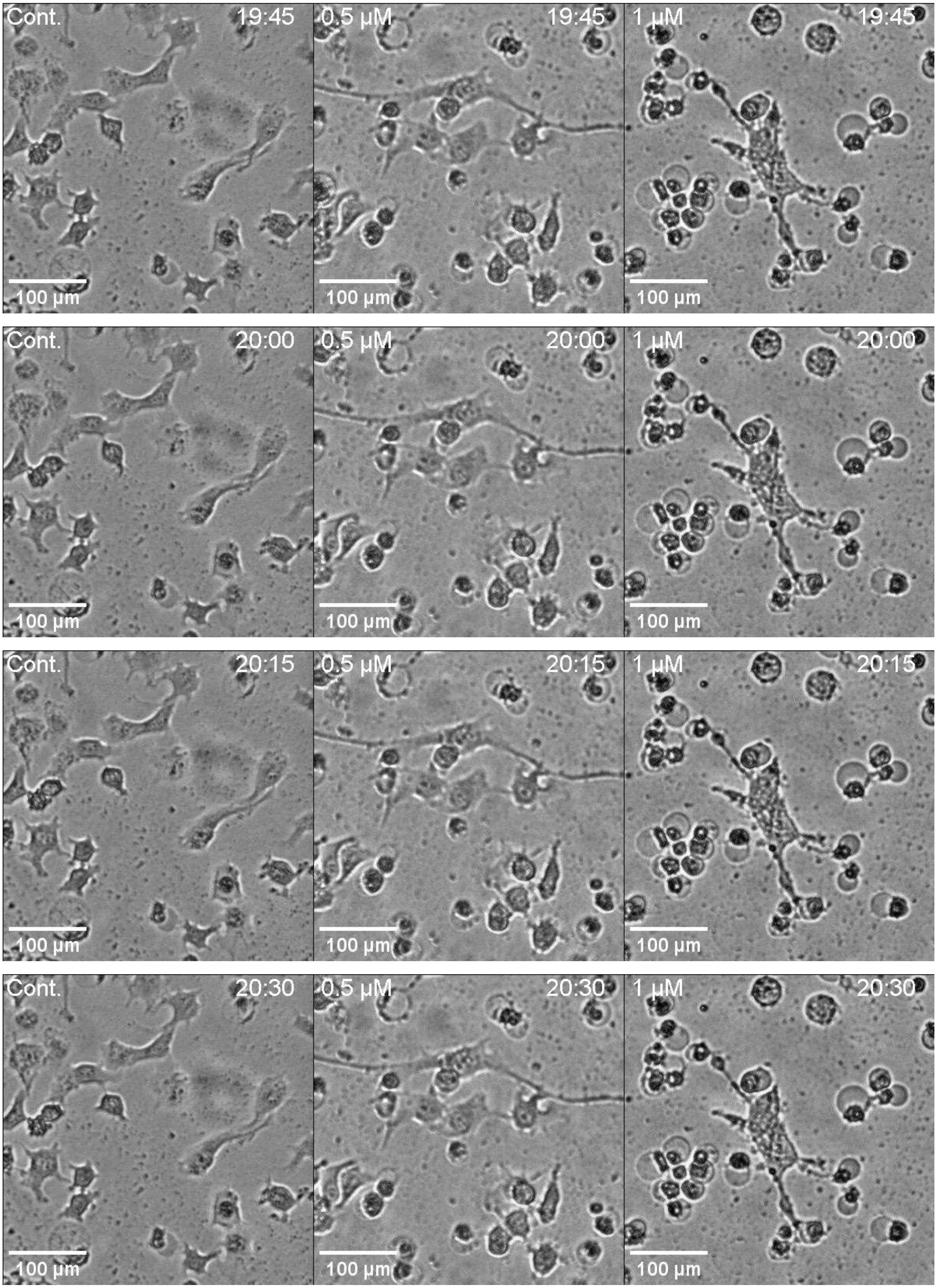
Cont.
19:45
0.5 µM
19:45
1 µM
19:45
20:00
20:15
20:30
100 µm

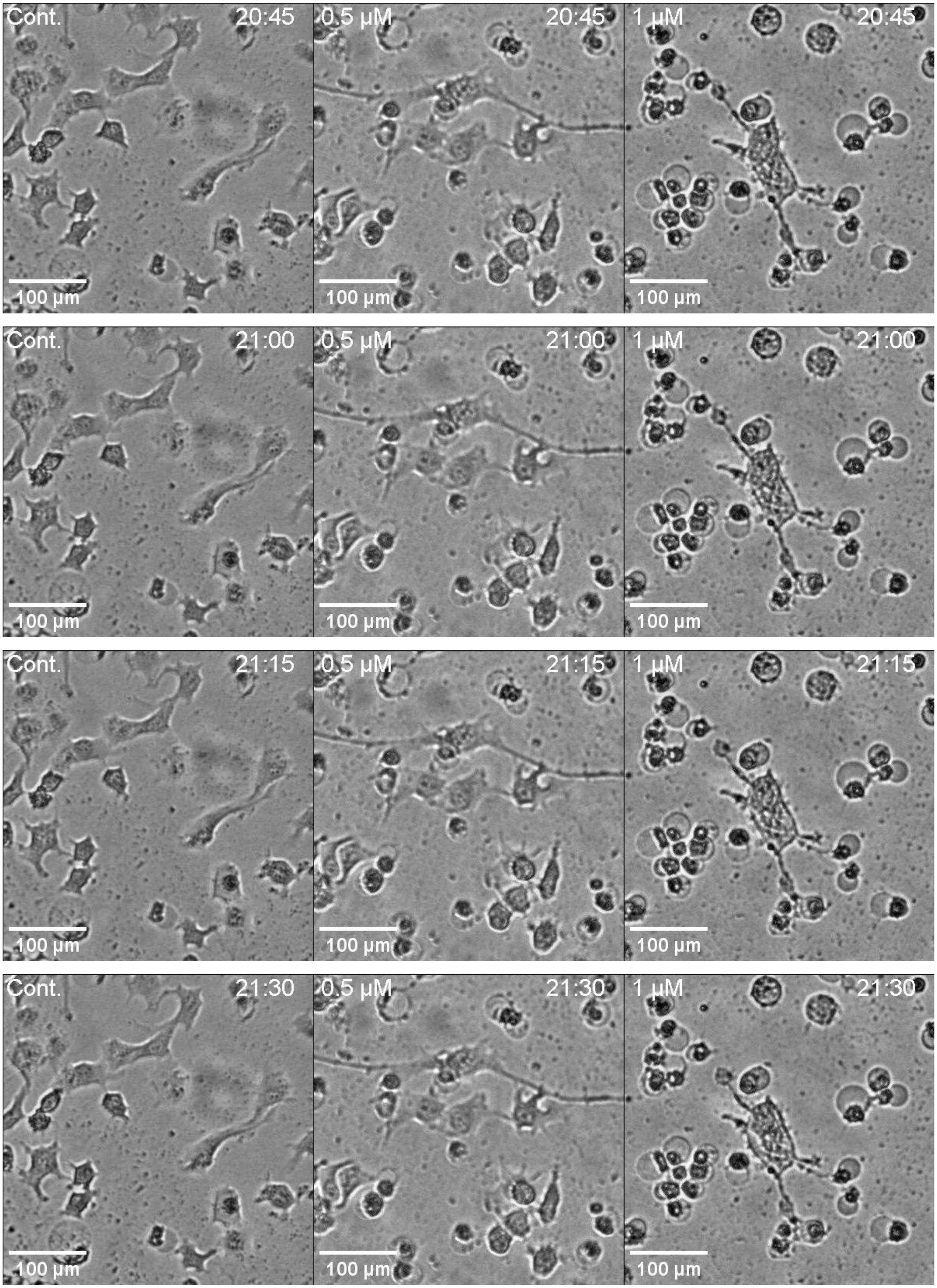
Cont.
20:45
0.5 μM
20:45
1 μM
20:45
21:00
21:15
21:30
100 μm

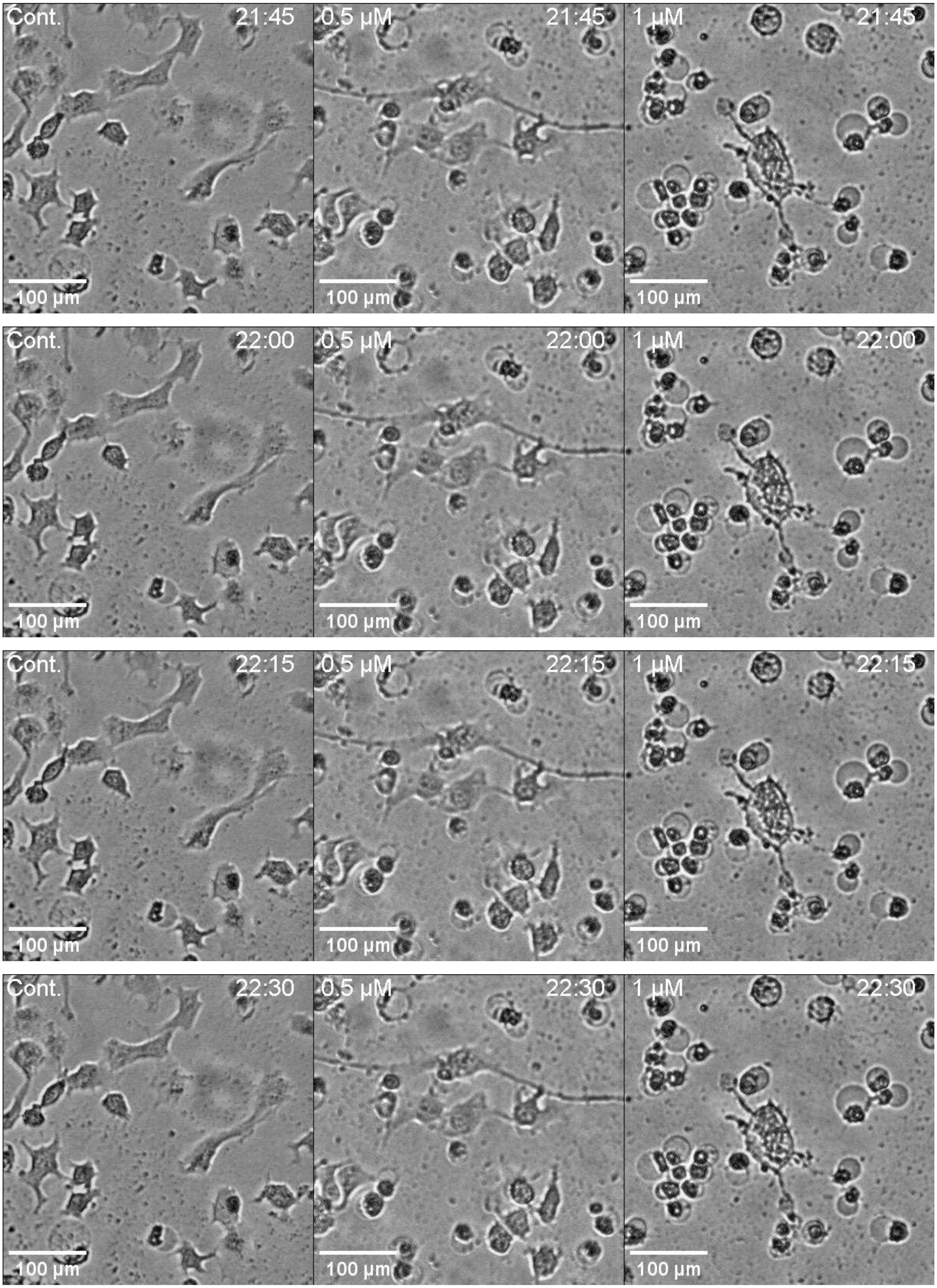

Cont.
21:45
0.5 μM
21:45
1 μM
21:45
100 μm
100 μm
100 μm
Cont.
22:00
0.5 μM
22:00
1 μM
22:00
100 μm
100 μm
100 μm
Cont.
22:15
0.5 μM
22:15
1 μM
22:15
100 μm
100 μm
100 μm
Cont.
22:30
0.5 μM
22:30
1 μM
22:30
100 μm
100 μm
100 μm

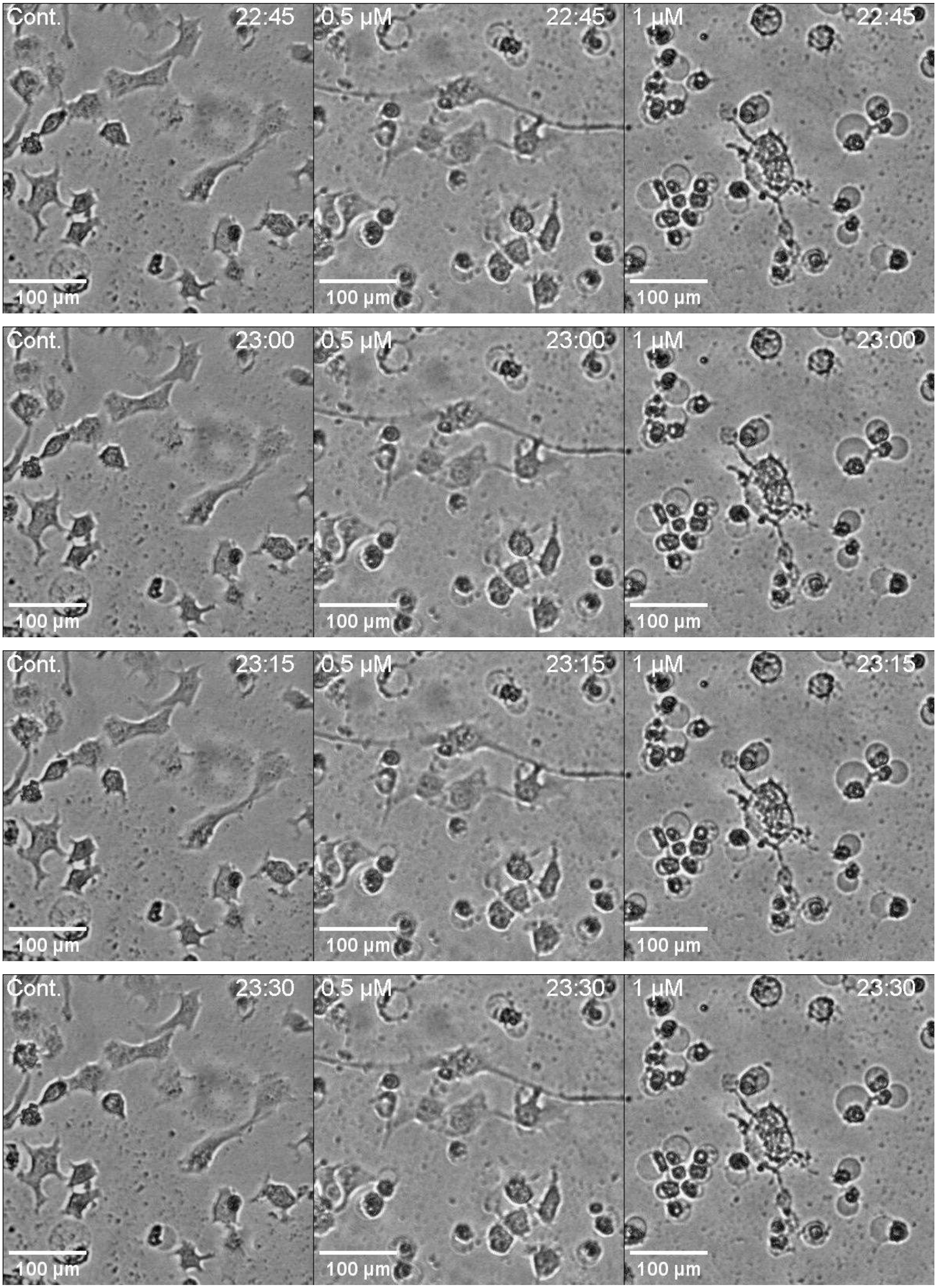
Cont.
22:45
100 µm
0.5 µM
22:45
100 µm
1 µM
22:45
100 µm
Cont.
23:00
100 µm
0.5 µM
23:00
100 µm
1 µM
23:00
100 µm
Cont.
23:15
100 µm
0.5 µM
23:15
100 µm
1 µM
23:15
100 µm
Cont.
23:30
100 µm
0.5 µM
23:30
100 µm
1 µM
23:30
100 µm

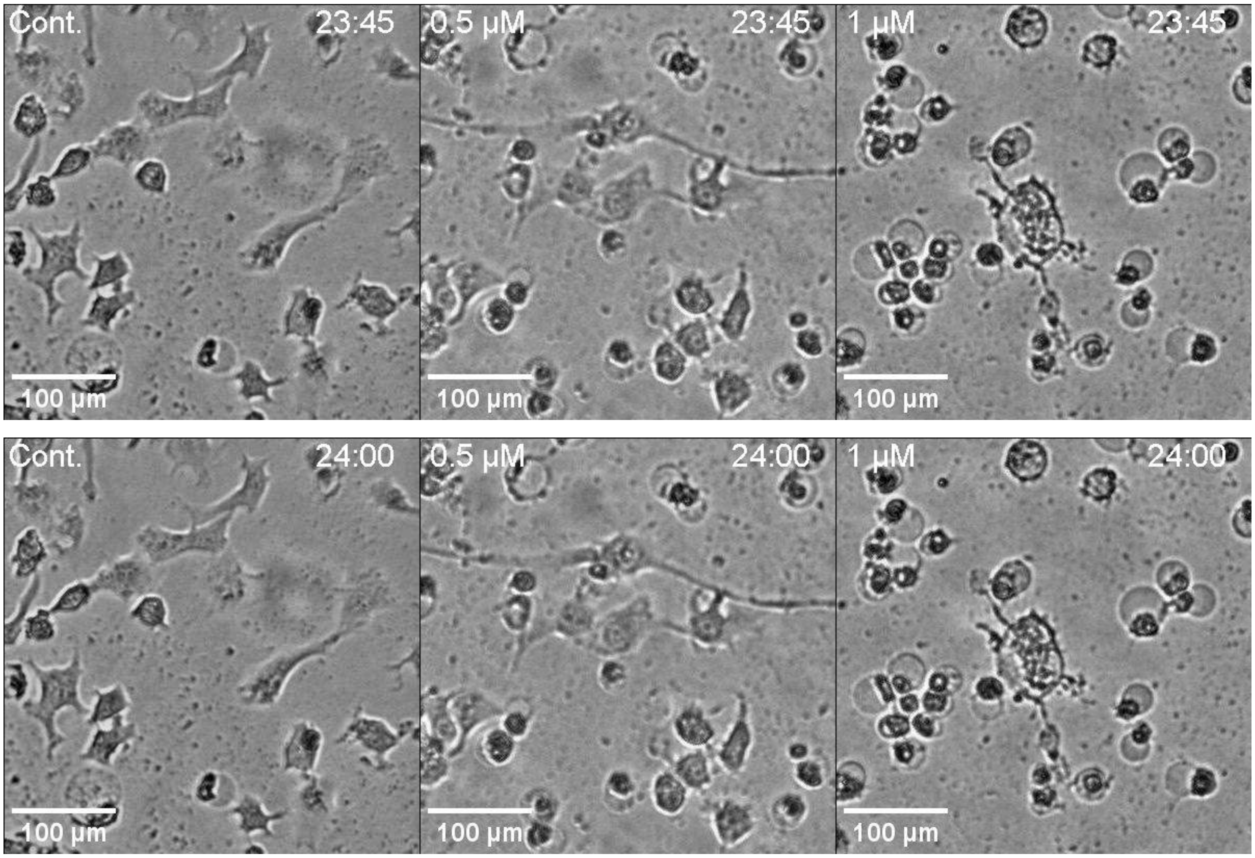
Cont.
23:45
100 µm
0.5 µM
23:45
100 µm
1 µM
23:45
100 µm
Cont.
24:00
100 µm
0.5 µM
24:00
100 µm
1 µM
24:00
100 µm

Figure S4. Real-time cell migration assay of calliviminone A (CVM-A)-induced inhibition of PANC-1 pancreatic cancer cell migration.

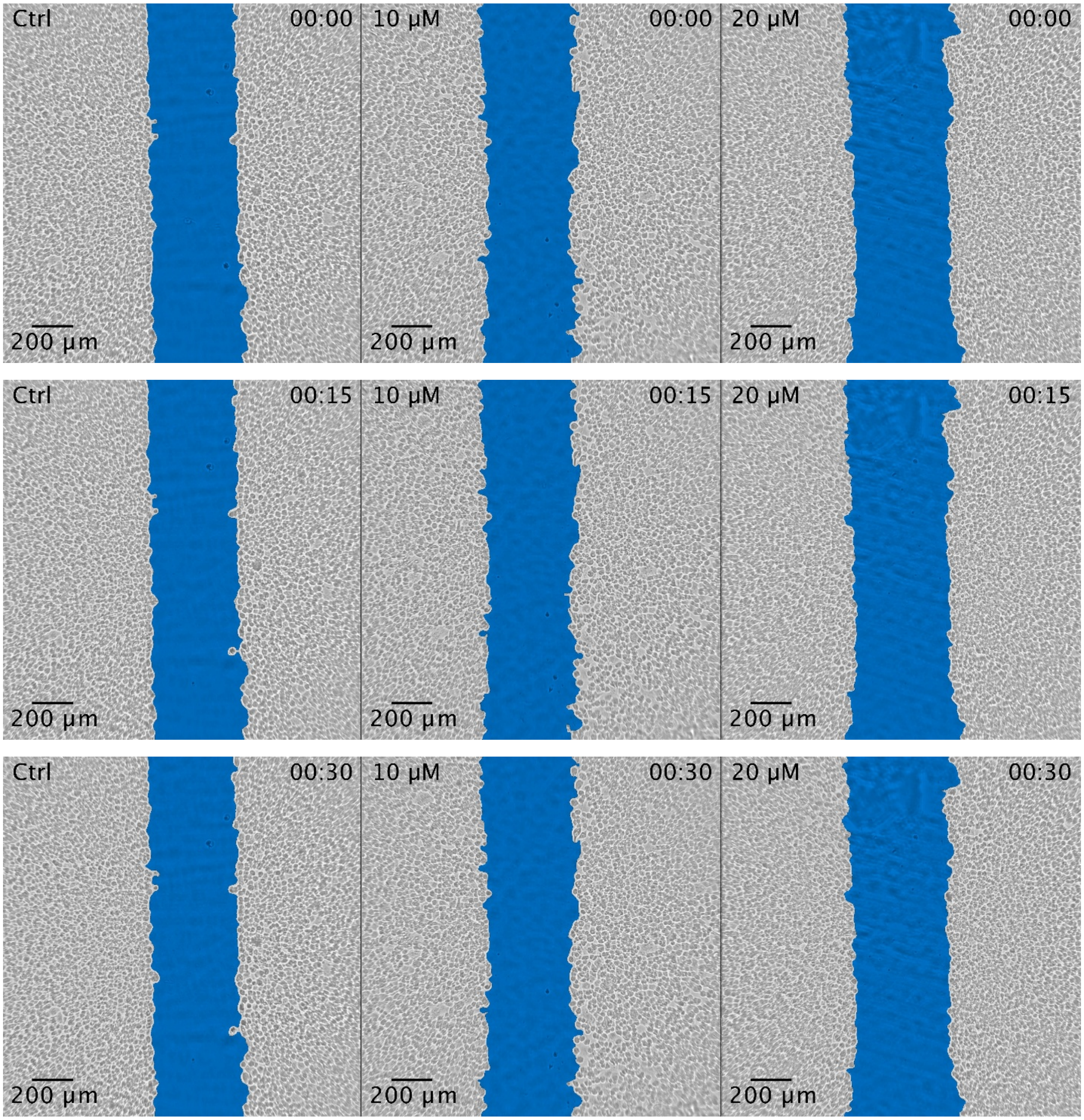

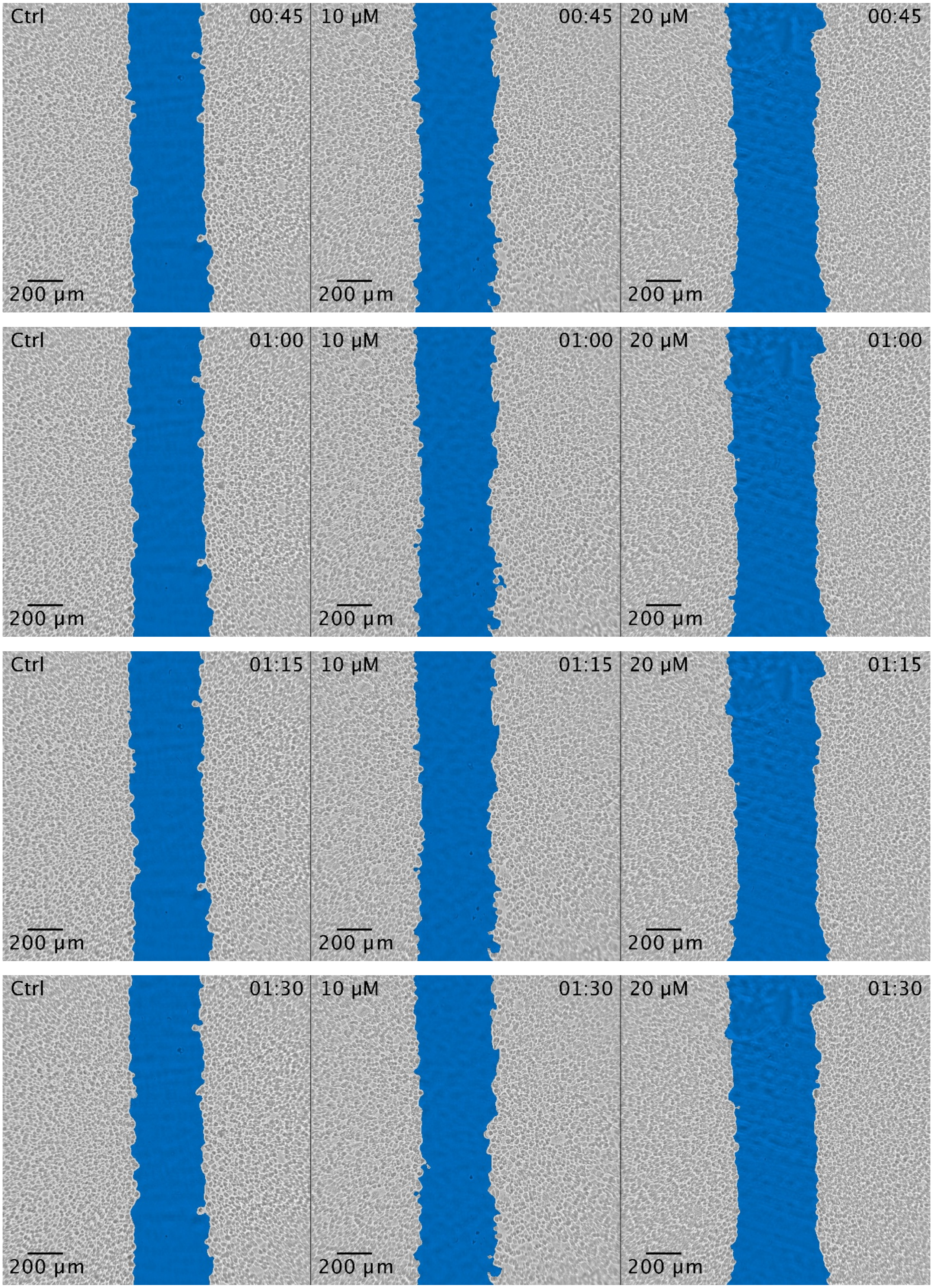
Ctrl
00:45
10 μM
00:45
20 μM
00:45
200 μm
Ctrl
01:00
10 μM
01:00
20 μM
01:00
200 μm
Ctrl
01:15
10 μM
01:15
20 μM
01:15
200 μm
Ctrl
01:30
10 μM
01:30
20 μM
01:30
200 μm

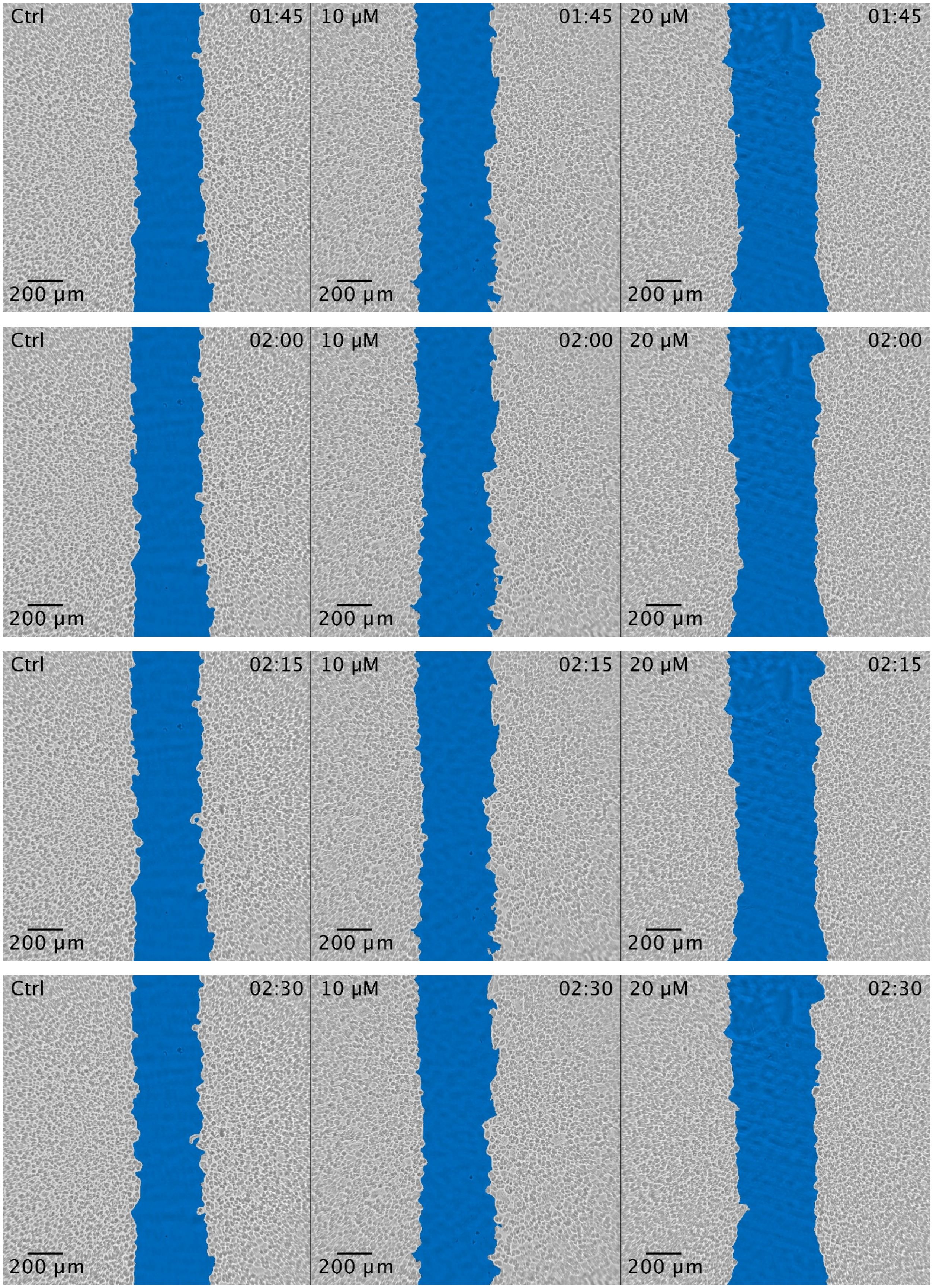
Ctrl
01:45
10 µM
01:45
20 µM
01:45
200 µm
Ctrl
02:00
10 µM
02:00
20 µM
02:00
Ctrl
02:15
10 µM
02:15
20 µM
02:15
Ctrl
02:30
10 µM
02:30
20 µM
02:30

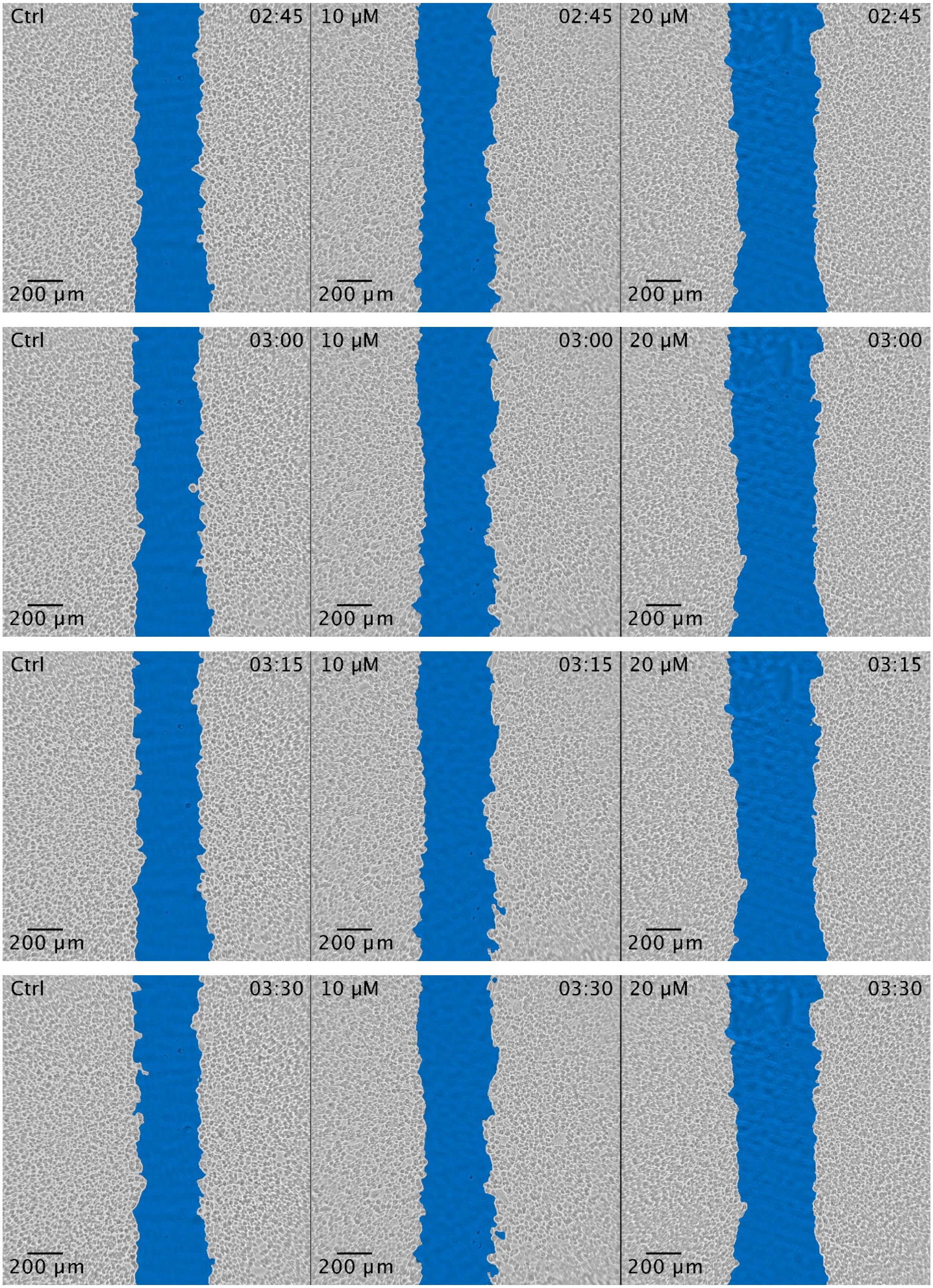
Ctrl
02:45
10 µM
02:45
20 µM
02:45
200 µm
Ctrl
03:00
10 µM
03:00
20 µM
03:00
Ctrl
03:15
10 µM
03:15
20 µM
03:15
Ctrl
03:30
10 µM
03:30
20 µM
03:30

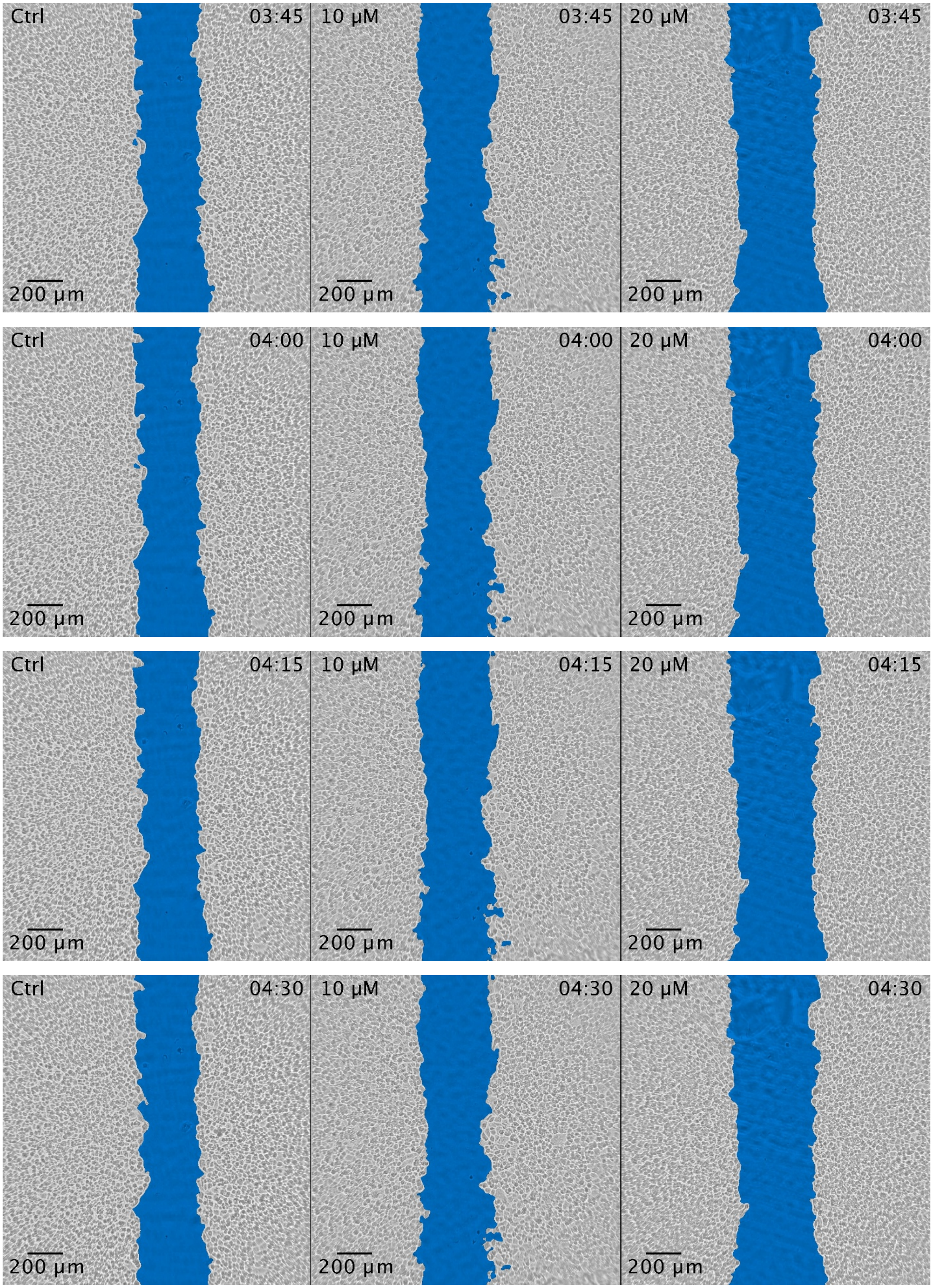
Ctrl
03:45
10 μM
03:45
20 μM
03:45
200 μm
200 μm
200 μm
Ctrl
04:00
10 μM
04:00
20 μM
04:00
200 μm
200 μm
200 μm
Ctrl
04:15
10 μM
04:15
20 μM
04:15
200 μm
200 μm
200 μm
Ctrl
04:30
10 μM
04:30
20 μM
04:30
200 μm
200 μm
200 μm

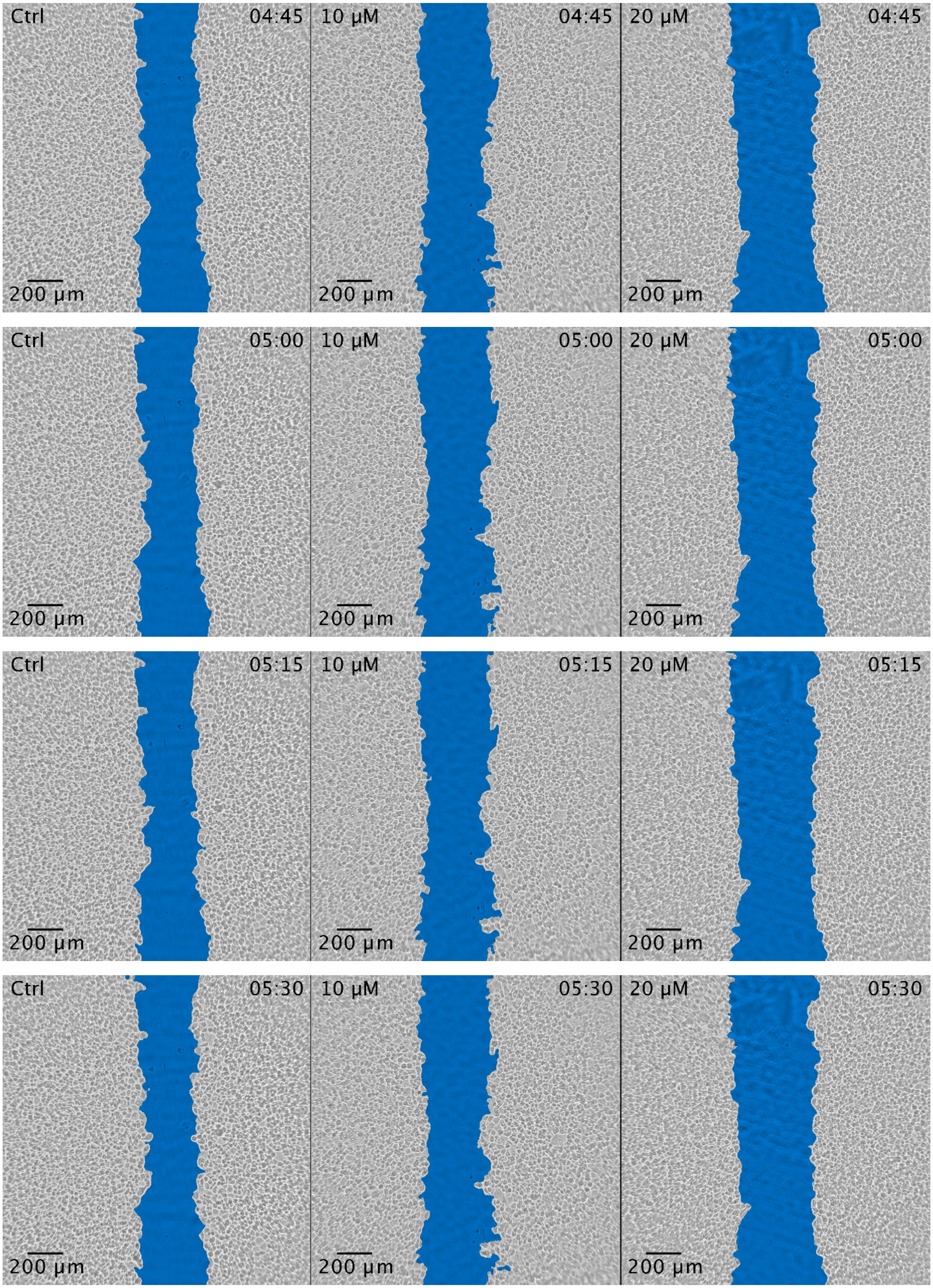
Ctrl
04:45
10 μM
04:45
20 μM
04:45
200 μm
200 μm
200 μm
Ctrl
05:00
10 μM
05:00
20 μM
05:00
200 μm
200 μm
200 μm
Ctrl
05:15
10 μM
05:15
20 μM
05:15
200 μm
200 μm
200 μm
Ctrl
05:30
10 μM
05:30
20 μM
05:30
200 μm
200 μm
200 μm

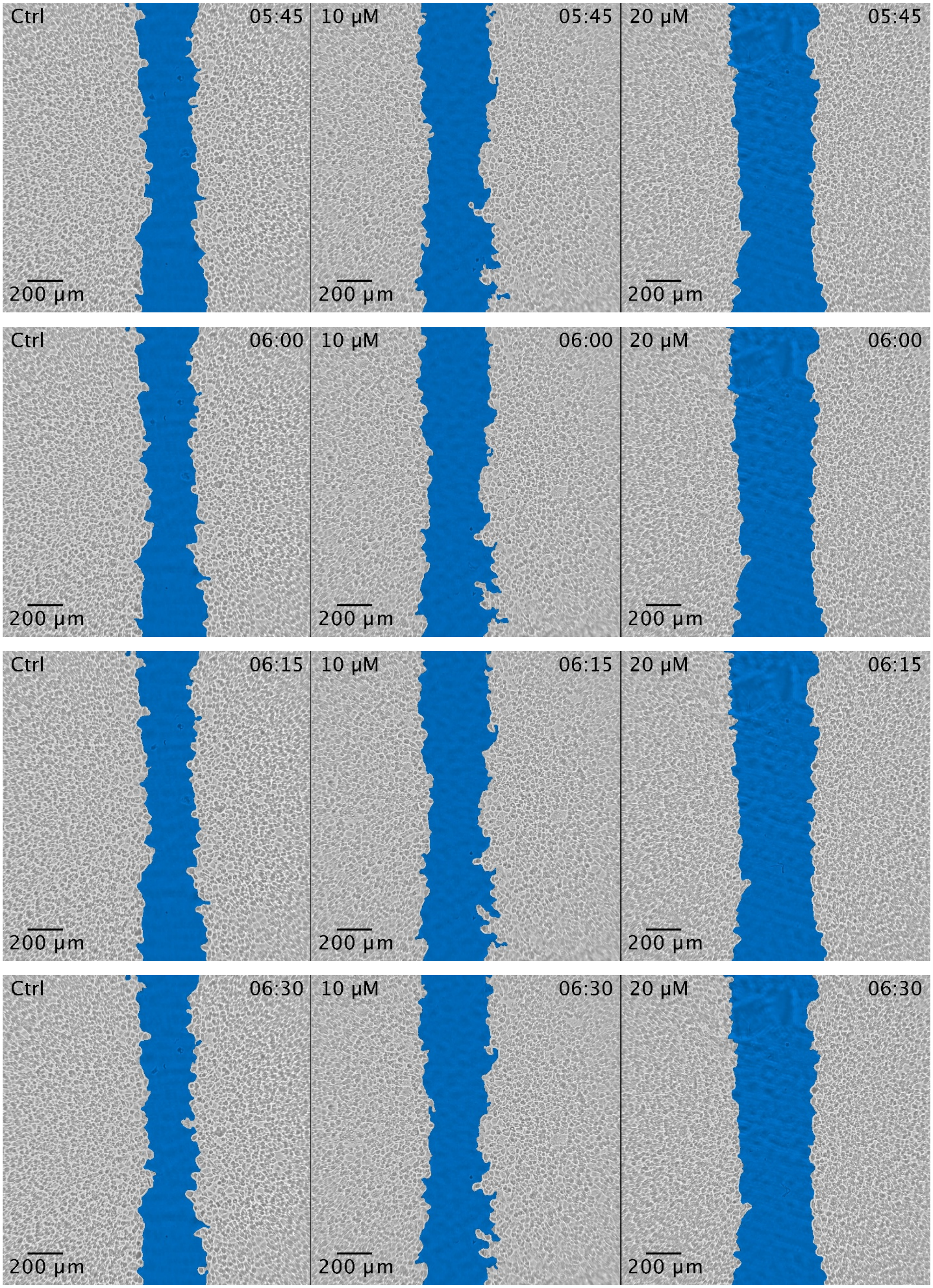
Ctrl
05:45
10 µM
05:45
20 µM
05:45
200 µm
Ctrl
06:00
10 µM
06:00
20 µM
06:00
200 µm
Ctrl
06:15
10 µM
06:15
20 µM
06:15
200 µm
Ctrl
06:30
10 µM
06:30
20 µM
06:30
200 µm

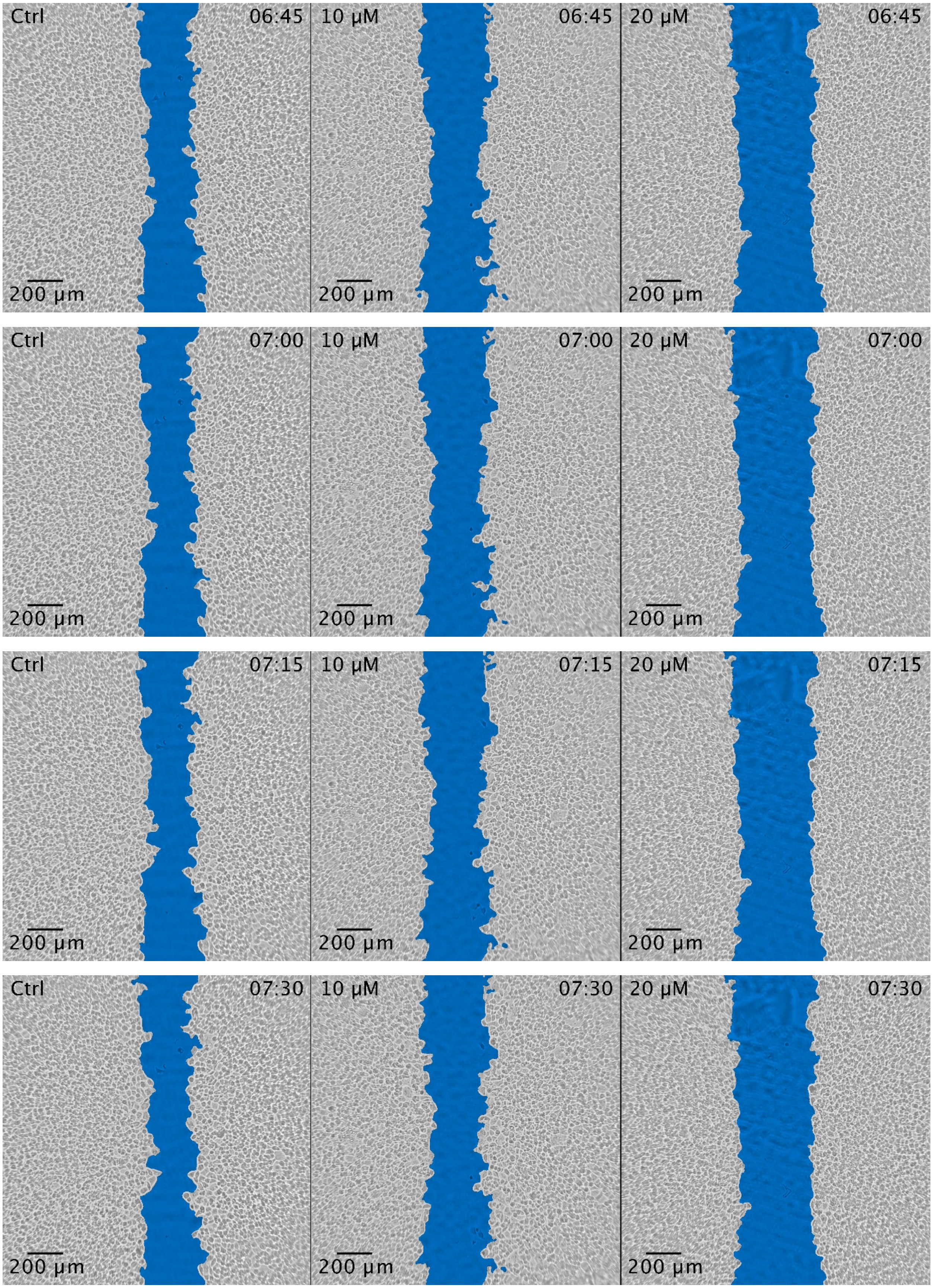
Ctrl
06:45
10 μM
06:45
20 μM
06:45
200 μm
Ctrl
07:00
10 μM
07:00
20 μM
07:00
200 μm
Ctrl
07:15
10 μM
07:15
20 μM
07:15
200 μm
Ctrl
07:30
10 μM
07:30
20 μM
07:30
200 μm

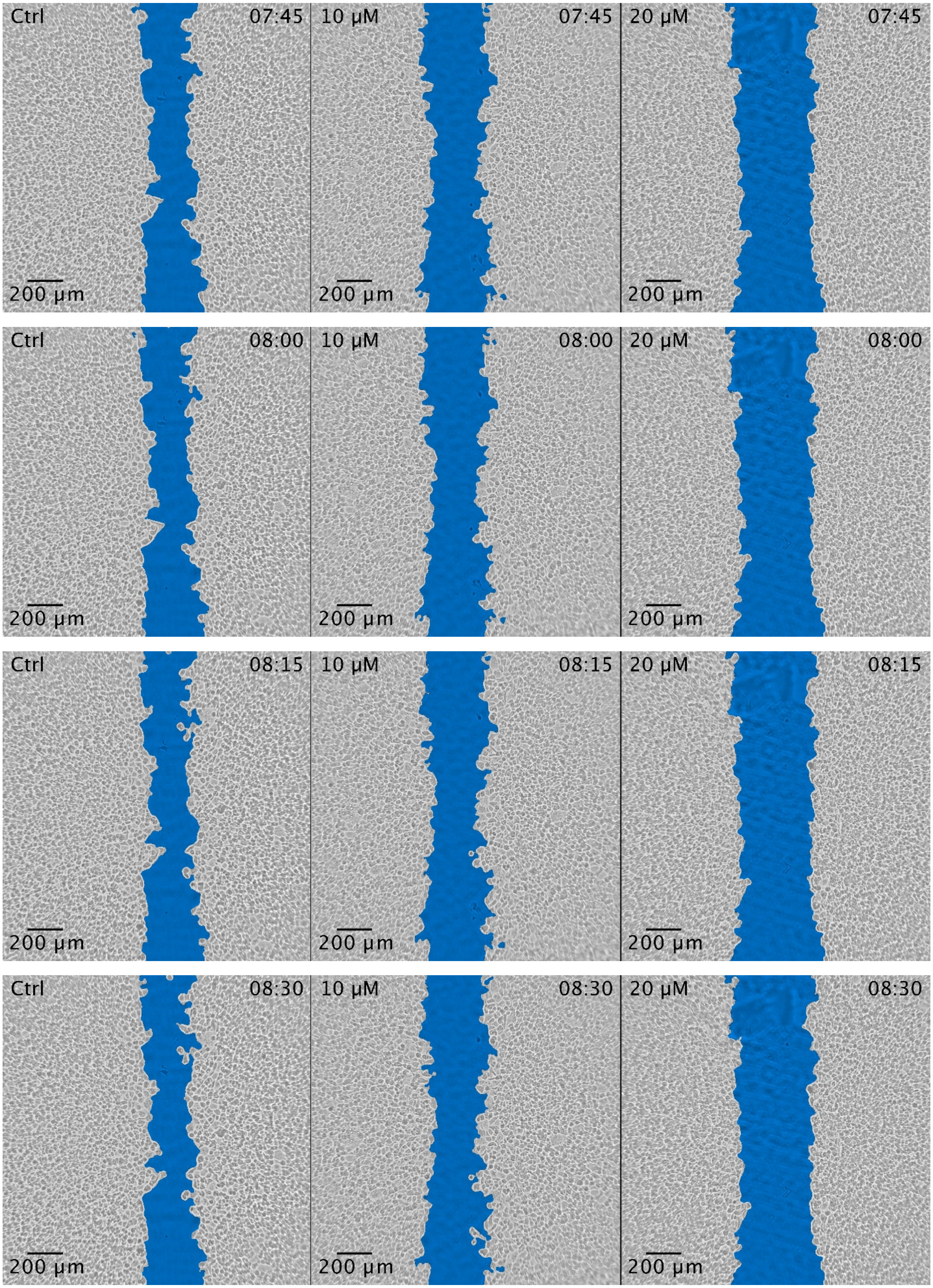
Ctrl
07:45
10 µM
07:45
20 µM
07:45
200 µm
Ctrl
08:00
10 µM
08:00
20 µM
08:00
200 µm
Ctrl
08:15
10 µM
08:15
20 µM
08:15
200 µm
Ctrl
08:30
10 µM
08:30
20 µM
08:30
200 µm

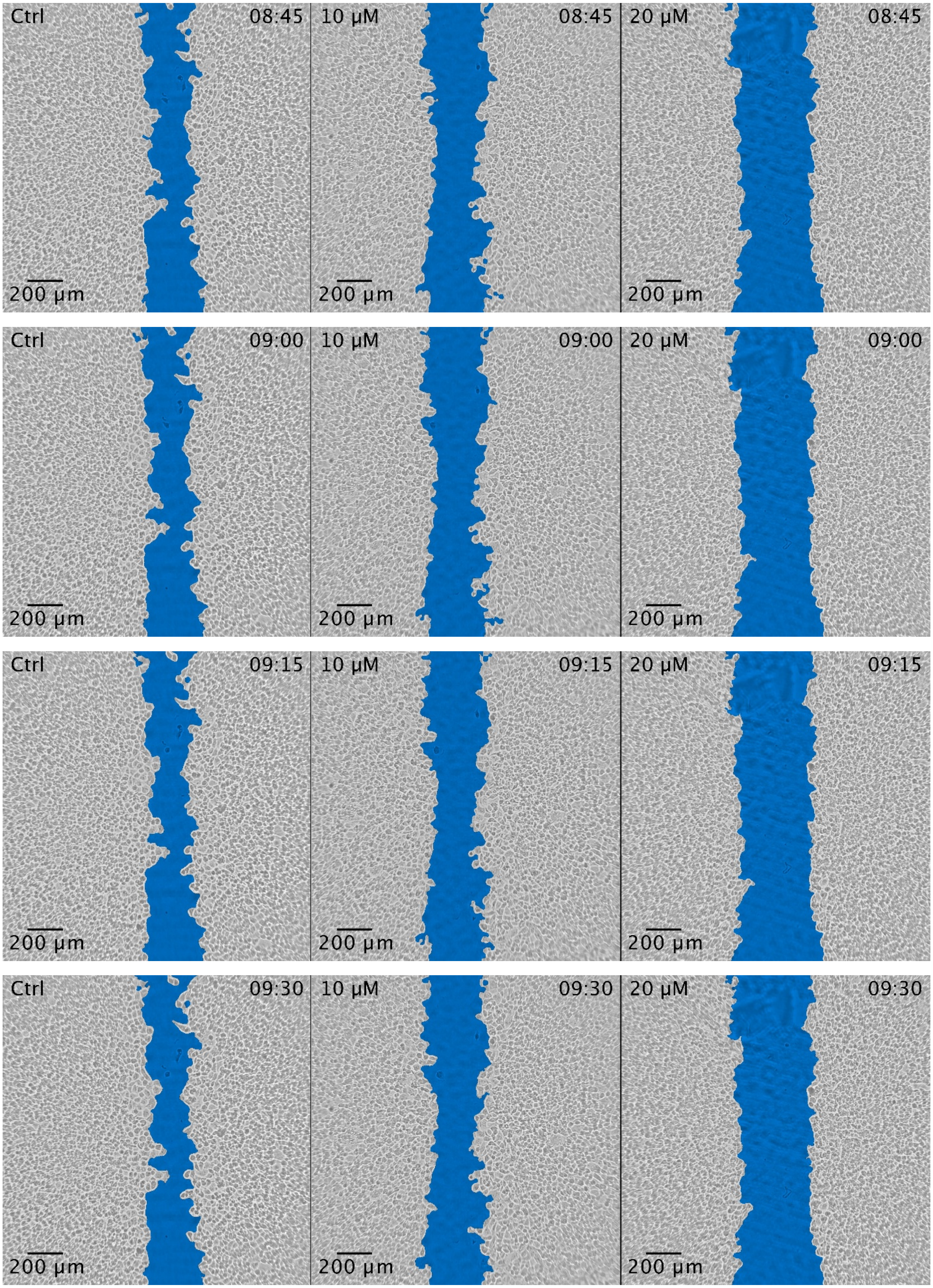
Ctrl
08:45
10 µM
08:45
20 µM
08:45
200 µm
Ctrl
09:00
10 µM
09:00
20 µM
09:00
Ctrl
09:15
10 µM
09:15
20 µM
09:15
Ctrl
09:30
10 µM
09:30
20 µM
09:30

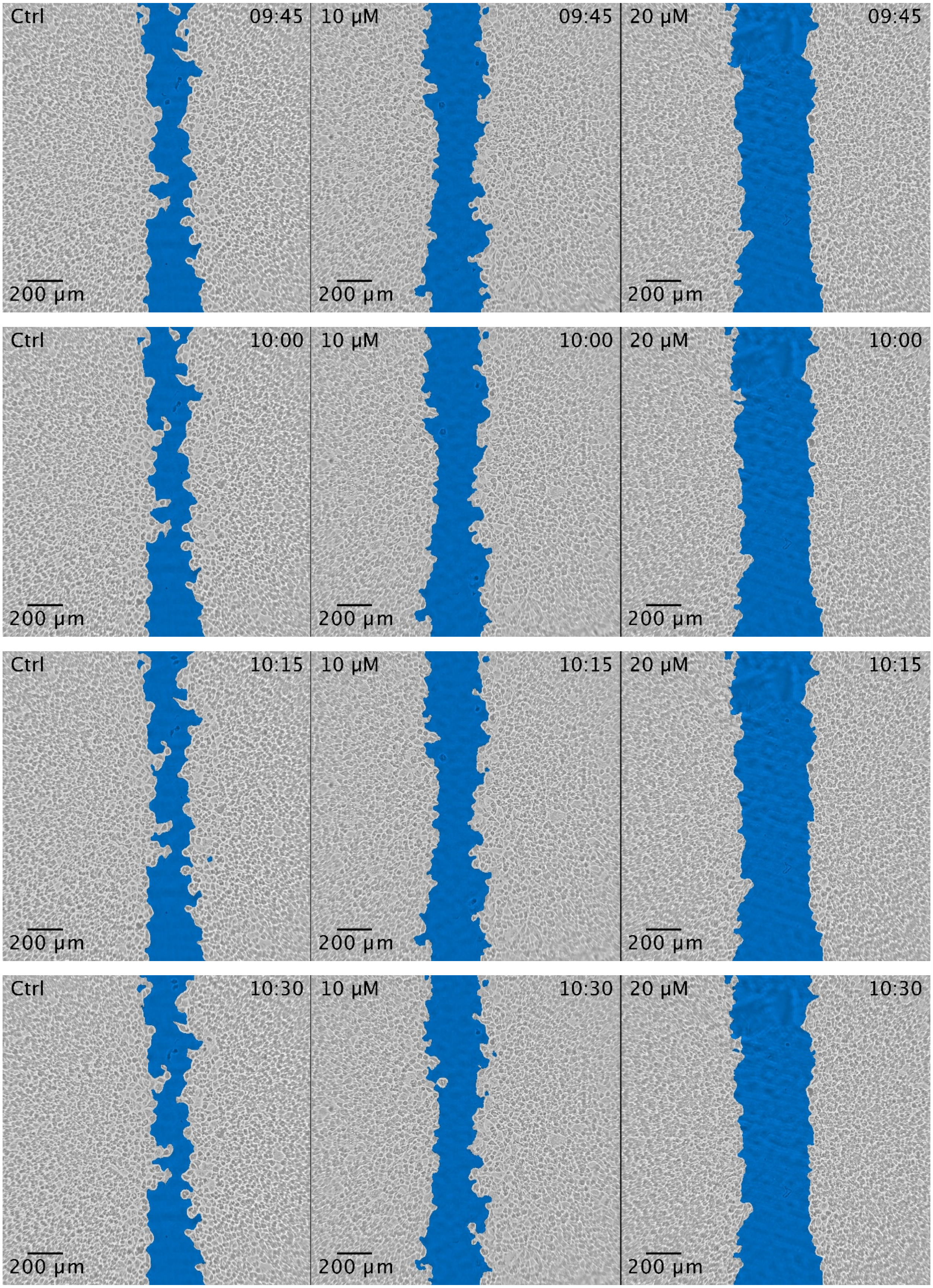
Ctrl
09:45
10 µM
09:45
20 µM
09:45
200 µm
Ctrl
10:00
10 µM
10:00
20 µM
10:00
Ctrl
10:15
10 µM
10:15
20 µM
10:15
Ctrl
10:30
10 µM
10:30
20 µM
10:30

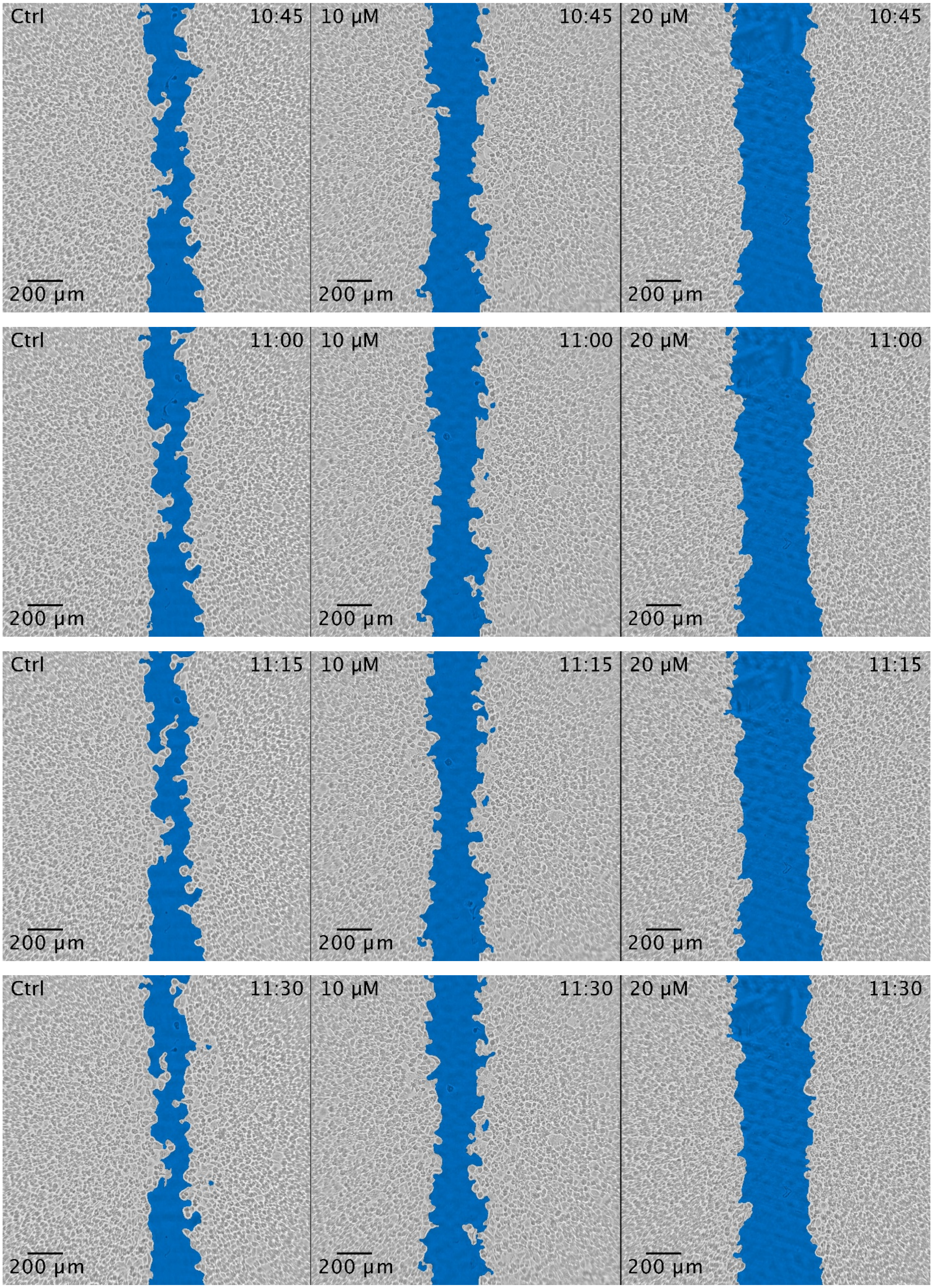
Ctrl
10:45
10 μM
10:45
20 μM
10:45
200 μm
200 μm
200 μm
Ctrl
11:00
10 μM
11:00
20 μM
11:00
200 μm
200 μm
200 μm
Ctrl
11:15
10 μM
11:15
20 μM
11:15
200 μm
200 μm
200 μm
Ctrl
11:30
10 μM
11:30
20 μM
11:30
200 μm
200 μm
200 μm

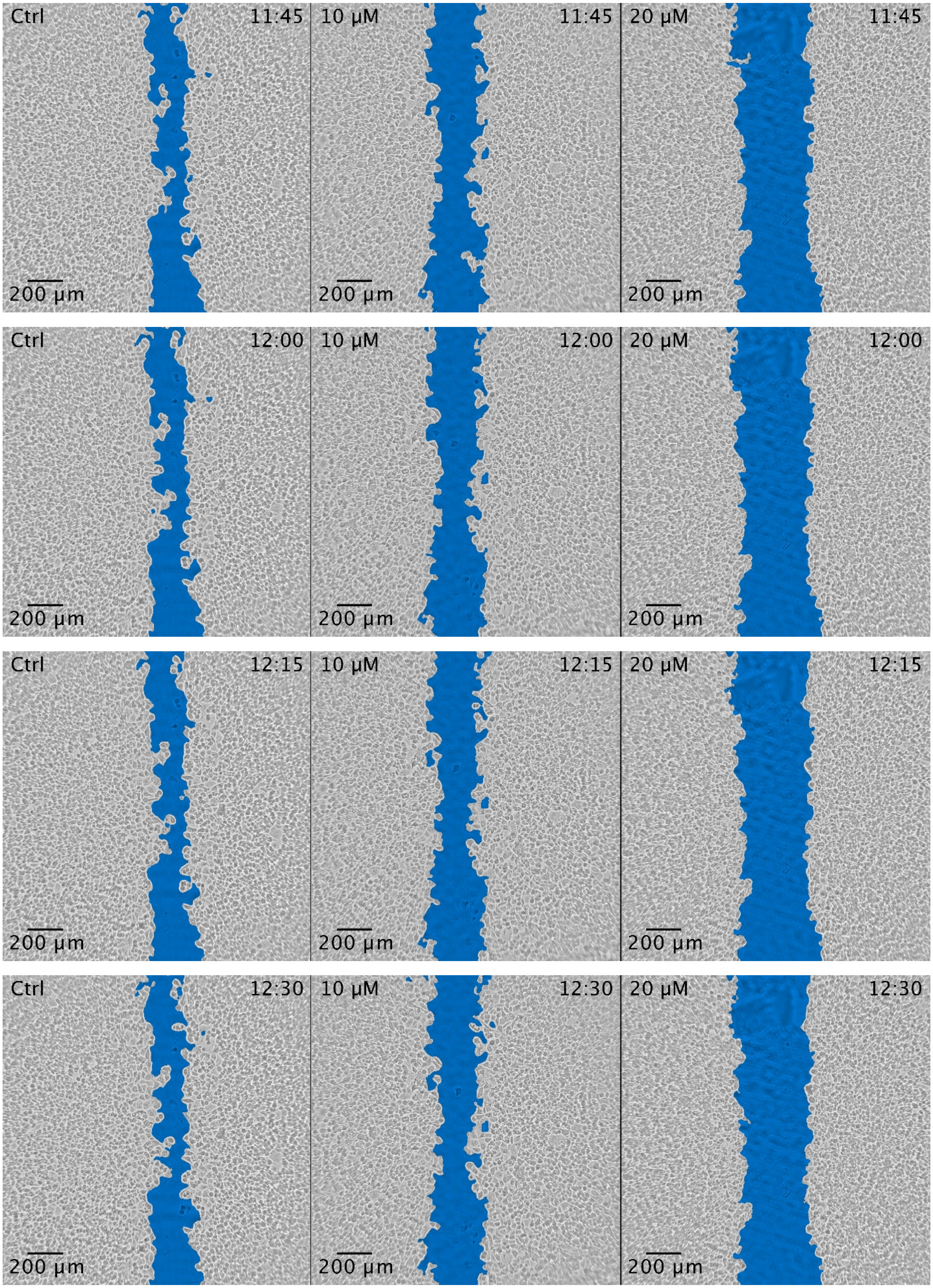
Ctrl
11:45
10 μM
11:45
20 μM
11:45
200 μm
Ctrl
12:00
10 μM
12:00
20 μM
12:00
200 μm
Ctrl
12:15
10 μM
12:15
20 μM
12:15
200 μm
Ctrl
12:30
10 μM
12:30
20 μM
12:30
200 μm

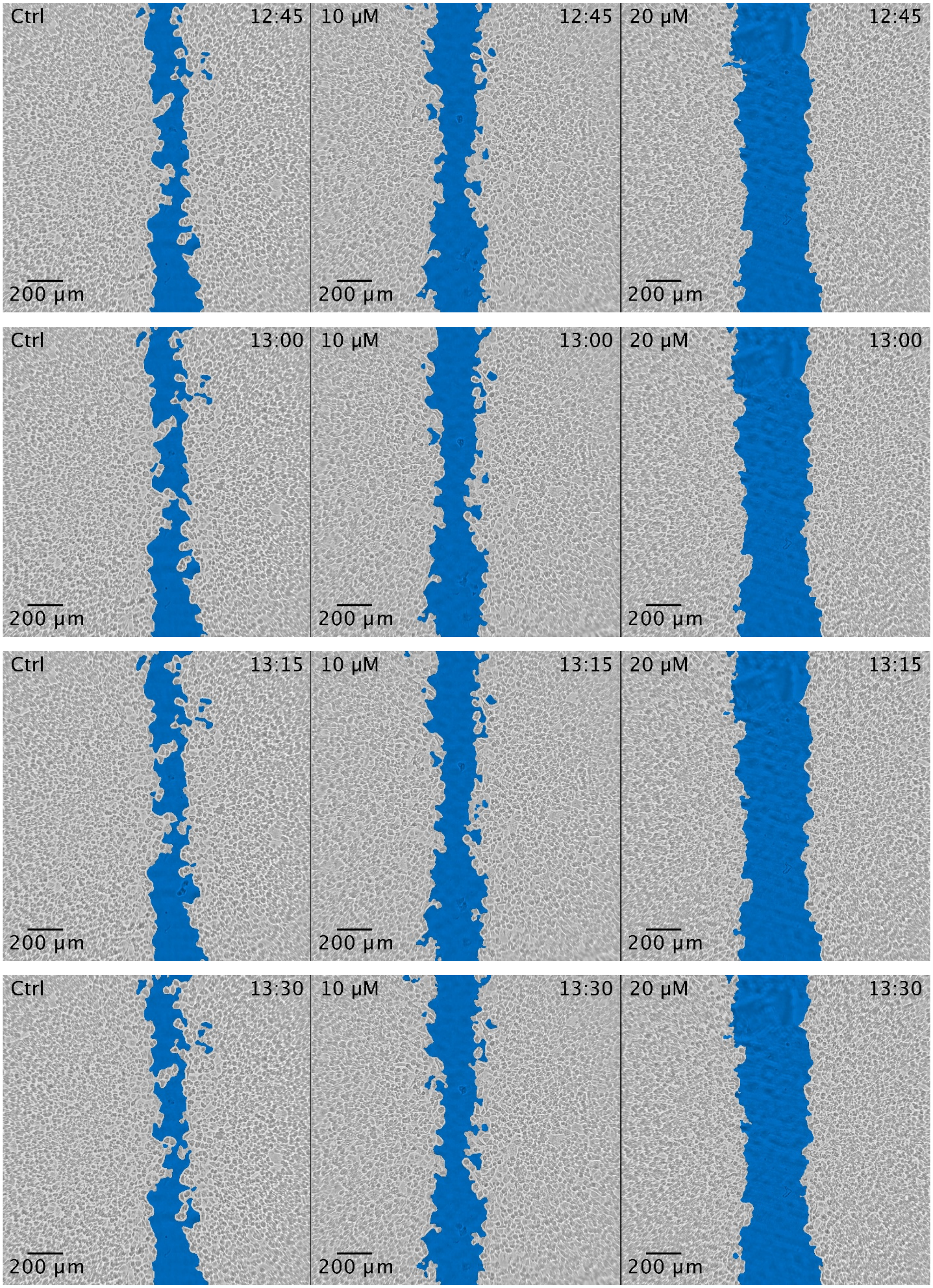
Ctrl
12:45
10 μM
12:45
20 μM
12:45
200 μm
200 μm
200 μm
Ctrl
13:00
10 μM
13:00
20 μM
13:00
200 μm
200 μm
200 μm
Ctrl
13:15
10 μM
13:15
20 μM
13:15
200 μm
200 μm
200 μm
Ctrl
13:30
10 μM
13:30
20 μM
13:30
200 μm
200 μm
200 μm

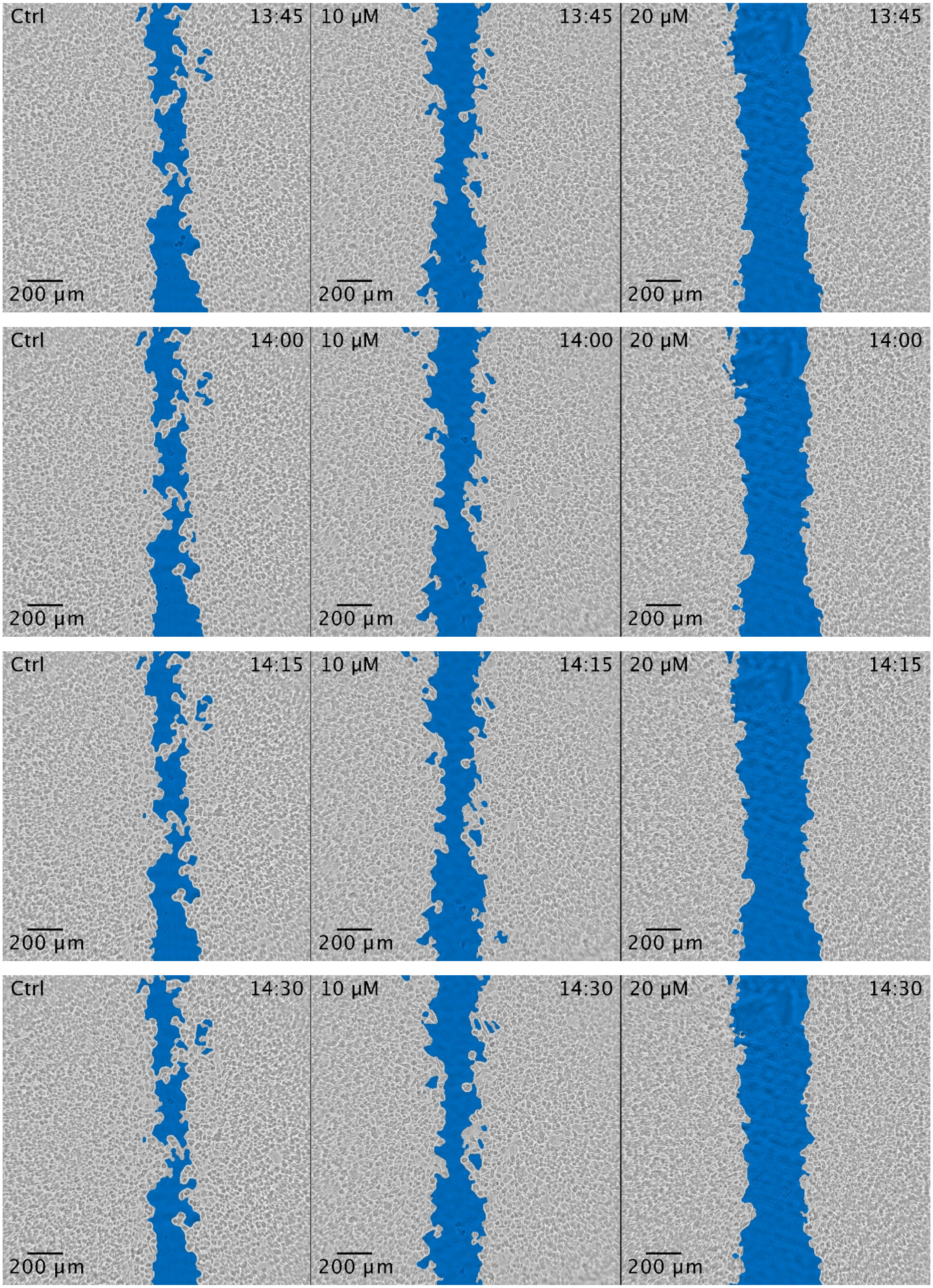
Ctrl
13:45
10 μM
13:45
20 μM
13:45
200 μm
200 μm
200 μm
Ctrl
14:00
10 μM
14:00
20 μM
14:00
200 μm
200 μm
200 μm
Ctrl
14:15
10 μM
14:15
20 μM
14:15
200 μm
200 μm
200 μm
Ctrl
14:30
10 μM
14:30
20 μM
14:30
200 μm
200 μm
200 μm

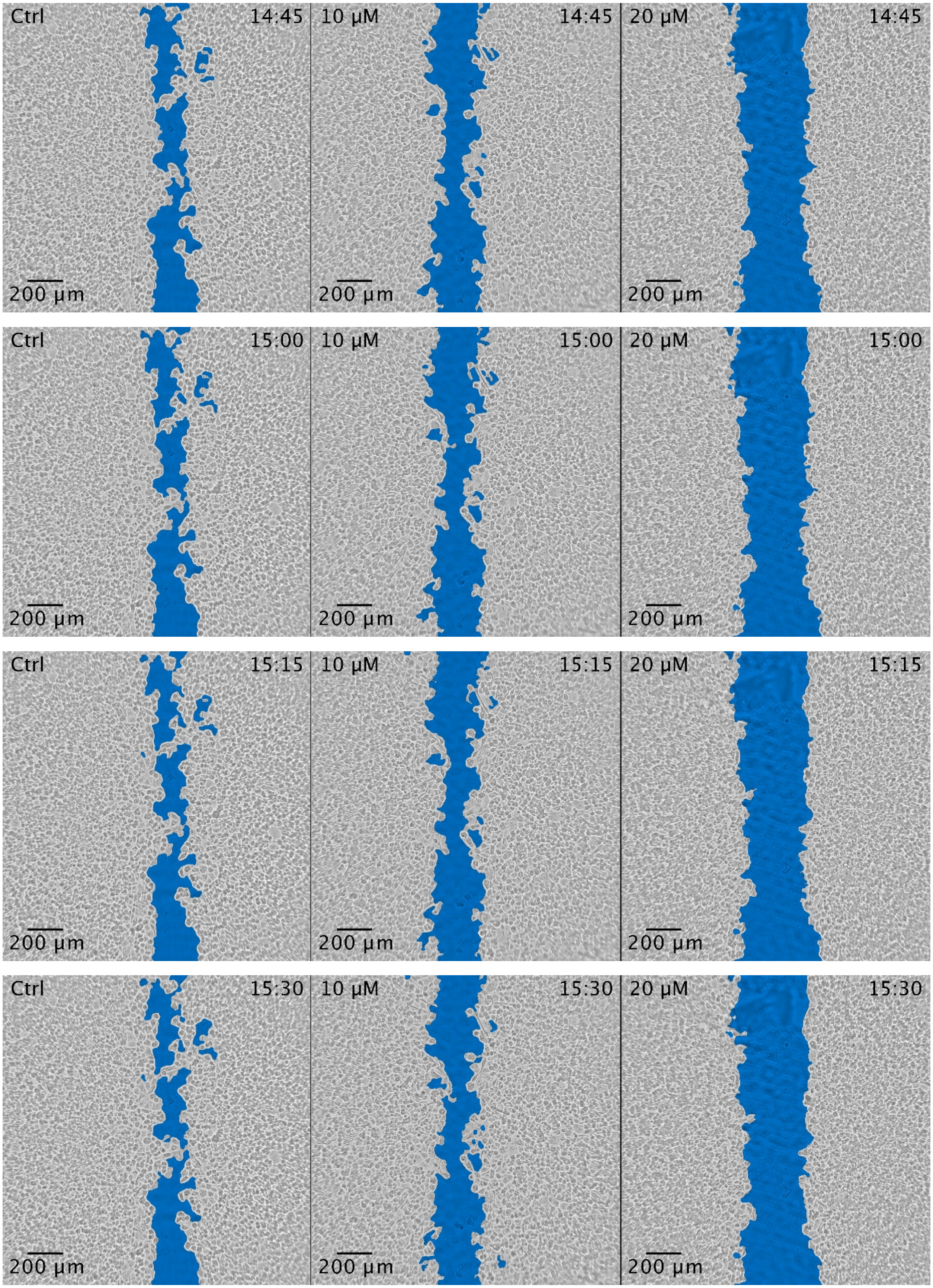
Ctrl
14:45
10 μM
14:45
20 μM
14:45
200 μm
15:00
15:15
15:30

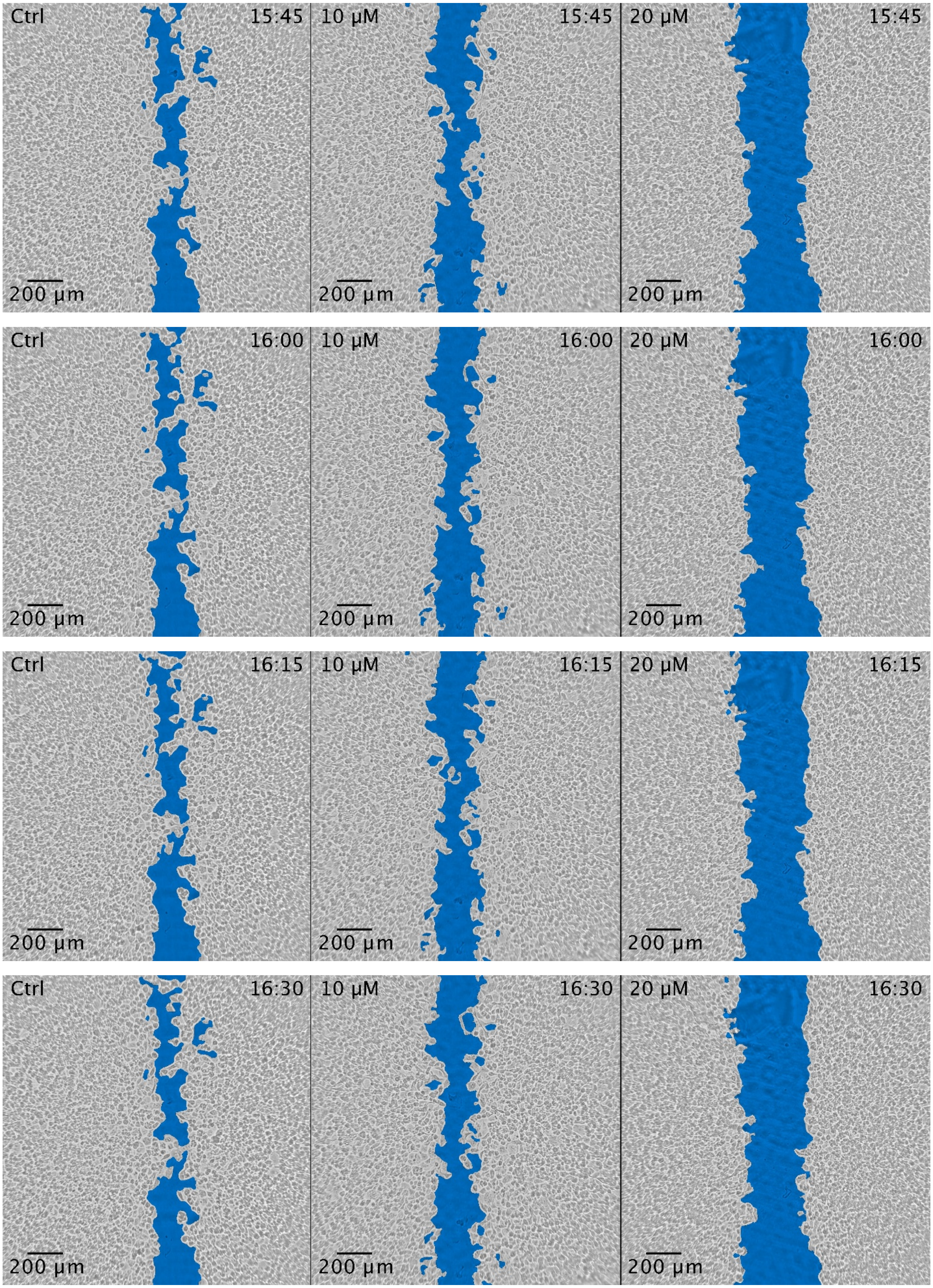
Ctrl
15:45
10 µM
15:45
20 µM
15:45
200 µm
Ctrl
16:00
10 µM
16:00
20 µM
16:00
200 µm
Ctrl
16:15
10 µM
16:15
20 µM
16:15
200 µm
Ctrl
16:30
10 µM
16:30
20 µM
16:30
200 µm

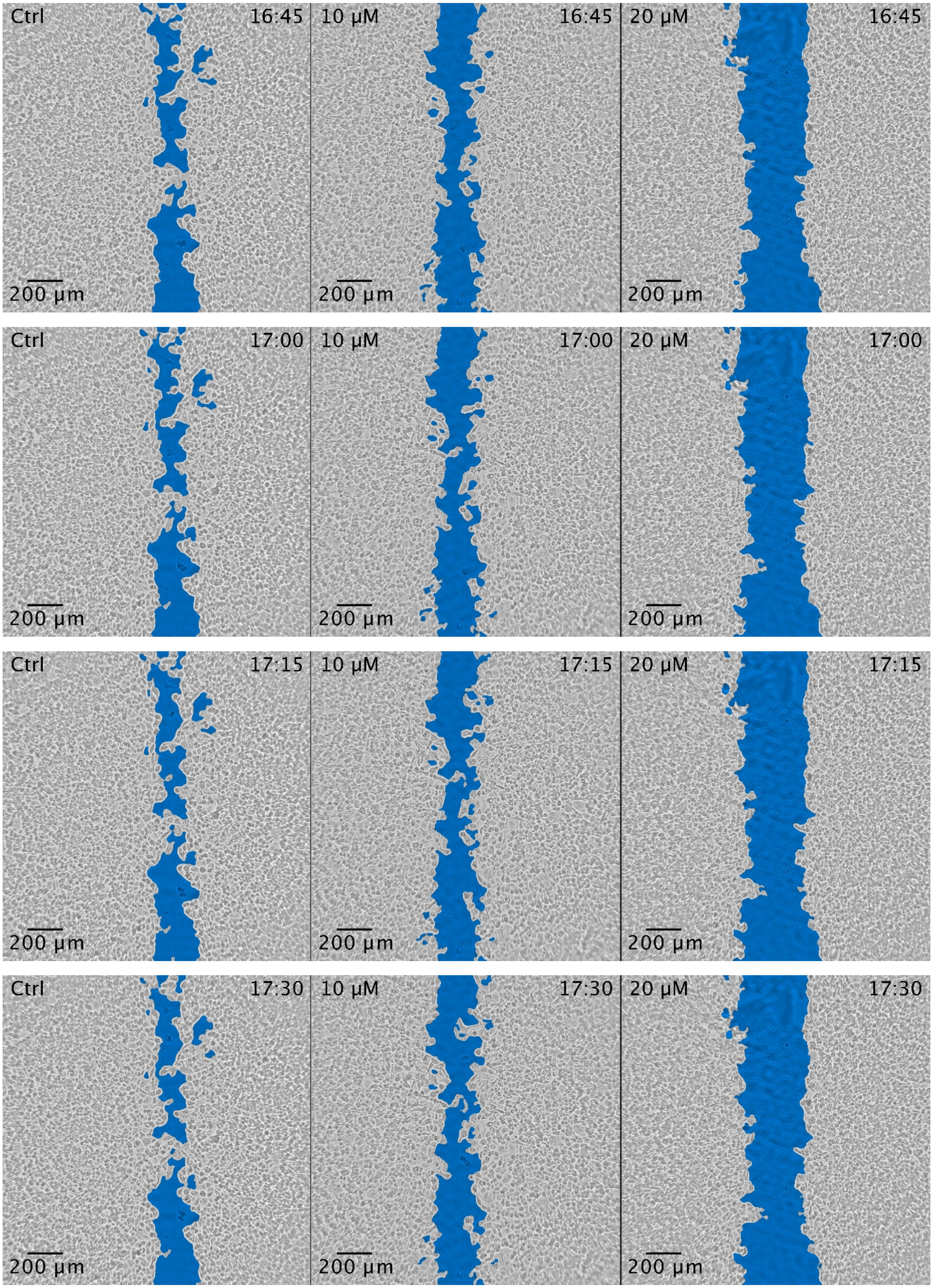
Ctrl
16:45
10 μM
16:45
20 μM
16:45
200 μm
Ctrl
17:00
10 μM
17:00
20 μM
17:00
200 μm
Ctrl
17:15
10 μM
17:15
20 μM
17:15
200 μm
Ctrl
17:30
10 μM
17:30
20 μM
17:30
200 μm

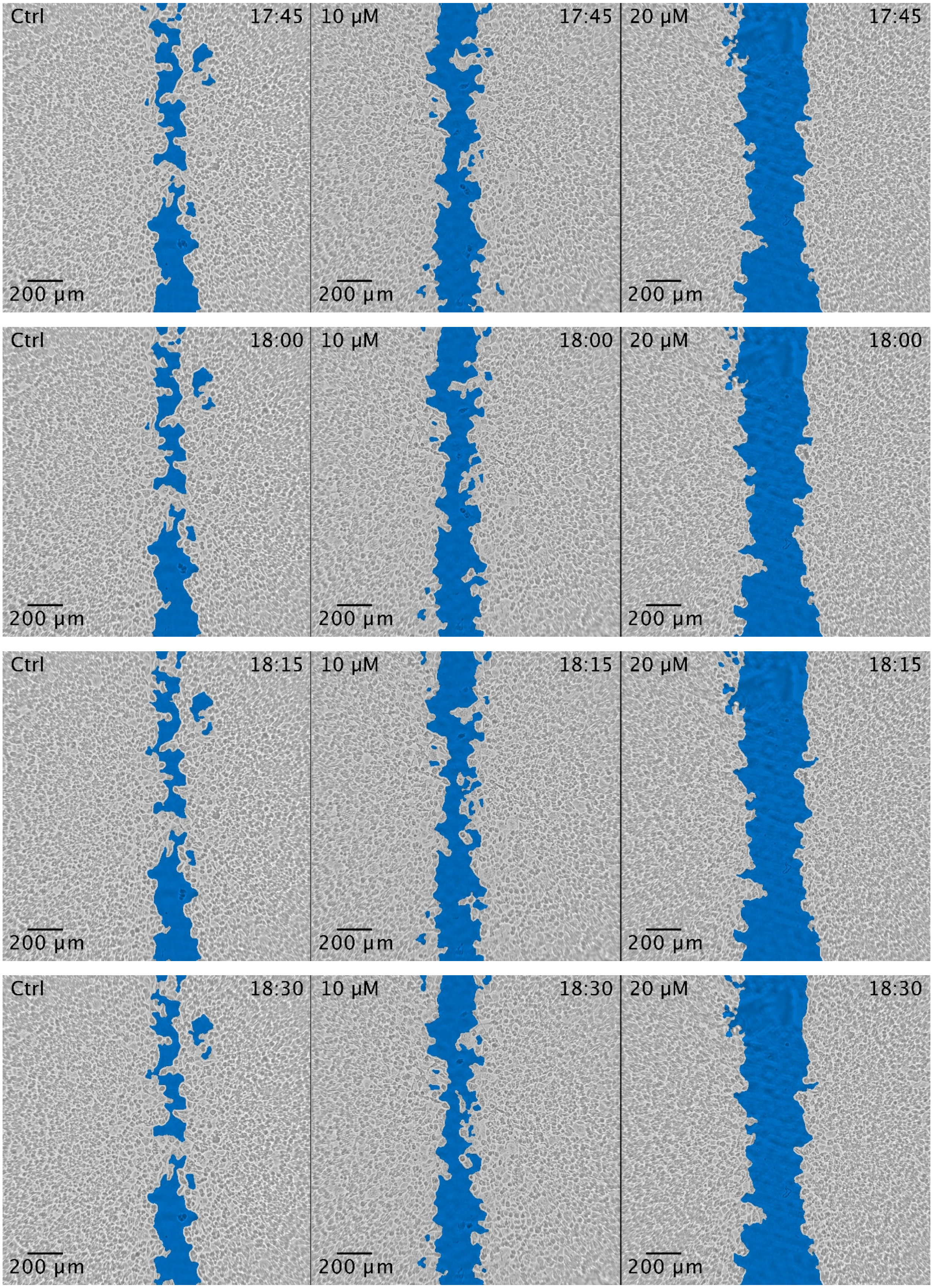
Ctrl
17:45
10 µM
17:45
20 µM
17:45
200 µm
200 µm
200 µm
Ctrl
18:00
10 µM
18:00
20 µM
18:00
200 µm
200 µm
200 µm
Ctrl
18:15
10 µM
18:15
20 µM
18:15
200 µm
200 µm
200 µm
Ctrl
18:30
10 µM
18:30
20 µM
18:30
200 µm
200 µm
200 µm

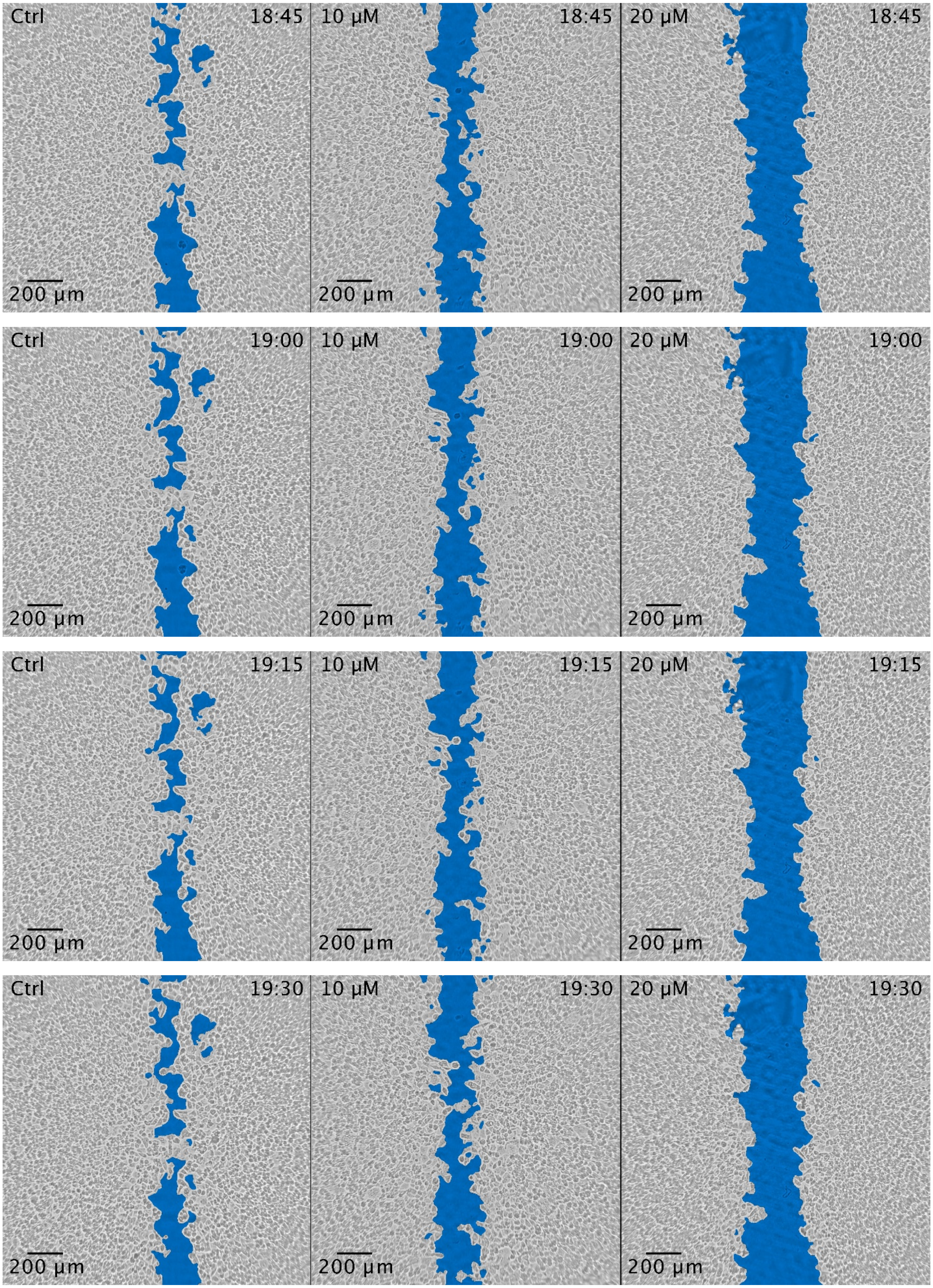
Ctrl
18:45
10 μM
18:45
20 μM
18:45
200 μm
19:00
19:15
19:30

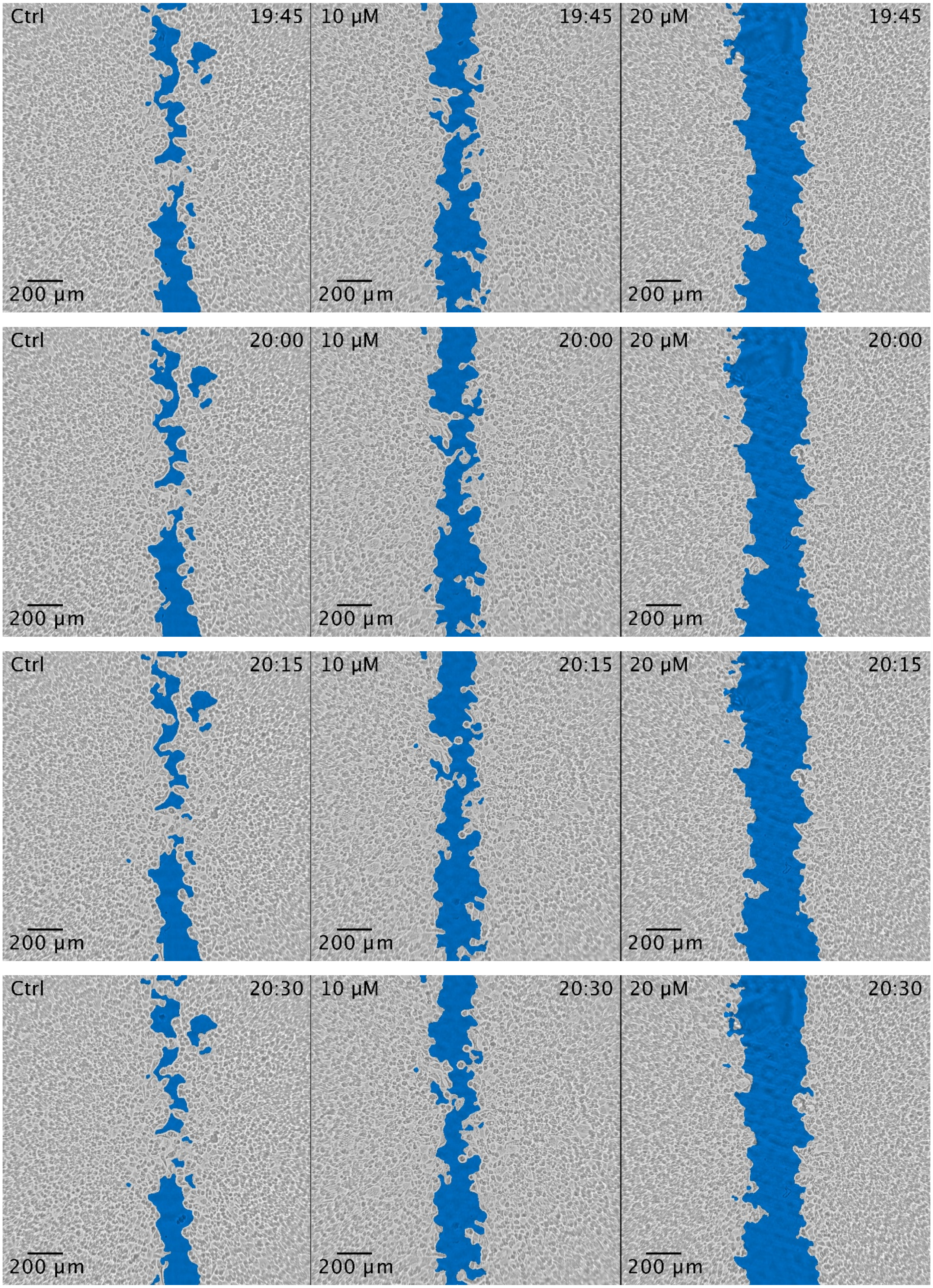
Ctrl
19:45
10 µM
19:45
20 µM
19:45
200 µm
Ctrl
20:00
10 µM
20:00
20 µM
20:00
200 µm
Ctrl
20:15
10 µM
20:15
20 µM
20:15
200 µm
Ctrl
20:30
10 µM
20:30
20 µM
20:30
200 µm

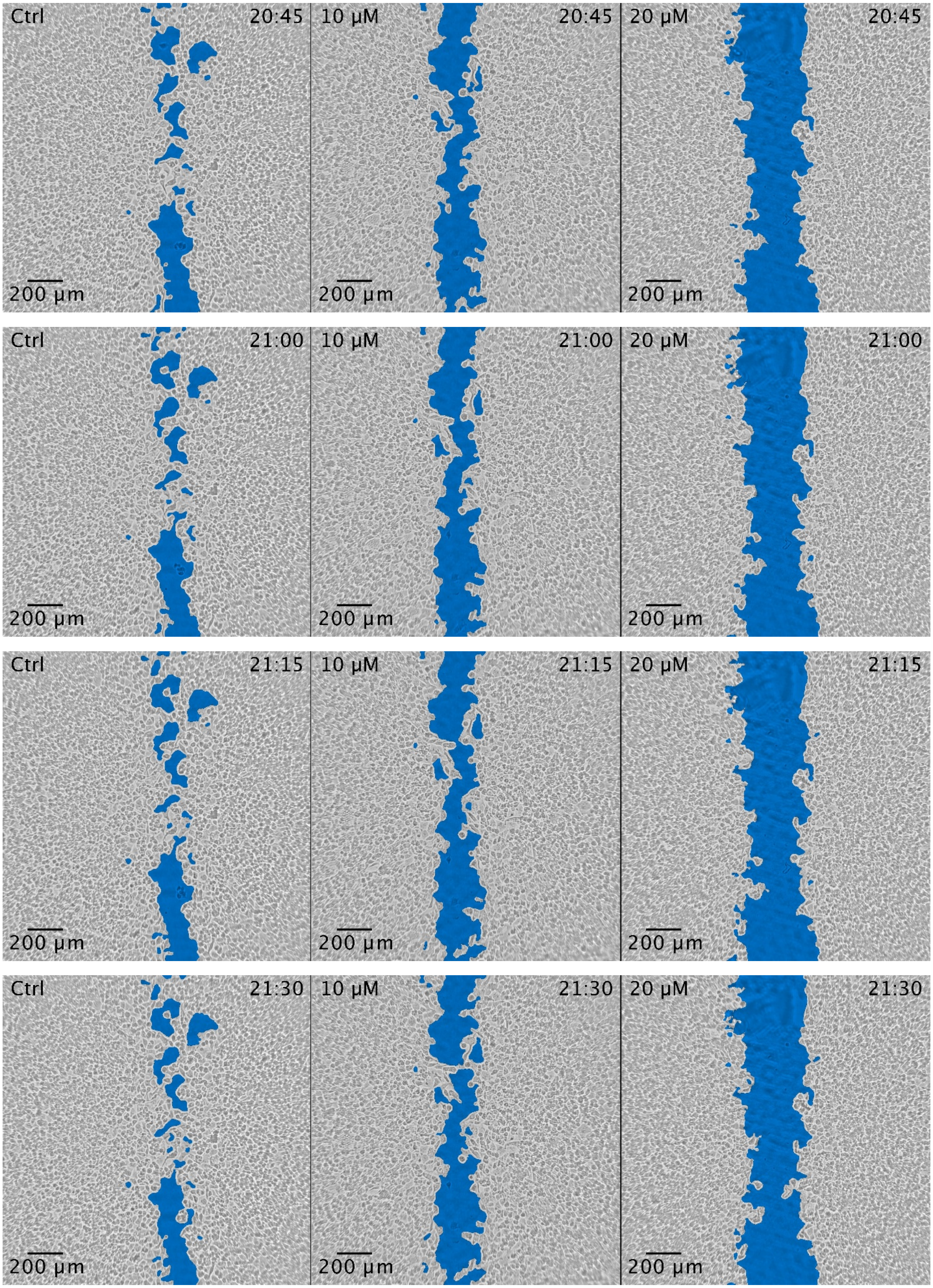
Ctrl
20:45
10 µM
20:45
20 µM
20:45
200 µm
21:00
21:15
21:30

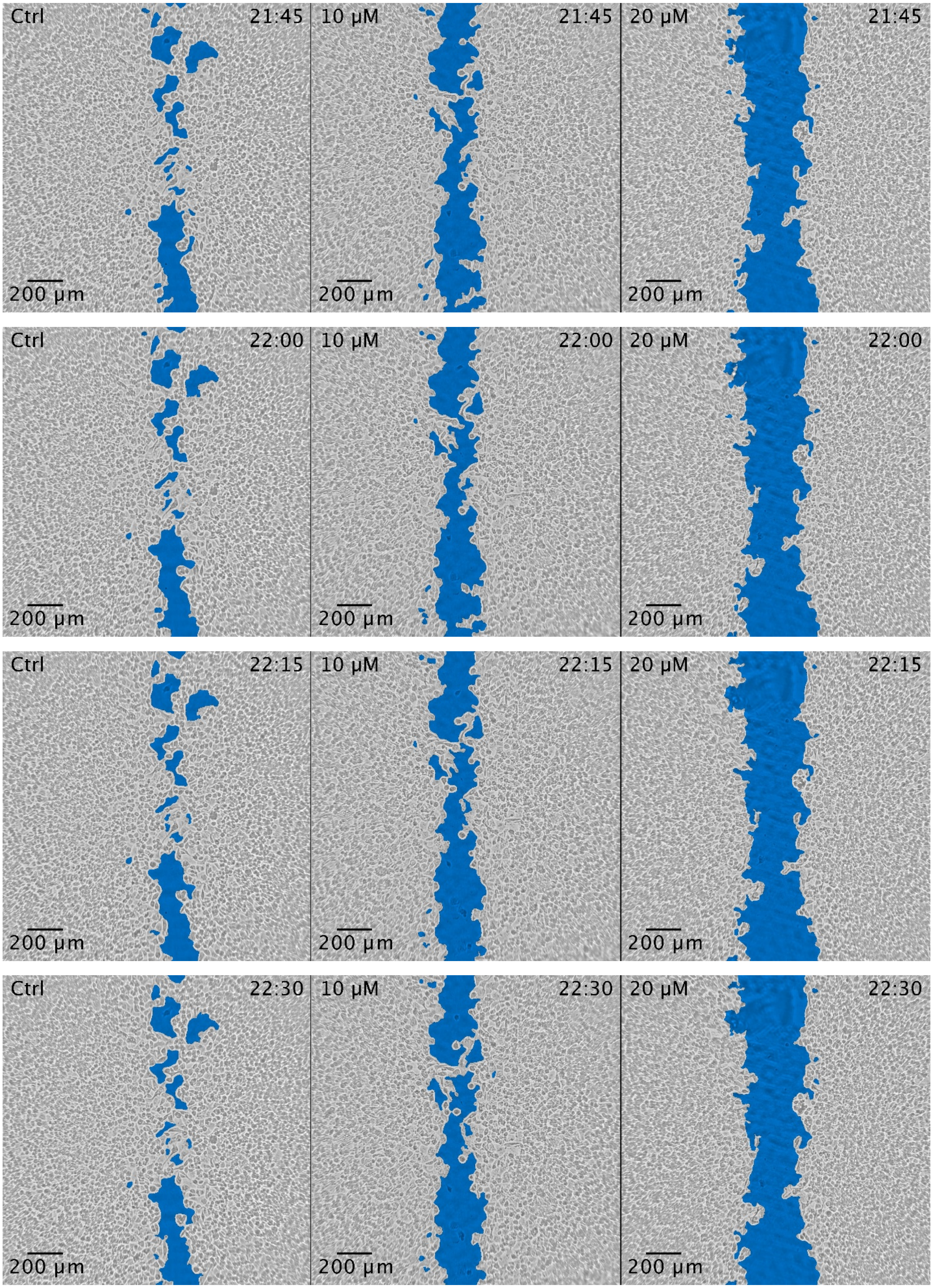

Ctrl
21:45
200 µm
10 µM
21:45
200 µm
20 µM
21:45
200 µm
Ctrl
22:00
200 µm
10 µM
22:00
200 µm
20 µM
22:00
200 µm
Ctrl
22:15
200 µm
10 µM
22:15
200 µm
20 µM
22:15
200 µm
Ctrl
22:30
200 µm
10 µM
22:30
200 µm
20 µM
22:30
200 µm

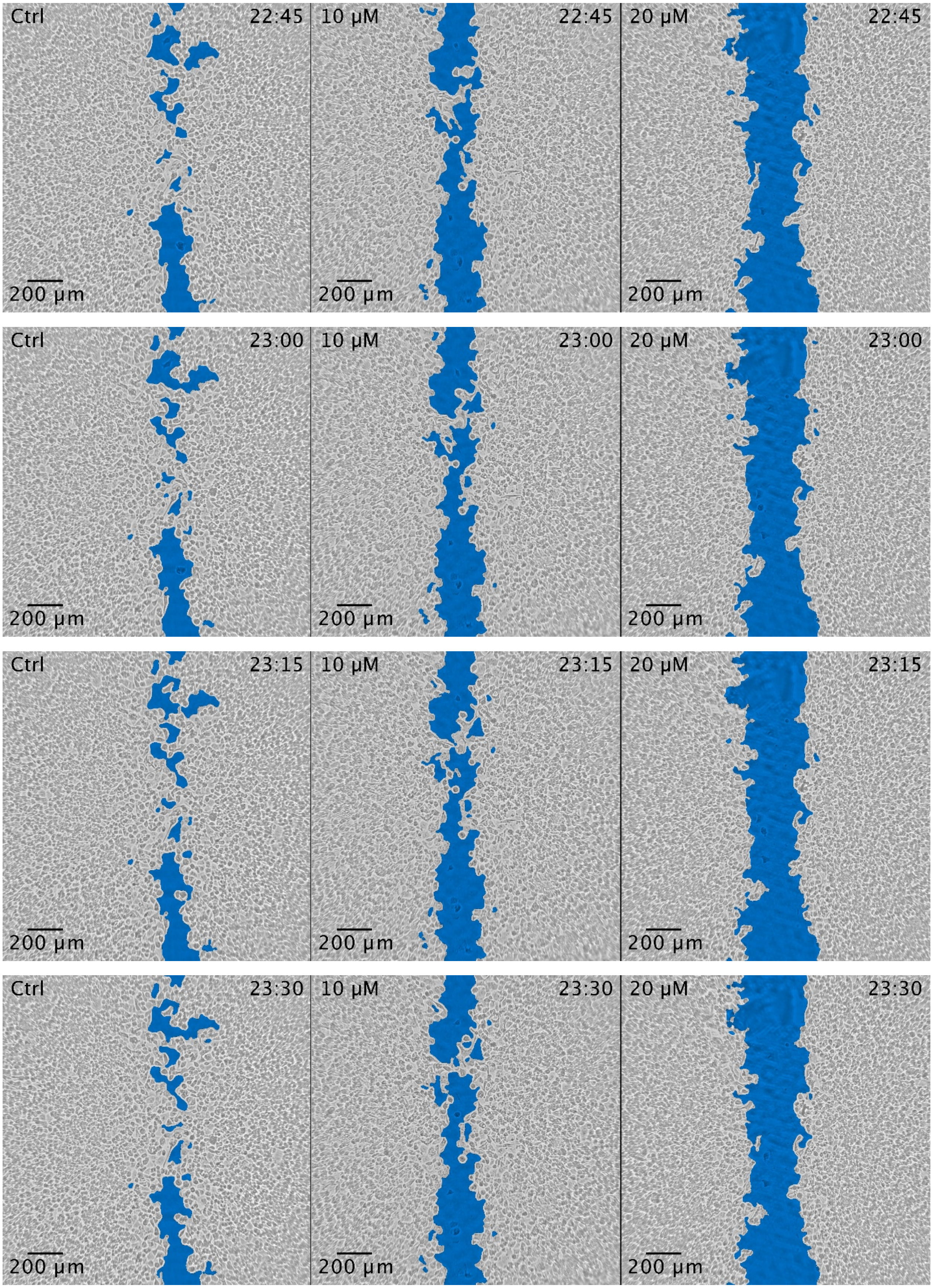
Ctrl
22:45
10 μM
22:45
20 μM
22:45
200 μm
200 μm
200 μm
Ctrl
23:00
10 μM
23:00
20 μM
23:00
200 μm
200 μm
200 μm
Ctrl
23:15
10 μM
23:15
20 μM
23:15
200 μm
200 μm
200 μm
Ctrl
23:30
10 μM
23:30
20 μM
23:30
200 μm
200 μm
200 μm

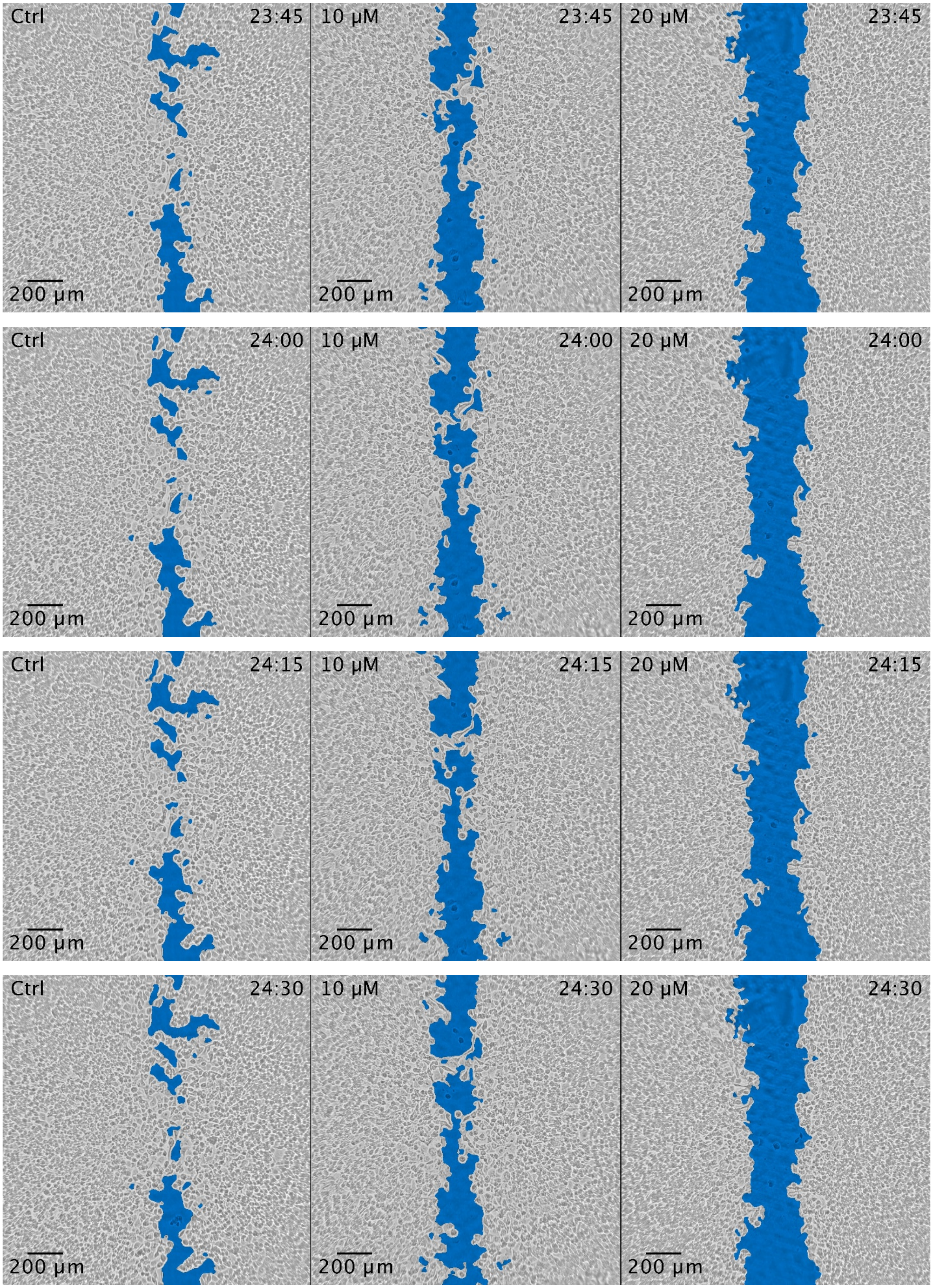
Ctrl
23:45
10 µM
23:45
20 µM
23:45
200 µm
Ctrl
24:00
10 µM
24:00
20 µM
24:00
200 µm
Ctrl
24:15
10 µM
24:15
20 µM
24:15
200 µm
Ctrl
24:30
10 µM
24:30
20 µM
24:30
200 µm

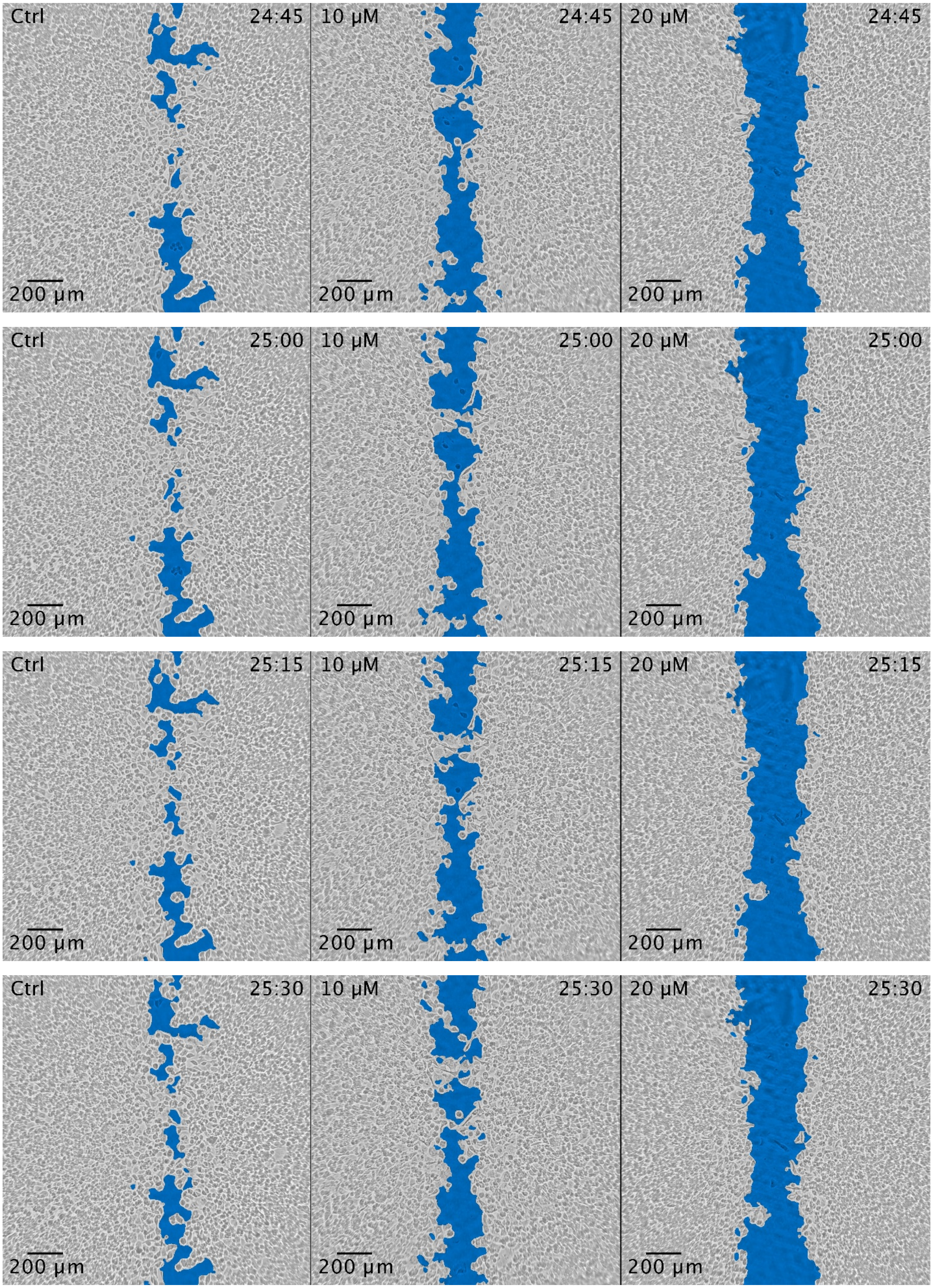
Ctrl
24:45
10 μM
24:45
20 μM
24:45
200 μm
200 μm
200 μm
Ctrl
25:00
10 μM
25:00
20 μM
25:00
200 μm
200 μm
200 μm
Ctrl
25:15
10 μM
25:15
20 μM
25:15
200 μm
200 μm
200 μm
Ctrl
25:30
10 μM
25:30
20 μM
25:30
200 μm
200 μm
200 μm

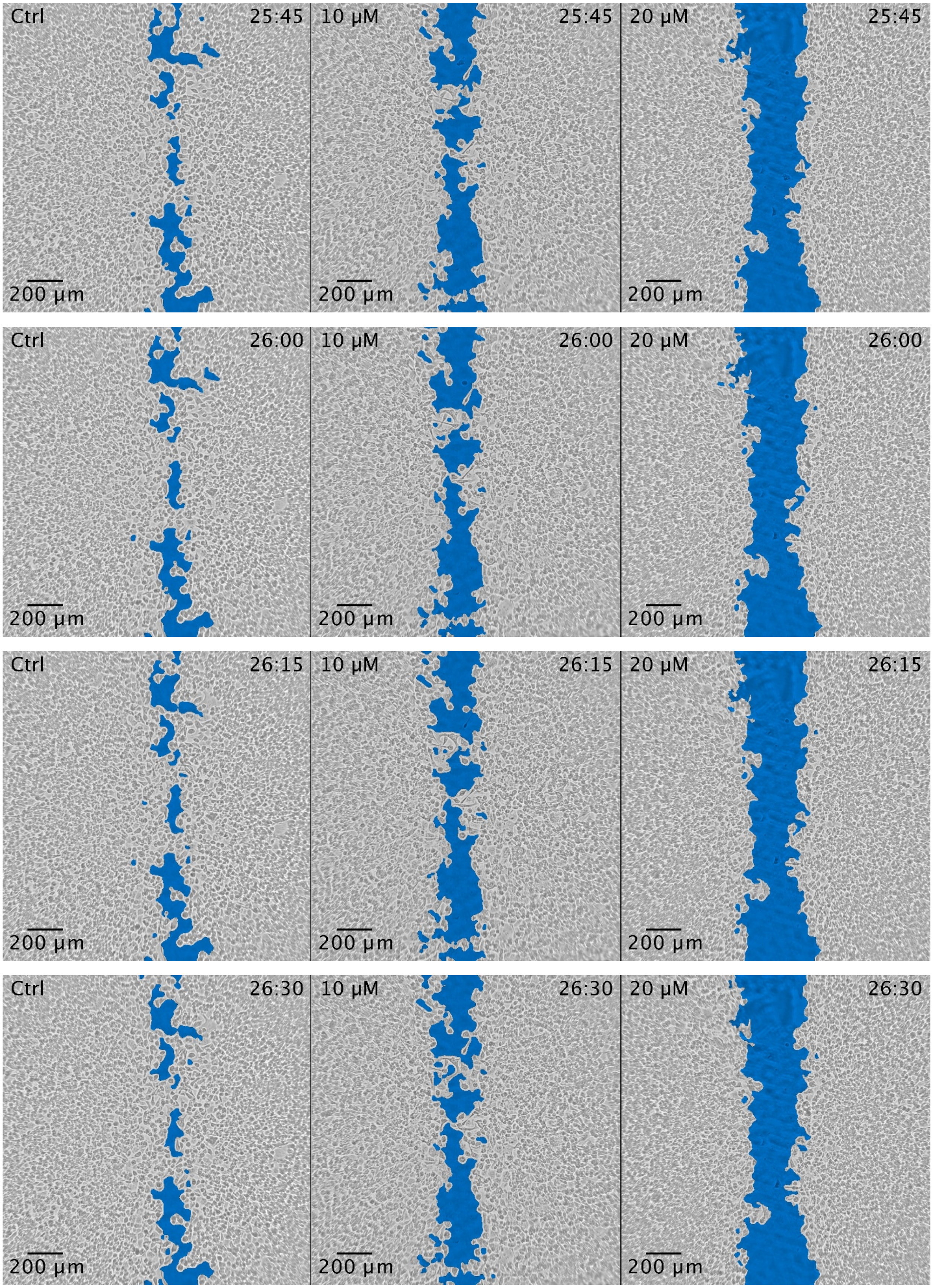
Ctrl
25:45
10 μM
25:45
20 μM
25:45
200 μm
200 μm
200 μm
Ctrl
26:00
10 μM
26:00
20 μM
26:00
200 μm
200 μm
200 μm
Ctrl
26:15
10 μM
26:15
20 μM
26:15
200 μm
200 μm
200 μm
Ctrl
26:30
10 μM
26:30
20 μM
26:30
200 μm
200 μm
200 μm

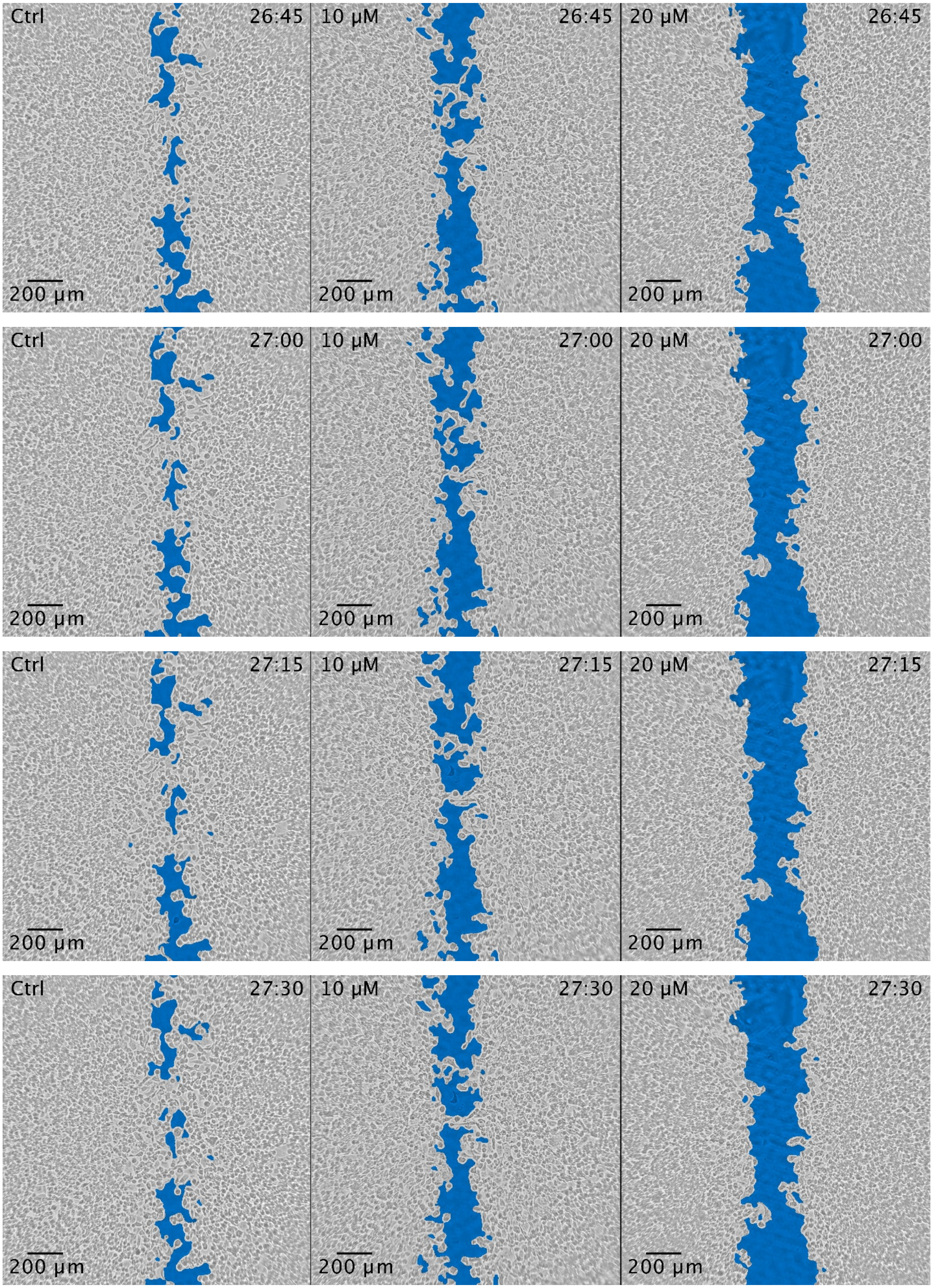
Ctrl
26:45
10 μM
26:45
20 μM
26:45
200 μm
Ctrl
27:00
10 μM
27:00
20 μM
27:00
200 μm
Ctrl
27:15
10 μM
27:15
20 μM
27:15
200 μm
Ctrl
27:30
10 μM
27:30
20 μM
27:30
200 μm

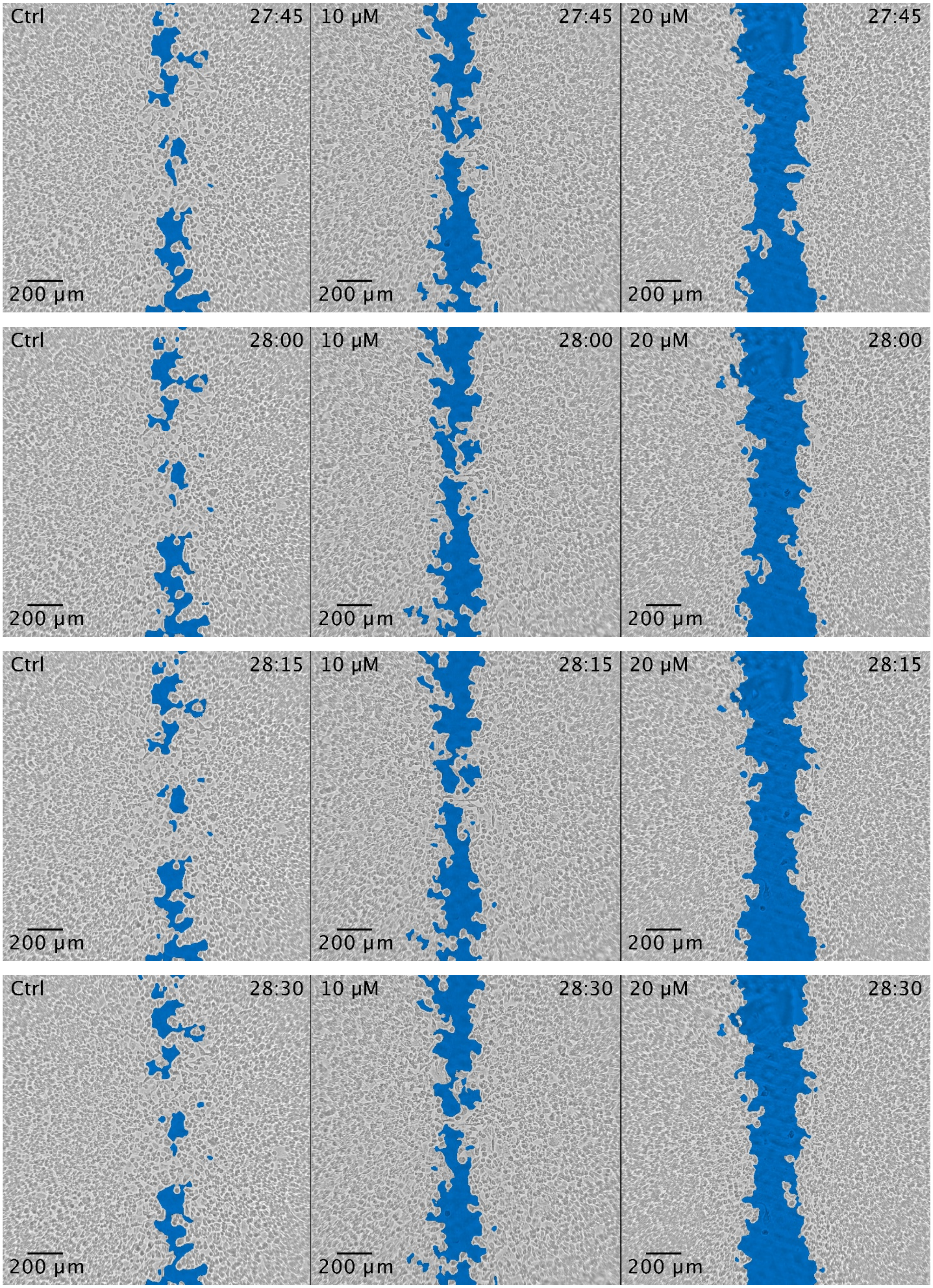
Ctrl
27:45
10 µM
27:45
20 µM
27:45
200 µm
Ctrl
28:00
10 µM
28:00
20 µM
28:00
200 µm
Ctrl
28:15
10 µM
28:15
20 µM
28:15
200 µm
Ctrl
28:30
10 µM
28:30
20 µM
28:30
200 µm

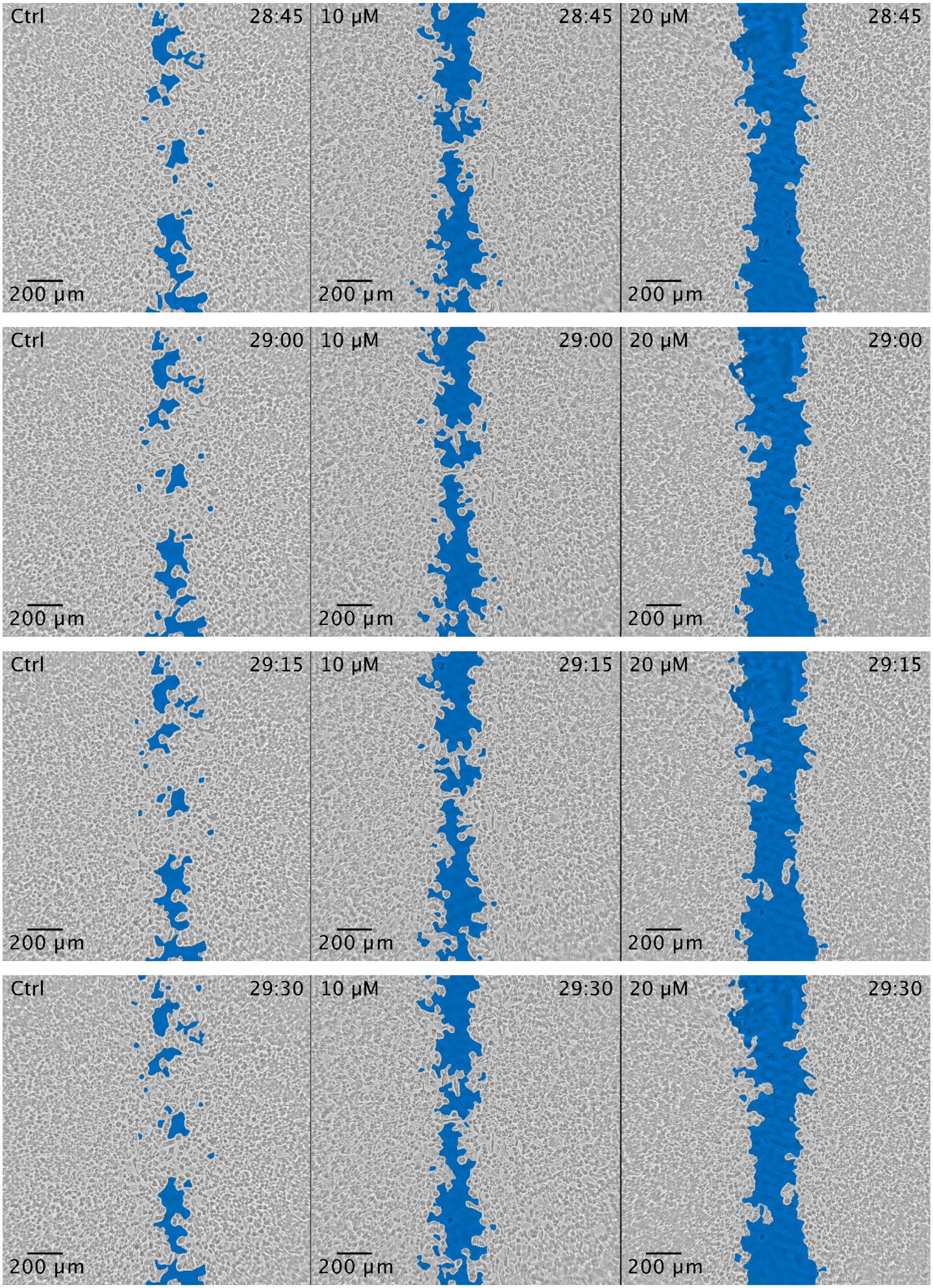
Ctrl
28:45
10 μM
28:45
20 μM
28:45
200 μm
200 μm
200 μm
Ctrl
29:00
10 μM
29:00
20 μM
29:00
200 μm
200 μm
200 μm
Ctrl
29:15
10 μM
29:15
20 μM
29:15
200 μm
200 μm
200 μm
Ctrl
29:30
10 μM
29:30
20 μM
29:30
200 μm
200 μm
200 μm

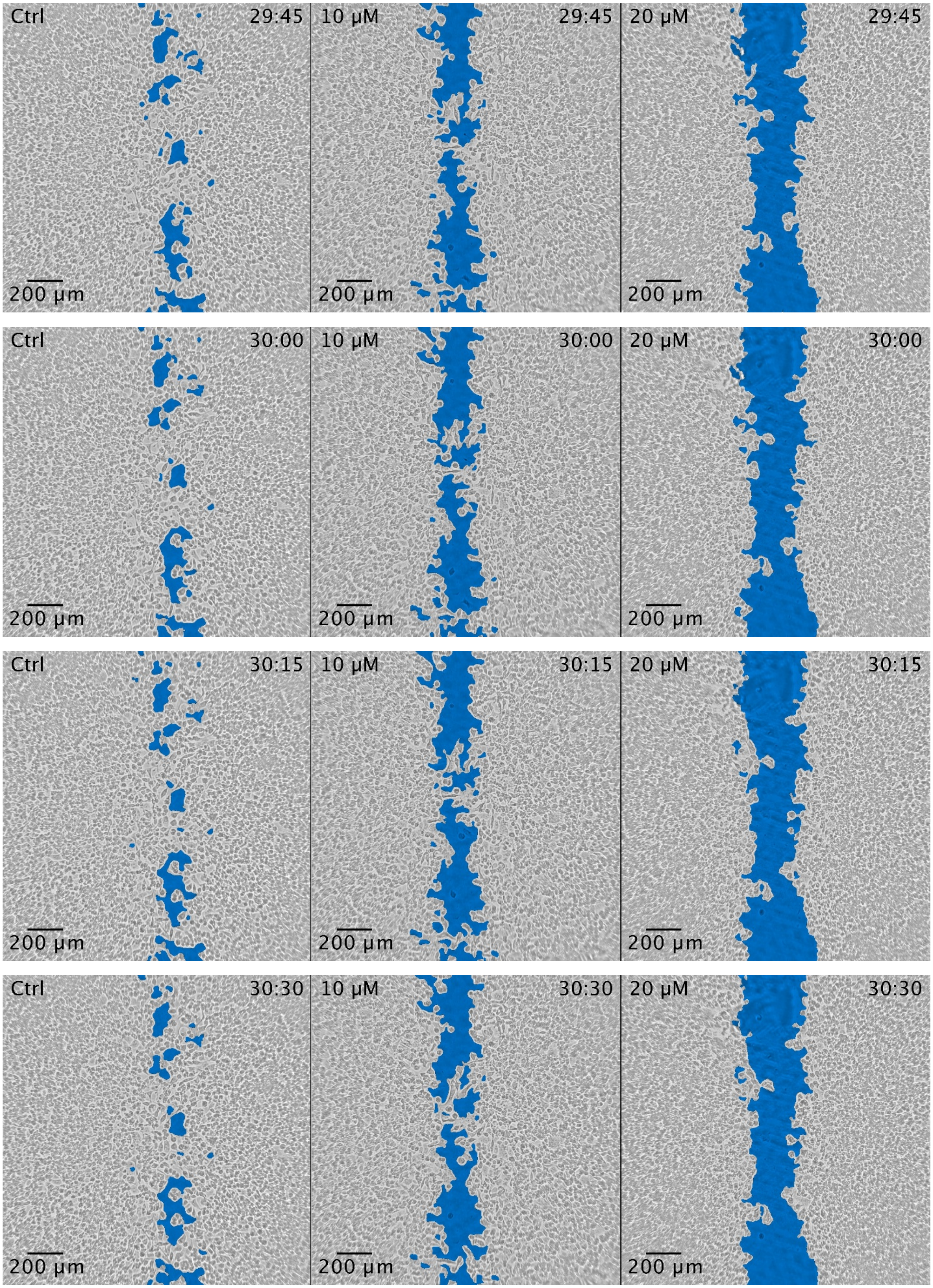
Ctrl
29:45
10 μM
29:45
20 μM
29:45
200 μm
200 μm
200 μm
Ctrl
30:00
10 μM
30:00
20 μM
30:00
200 μm
200 μm
200 μm
Ctrl
30:15
10 μM
30:15
20 μM
30:15
200 μm
200 μm
200 μm
Ctrl
30:30
10 μM
30:30
20 μM
30:30
200 μm
200 μm
200 μm

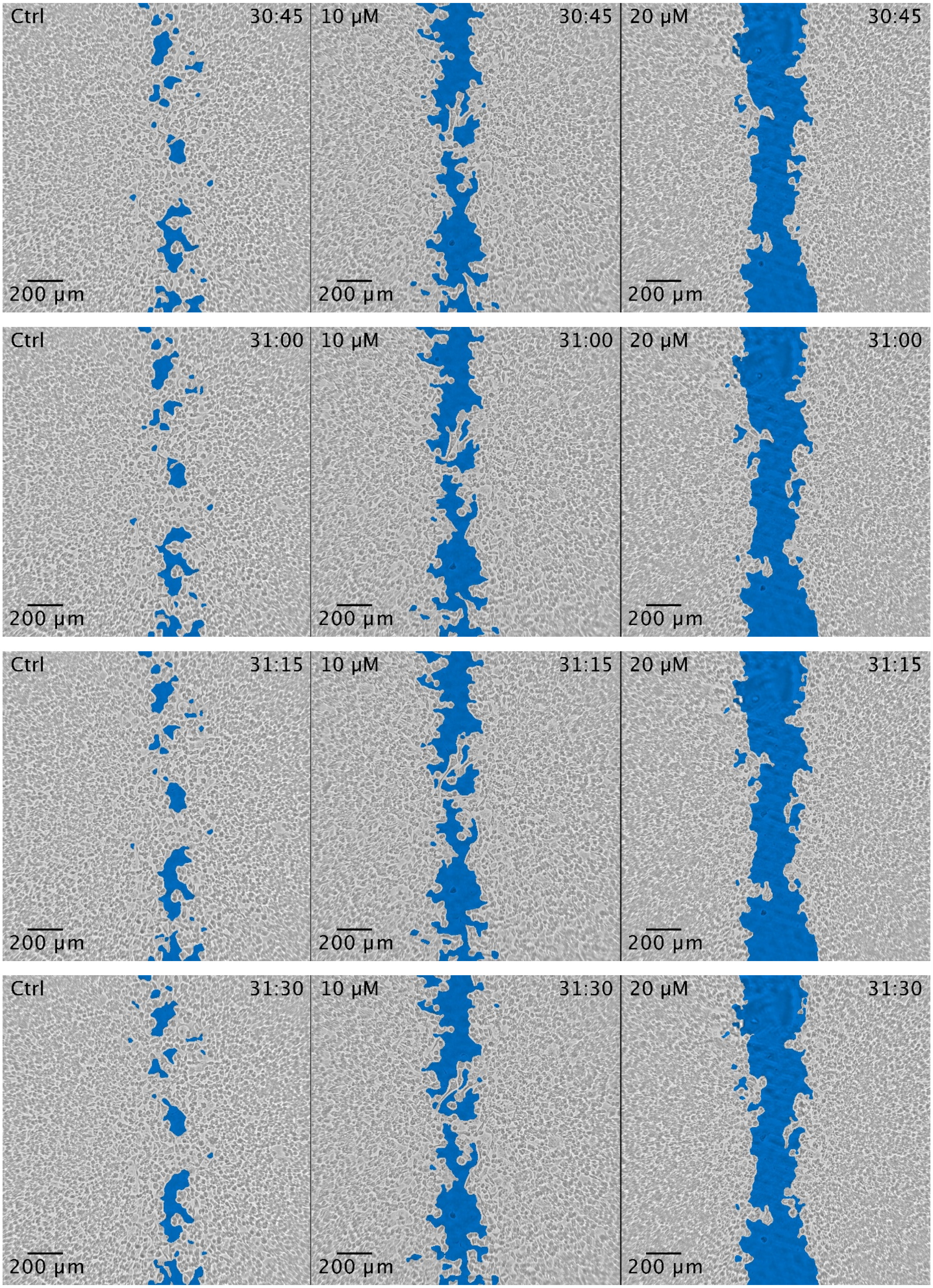
Ctrl
30:45
10 μM
30:45
20 μM
30:45
200 μm
Ctrl
31:00
10 μM
31:00
20 μM
31:00
Ctrl
31:15
10 μM
31:15
20 μM
31:15
Ctrl
31:30
10 μM
31:30
20 μM
31:30

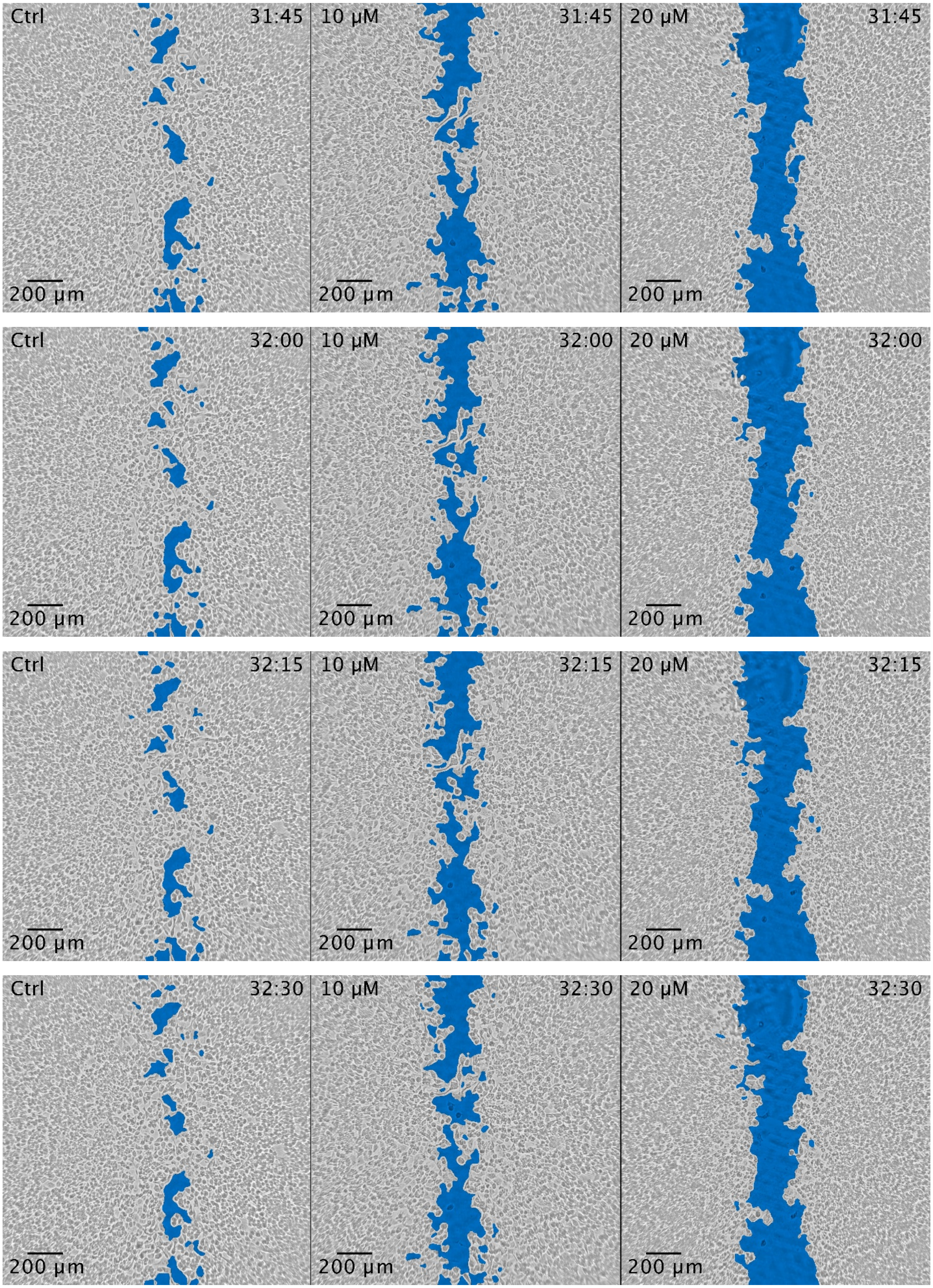
Ctrl
31:45
10 µM
31:45
20 µM
31:45
200 µm
200 µm
200 µm
Ctrl
32:00
10 µM
32:00
20 µM
32:00
200 µm
200 µm
200 µm
Ctrl
32:15
10 µM
32:15
20 µM
32:15
200 µm
200 µm
200 µm
Ctrl
32:30
10 µM
32:30
20 µM
32:30
200 µm
200 µm
200 µm

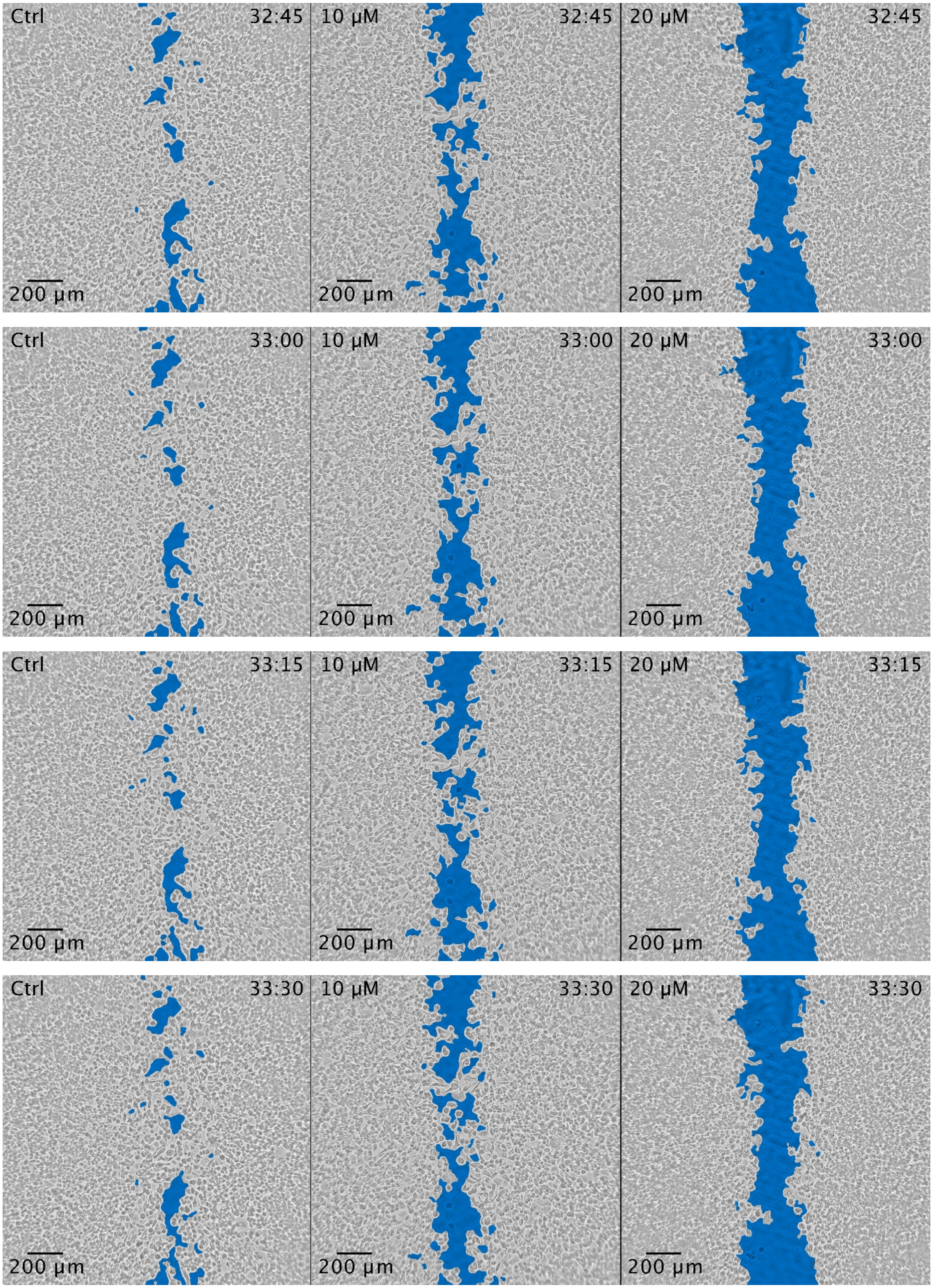
Ctrl
32:45
10 µM
32:45
20 µM
32:45
200 µm
Ctrl
33:00
10 µM
33:00
20 µM
33:00
Ctrl
33:15
10 µM
33:15
20 µM
33:15
Ctrl
33:30
10 µM
33:30
20 µM
33:30

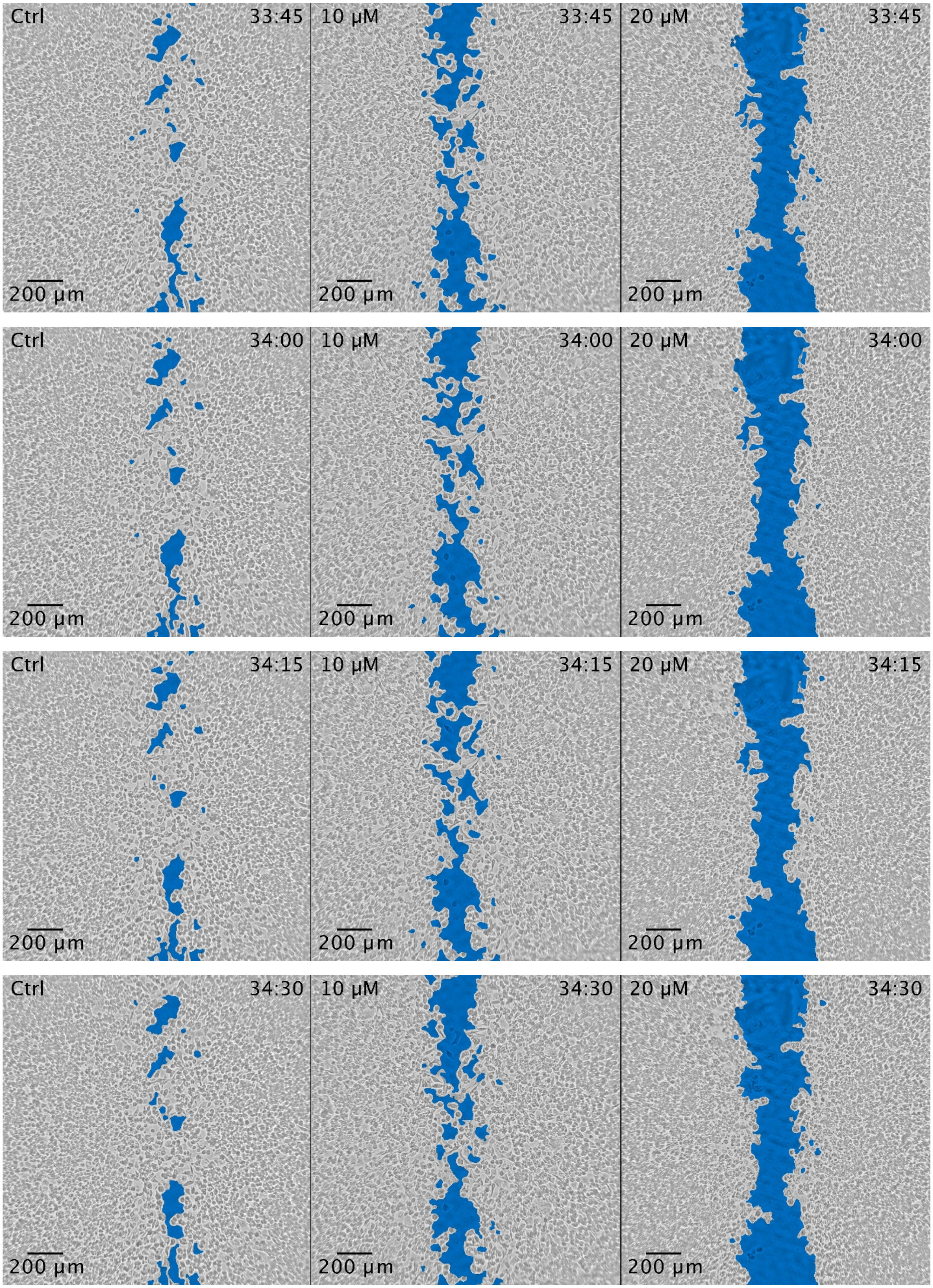

Ctrl
33:45
10 μM
33:45
20 μM
33:45
200 μm
200 μm
200 μm
Ctrl
34:00
10 μM
34:00
20 μM
34:00
200 μm
200 μm
200 μm
Ctrl
34:15
10 μM
34:15
20 μM
34:15
200 μm
200 μm
200 μm
Ctrl
34:30
10 μM
34:30
20 μM
34:30
200 μm
200 μm
200 μm

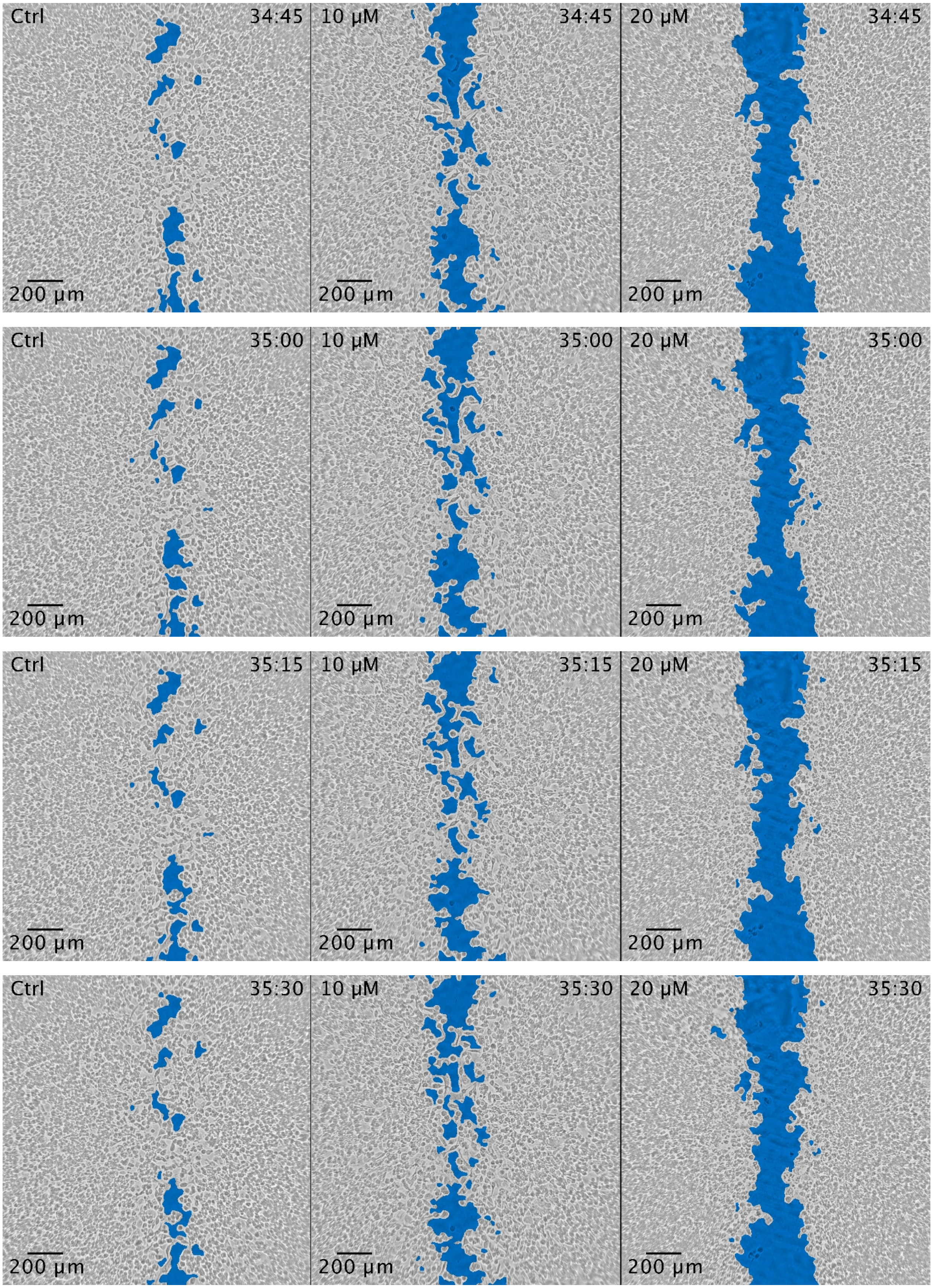

Ctrl
34:45
10 μM
34:45
20 μM
34:45
200 μm
Ctrl
35:00
10 μM
35:00
20 μM
35:00
200 μm
Ctrl
35:15
10 μM
35:15
20 μM
35:15
200 μm
Ctrl
35:30
10 μM
35:30
20 μM
35:30
200 μm

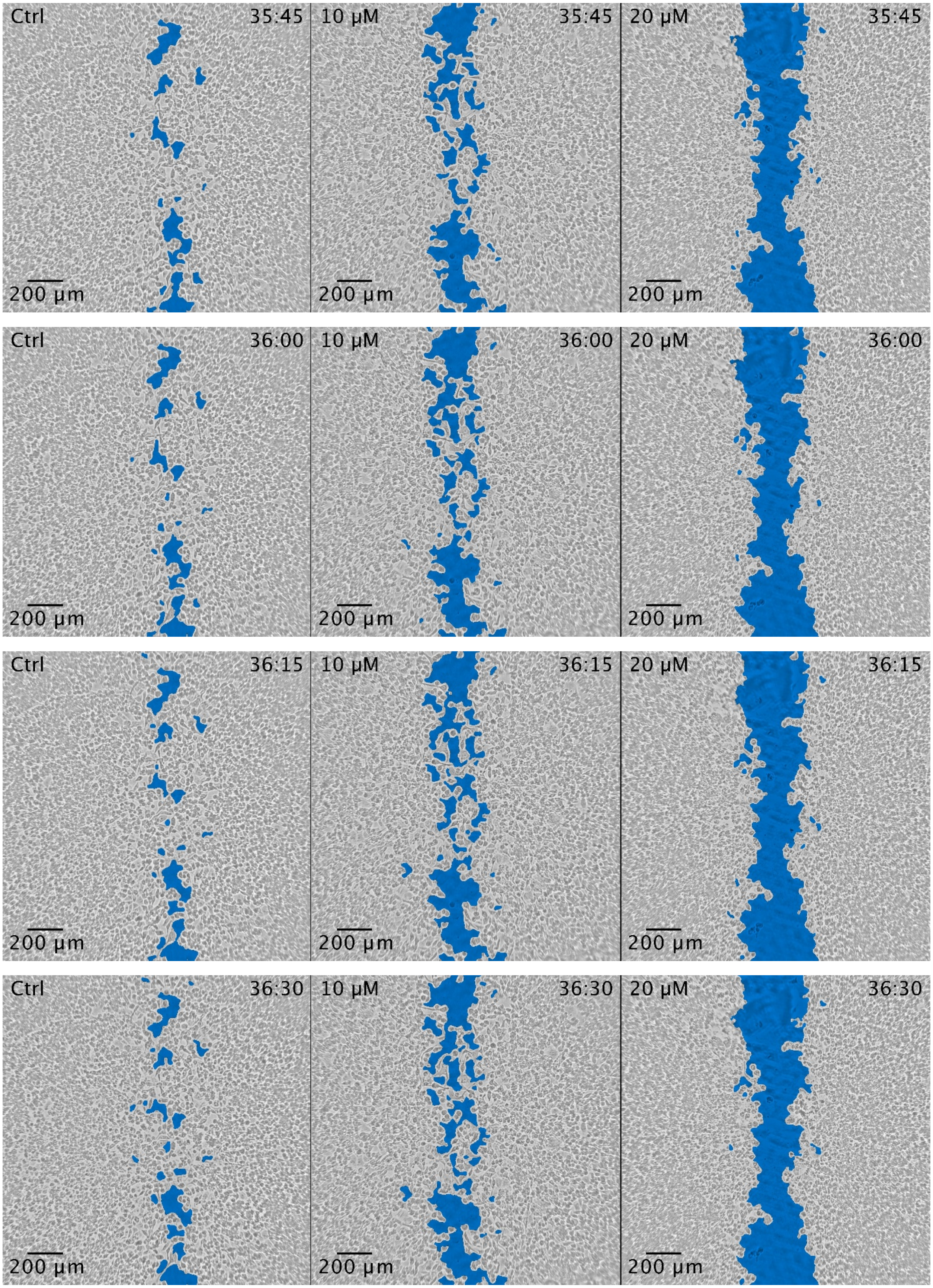
Ctrl
35:45
10 μM
35:45
20 μM
35:45
200 μm
Ctrl
36:00
10 μM
36:00
20 μM
36:00
200 μm
Ctrl
36:15
10 μM
36:15
20 μM
36:15
200 μm
Ctrl
36:30
10 μM
36:30
20 μM
36:30
200 μm

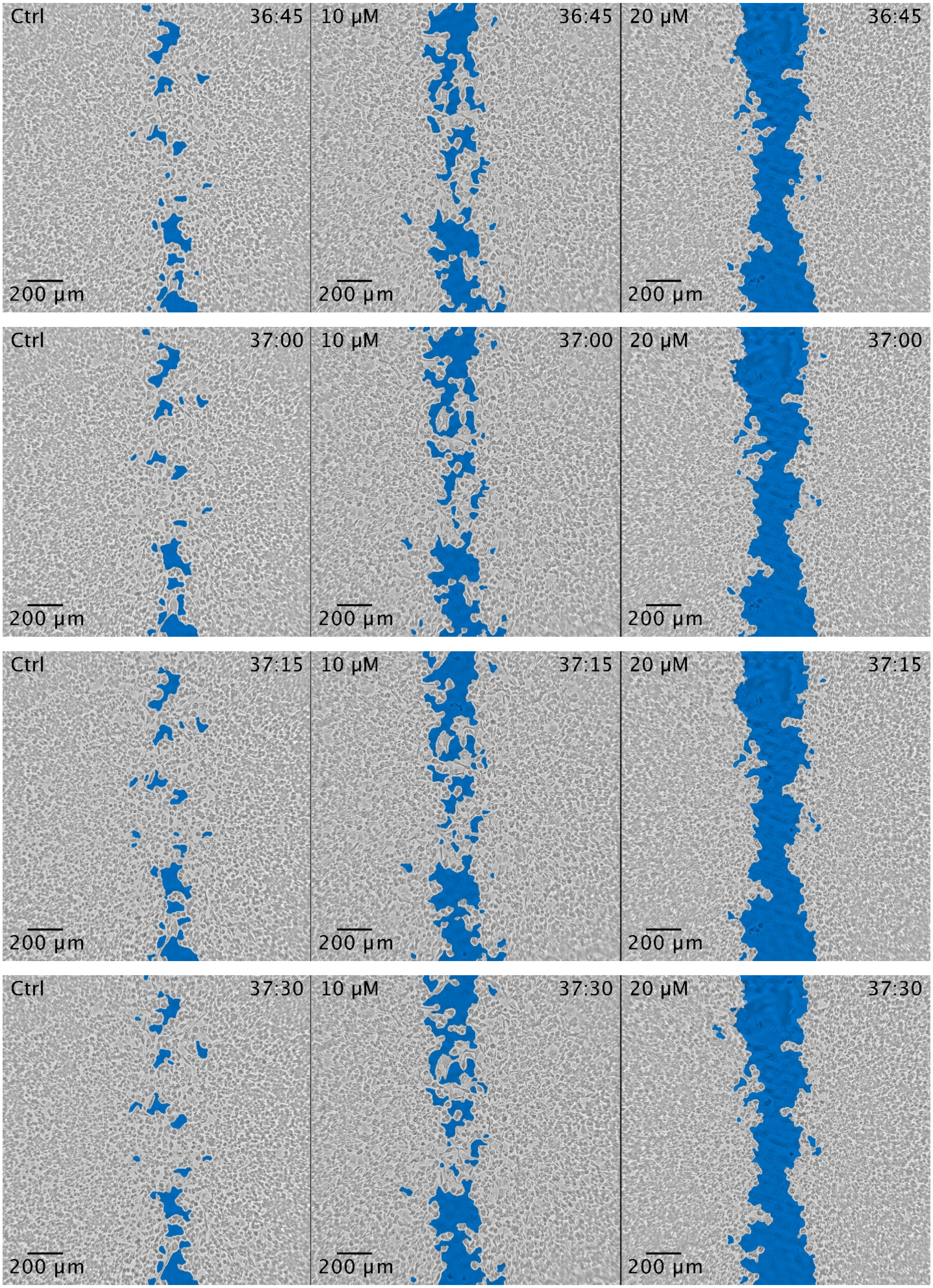
Ctrl
36:45
10 μM
36:45
20 μM
36:45
200 μm
Ctrl
37:00
10 μM
37:00
20 μM
37:00
200 μm
Ctrl
37:15
10 μM
37:15
20 μM
37:15
200 μm
Ctrl
37:30
10 μM
37:30
20 μM
37:30
200 μm

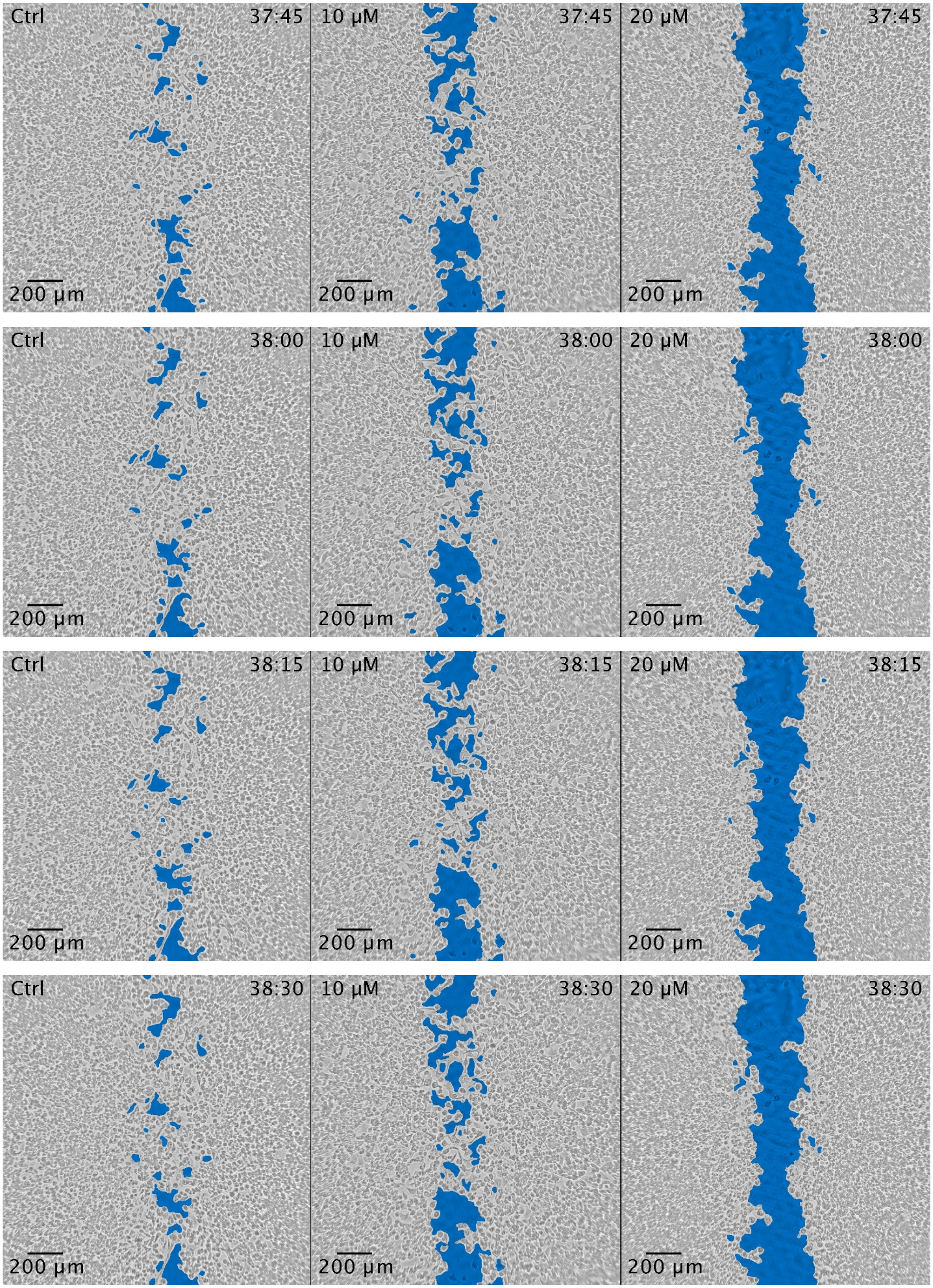
Ctrl
37:45
10 μM
37:45
20 μM
37:45
200 μm
Ctrl
38:00
10 μM
38:00
20 μM
38:00
200 μm
Ctrl
38:15
10 μM
38:15
20 μM
38:15
200 μm
Ctrl
38:30
10 μM
38:30
20 μM
38:30
200 μm

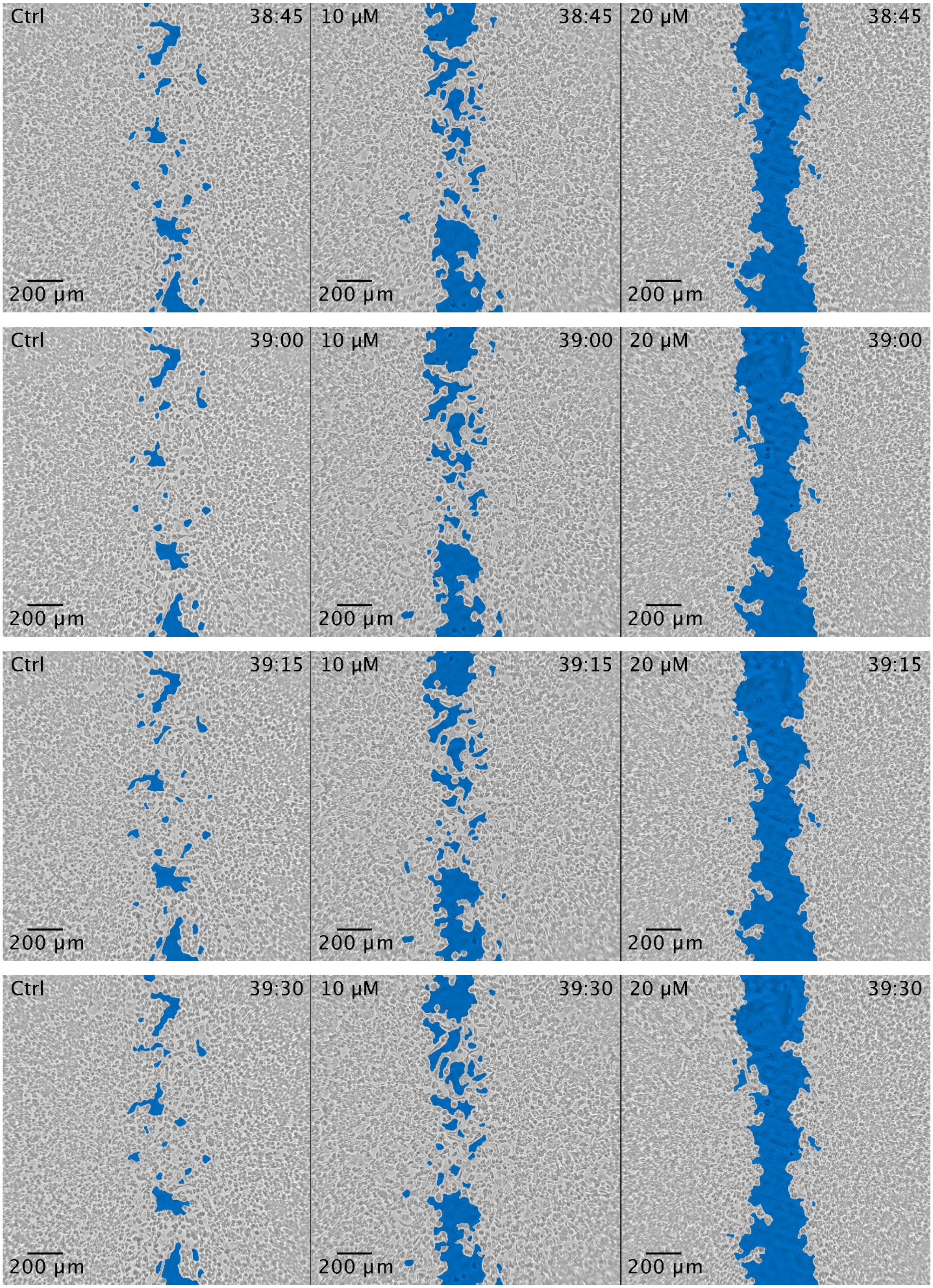
Ctrl
38:45
10 μM
38:45
20 μM
38:45
200 μm
200 μm
200 μm
Ctrl
39:00
10 μM
39:00
20 μM
39:00
200 μm
200 μm
200 μm
Ctrl
39:15
10 μM
39:15
20 μM
39:15
200 μm
200 μm
200 μm
Ctrl
39:30
10 μM
39:30
20 μM
39:30
200 μm
200 μm
200 μm

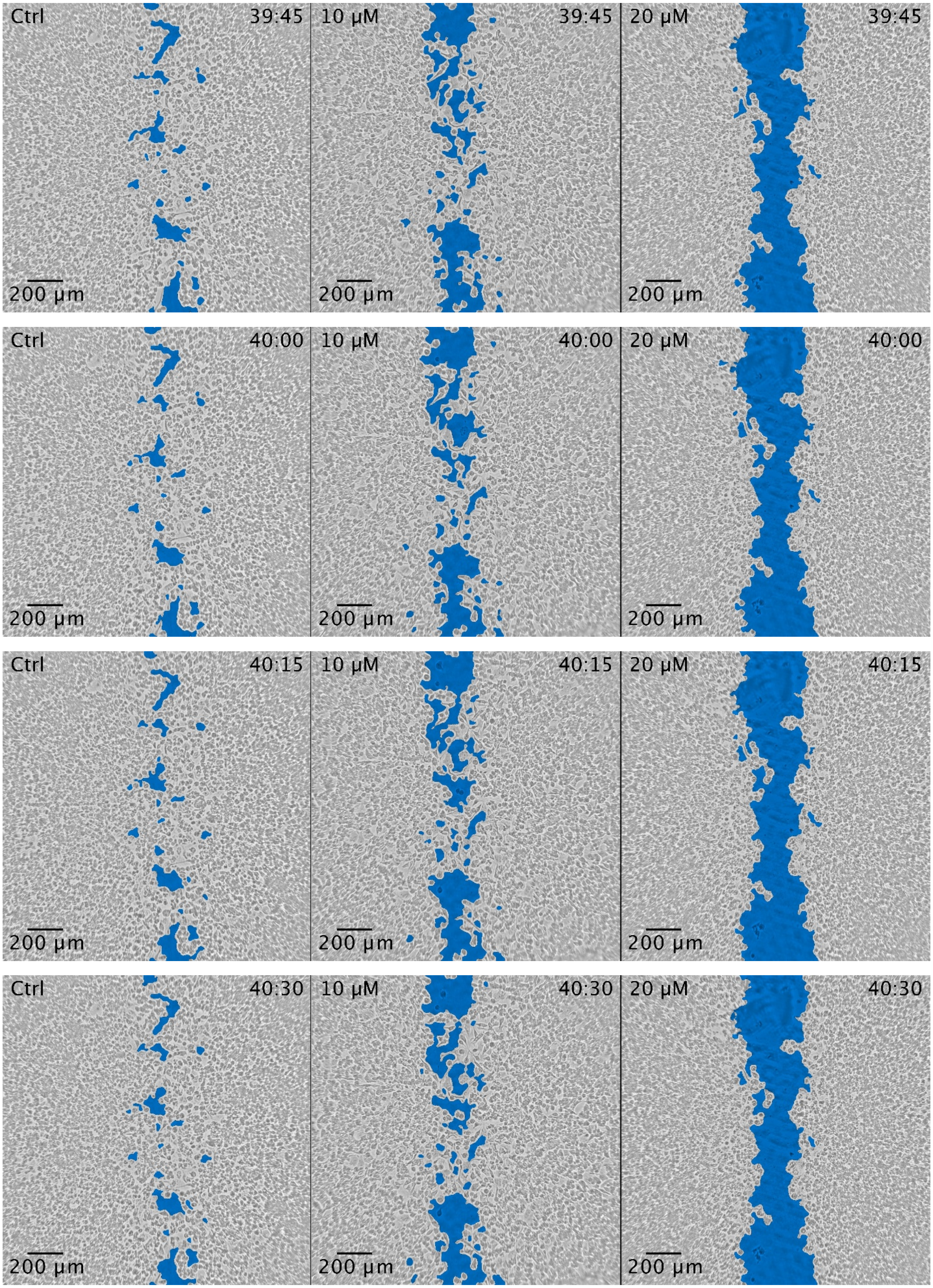

Ctrl
39:45
200 µm
10 µM
39:45
200 µm
20 µM
39:45
200 µm
Ctrl
40:00
200 µm
10 µM
40:00
200 µm
20 µM
40:00
200 µm
Ctrl
40:15
200 µm
10 µM
40:15
200 µm
20 µM
40:15
200 µm
Ctrl
40:30
200 µm
10 µM
40:30
200 µm
20 µM
40:30
200 µm

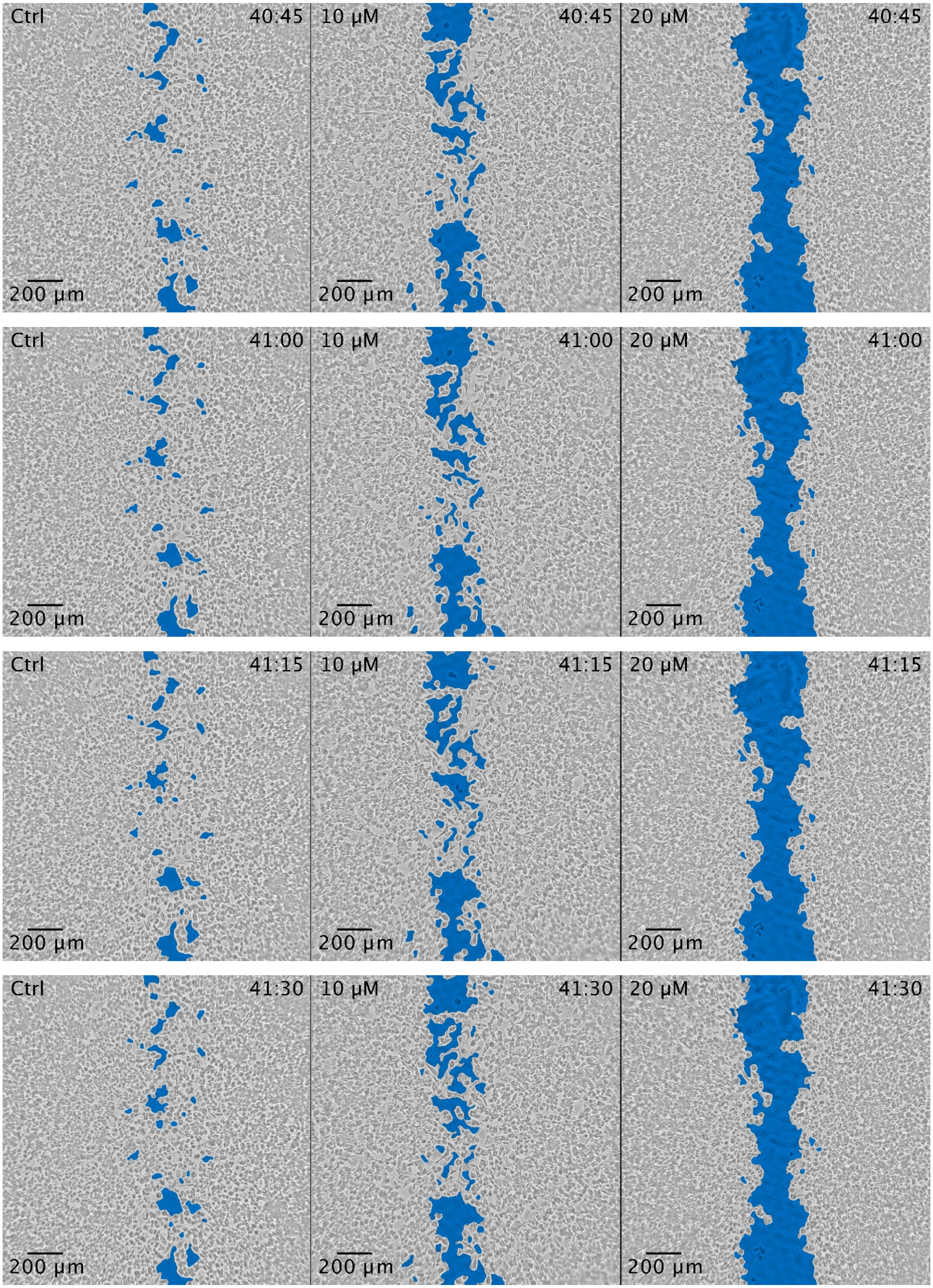
Ctrl
40:45
10 μM
40:45
20 μM
40:45
200 μm
200 μm
200 μm
Ctrl
41:00
10 μM
41:00
20 μM
41:00
200 μm
200 μm
200 μm
Ctrl
41:15
10 μM
41:15
20 μM
41:15
200 μm
200 μm
200 μm
Ctrl
41:30
10 μM
41:30
20 μM
41:30
200 μm
200 μm
200 μm

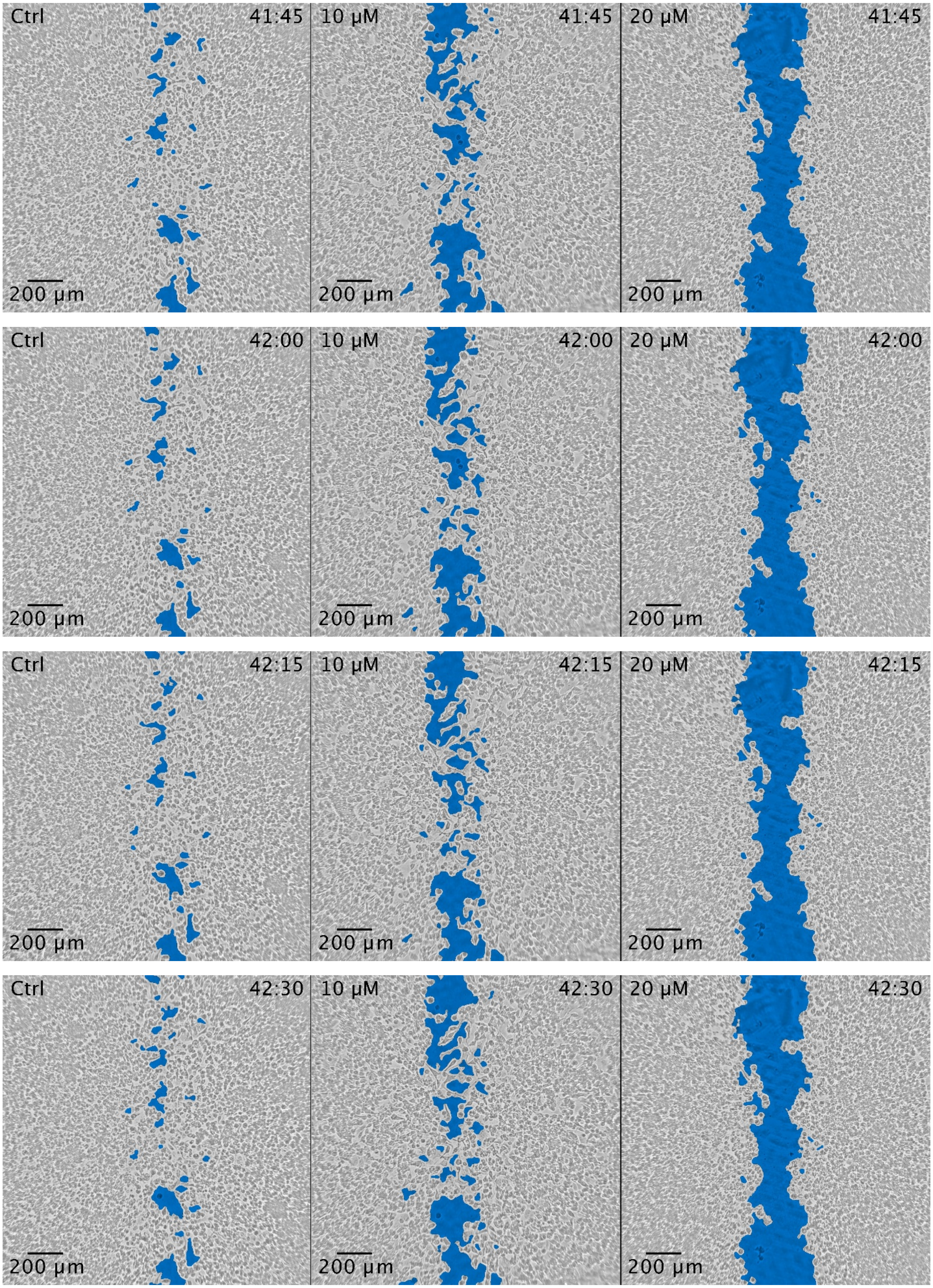
Ctrl
41:45
10 µM
41:45
20 µM
41:45
200 µm
Ctrl
42:00
10 µM
42:00
20 µM
42:00
200 µm
Ctrl
42:15
10 µM
42:15
20 µM
42:15
200 µm
Ctrl
42:30
10 µM
42:30
20 µM
42:30
200 µm

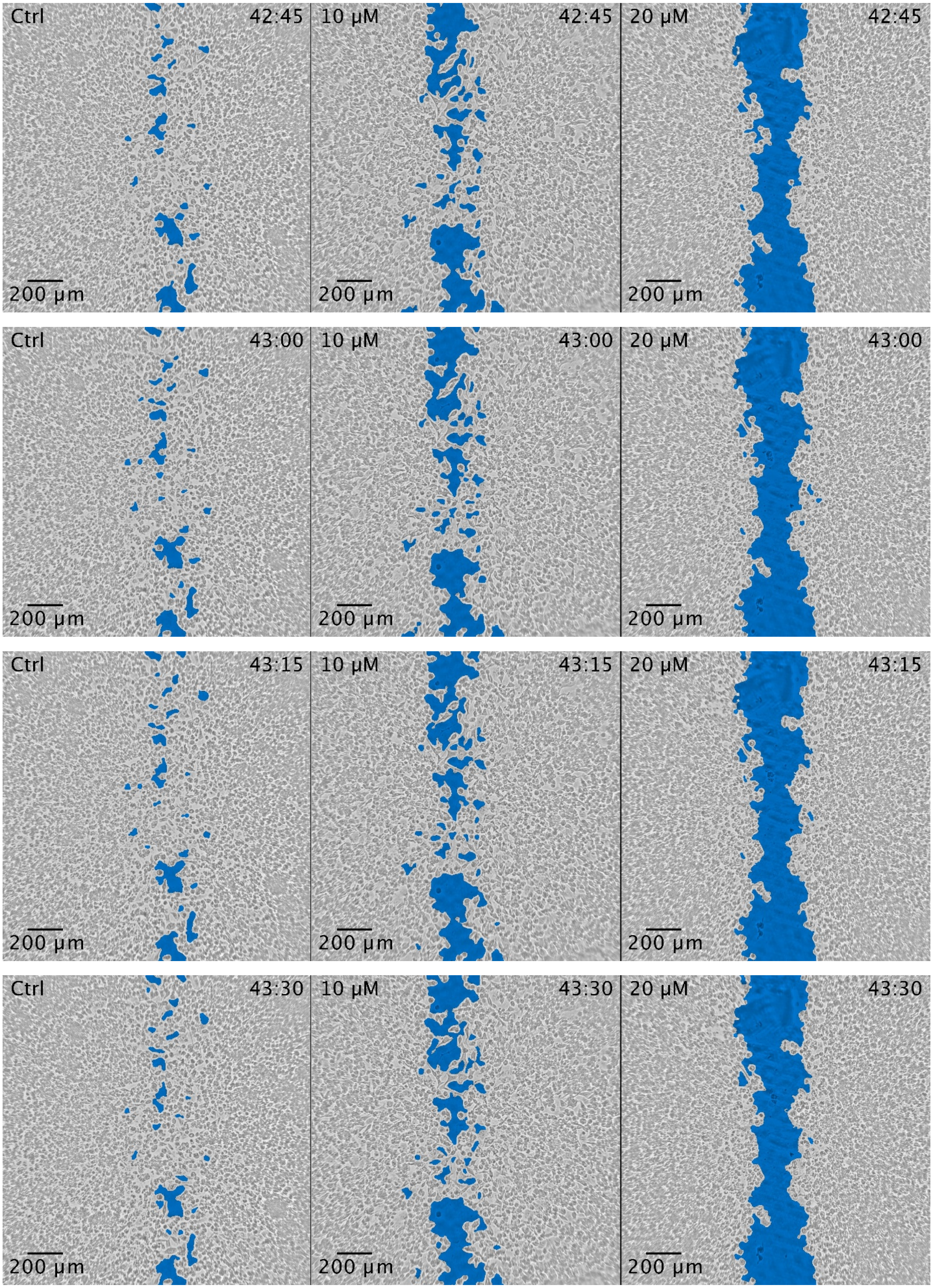

Ctrl
42:45
10 μM
42:45
20 μM
42:45
200 μm
Ctrl
43:00
10 μM
43:00
20 μM
43:00
200 μm
Ctrl
43:15
10 μM
43:15
20 μM
43:15
200 μm
Ctrl
43:30
10 μM
43:30
20 μM
43:30
200 μm

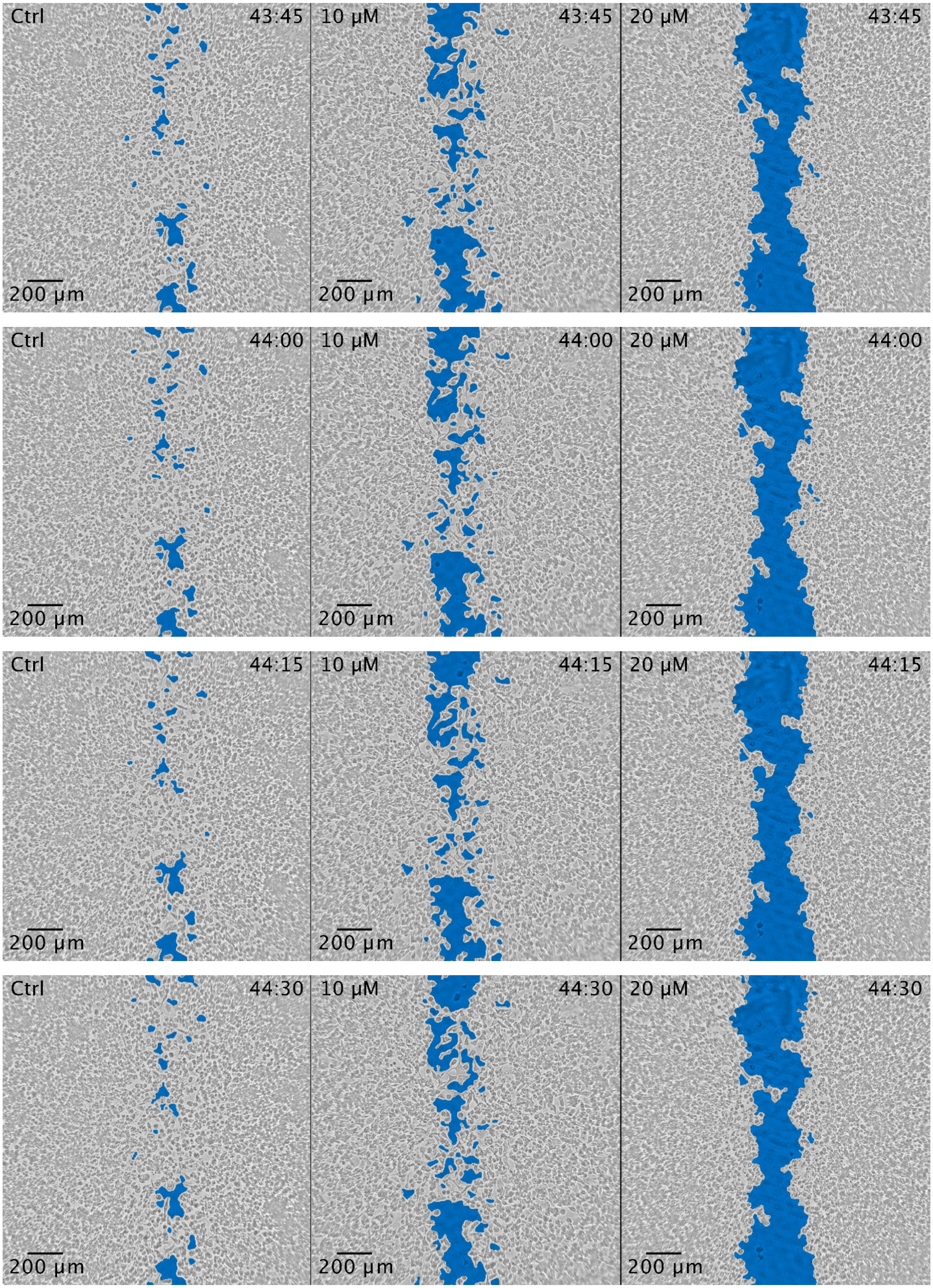
Ctrl
43:45
10 μM
43:45
20 μM
43:45
200 μm
Ctrl
44:00
10 μM
44:00
20 μM
44:00
200 μm
Ctrl
44:15
10 μM
44:15
20 μM
44:15
200 μm
Ctrl
44:30
10 μM
44:30
20 μM
44:30
200 μm

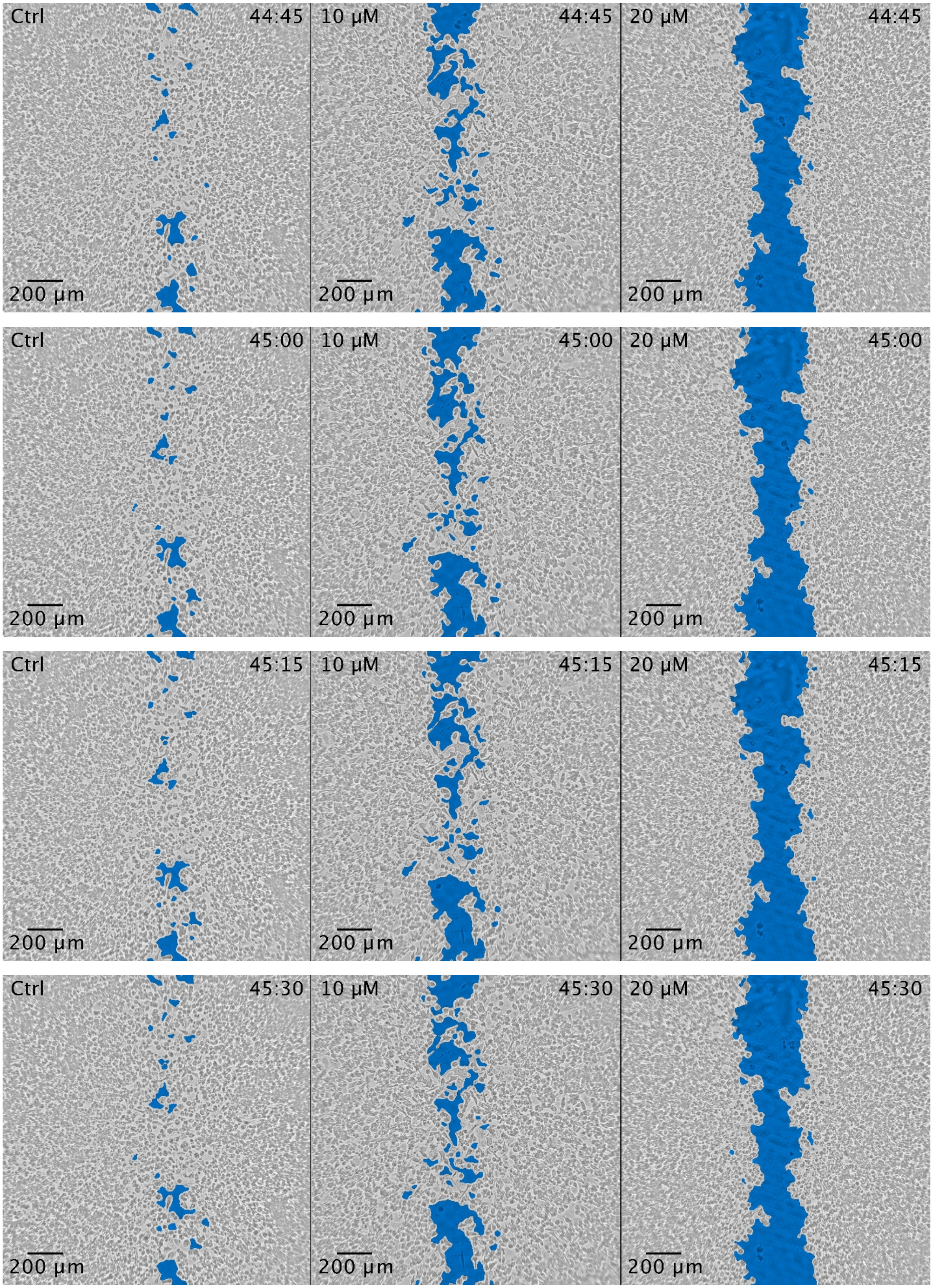
Ctrl
44:45
10 µM
44:45
20 µM
44:45
200 µm
Ctrl
45:00
10 µM
45:00
20 µM
45:00
200 µm
Ctrl
45:15
10 µM
45:15
20 µM
45:15
200 µm
Ctrl
45:30
10 µM
45:30
20 µM
45:30
200 µm

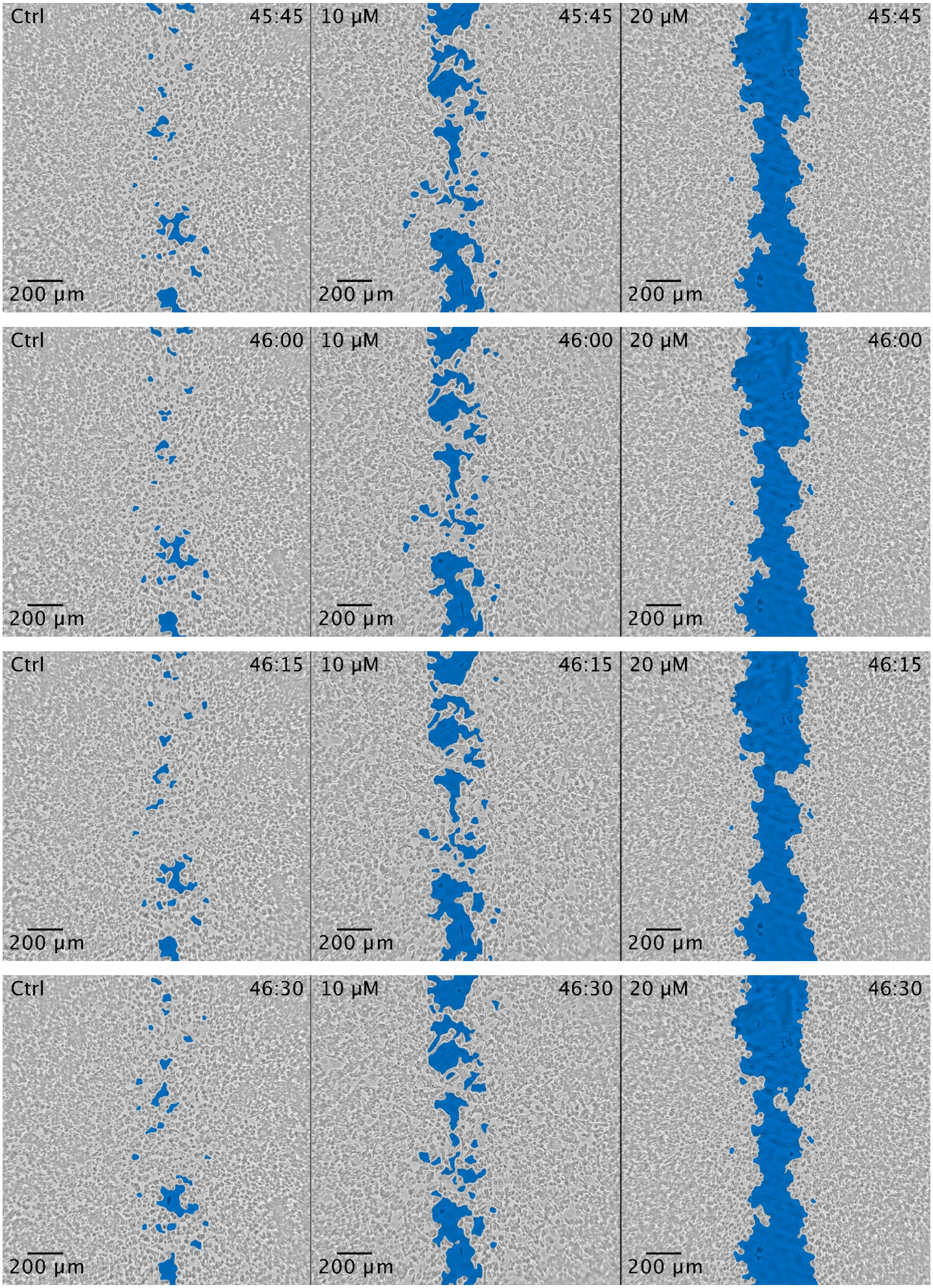
Ctrl
45:45
10 µM
45:45
20 µM
45:45
200 µm
Ctrl
46:00
10 µM
46:00
20 µM
46:00
Ctrl
46:15
10 µM
46:15
20 µM
46:15
Ctrl
46:30
10 µM
46:30
20 µM
46:30

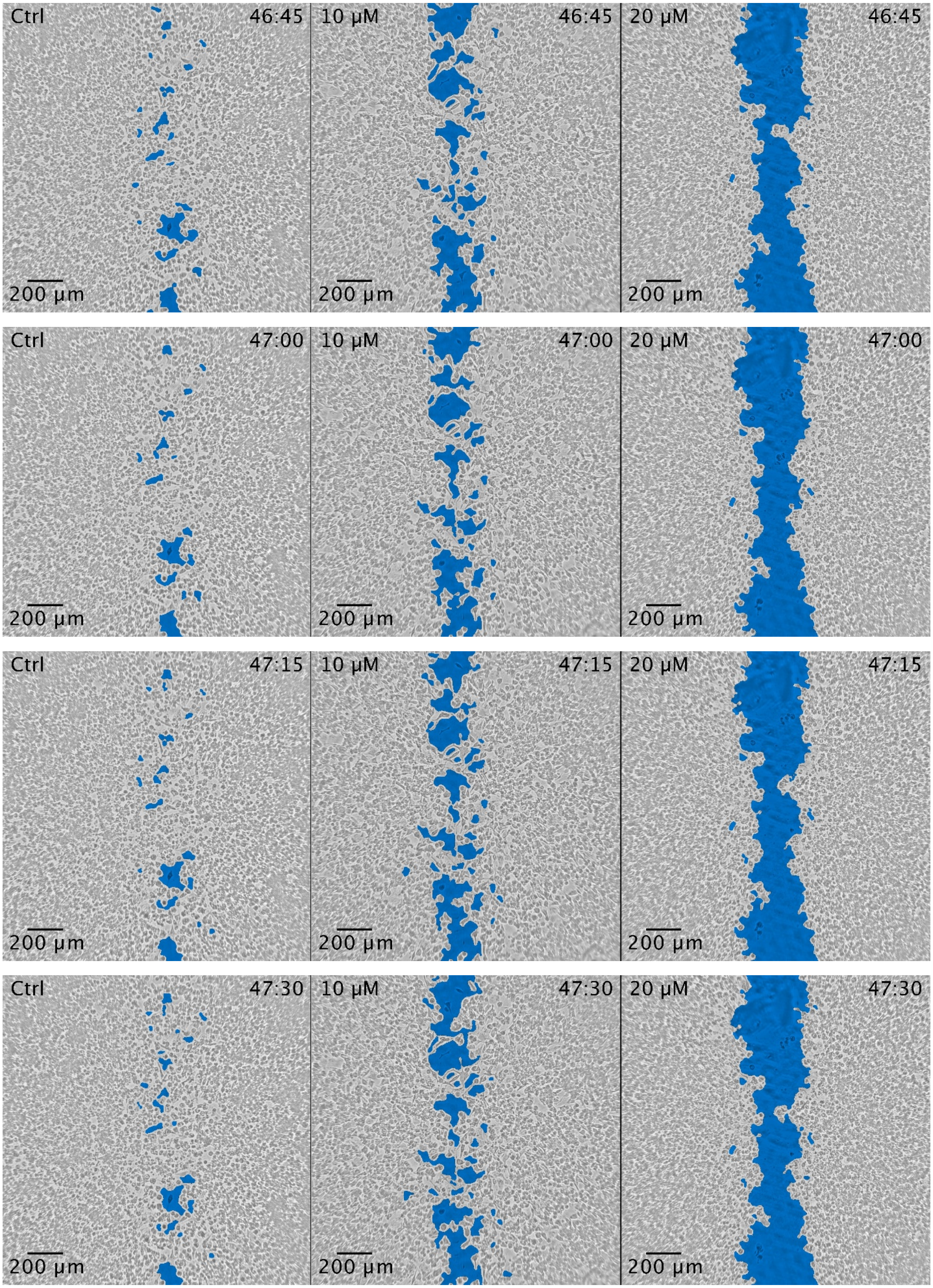

Ctrl
46:45
10 µM
46:45
20 µM
46:45
200 µm
200 µm
200 µm
Ctrl
47:00
10 µM
47:00
20 µM
47:00
200 µm
200 µm
200 µm
Ctrl
47:15
10 µM
47:15
20 µM
47:15
200 µm
200 µm
200 µm
Ctrl
47:30
10 µM
47:30
20 µM
47:30
200 µm
200 µm
200 µm

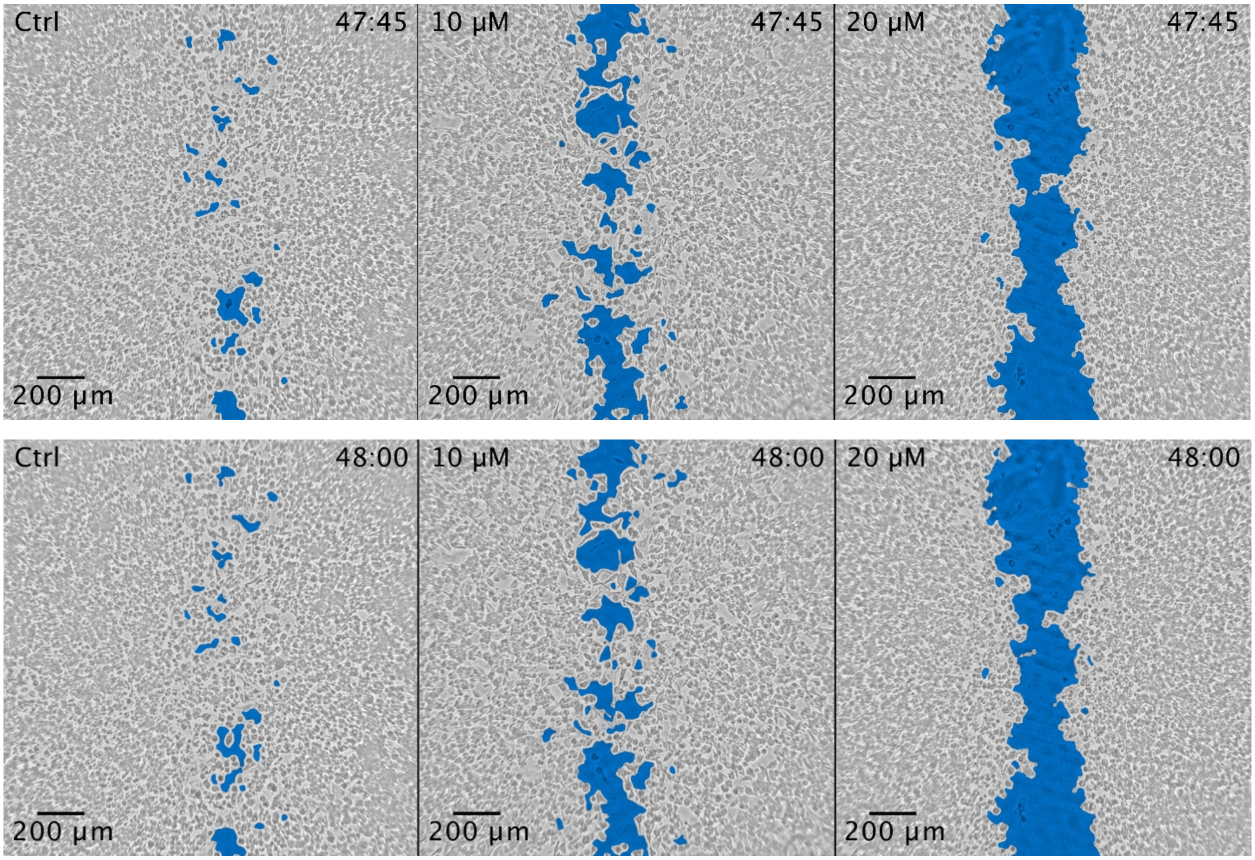
Ctrl
47:45
200 µm
10 µM
47:45
200 µm
20 µM
47:45
200 µm
Ctrl
48:00
200 µm
10 µM
48:00
200 µm
20 µM
48:00
200 µm

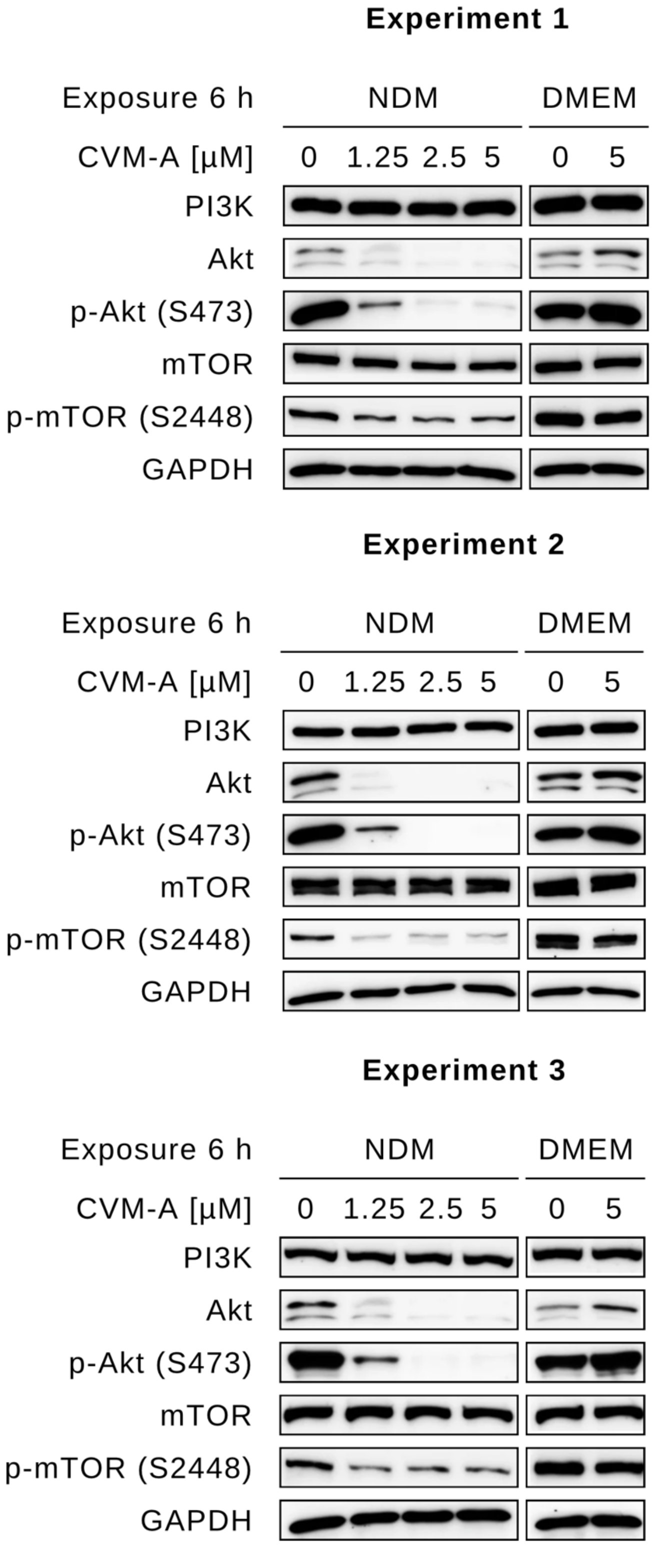

Figure S5. Three independent experiments of calliviminone A(CVM-A)-mediated modulation of the PI3K/Akt/mTOR signaling pathway in PANC-1 pancreatic cancer cells in NDM and DMEM.

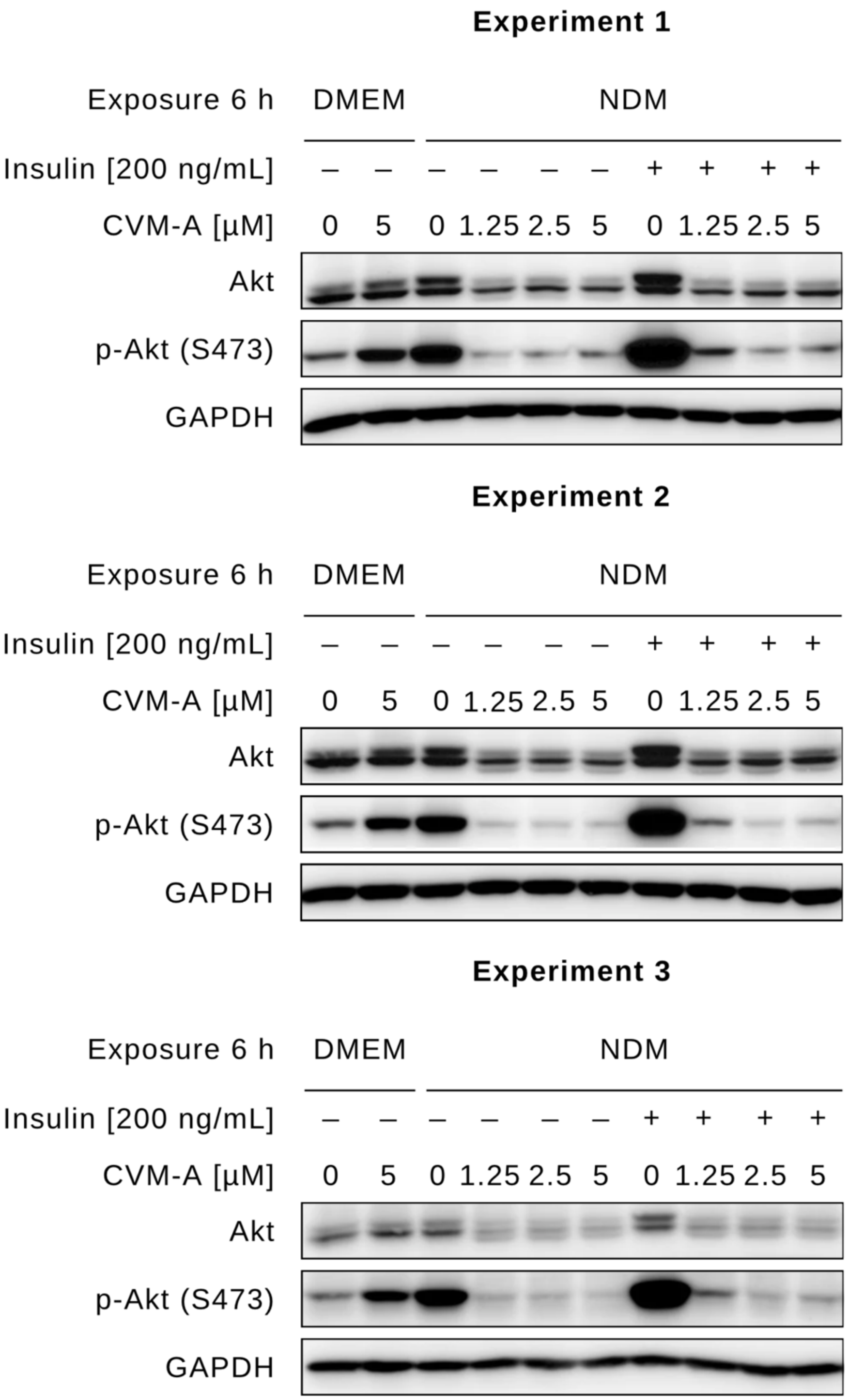

Figure S6. Three independent experiments of calliviminone A (CVM-A) on insulin-induced Akt activation in NDM.
